# Population genetic analysis of the microsporidium *Ordospora colligata* reveals the role of natural selection and phylogeography on its extremely compact and reduced genome

**DOI:** 10.1093/g3journal/jkad017

**Published:** 2023-01-19

**Authors:** Pascal Angst, Dieter Ebert, Peter D Fields

**Affiliations:** Department of Environmental Sciences, Zoology, University of Basel, Basel 4051, Switzerland; Department of Environmental Sciences, Zoology, University of Basel, Basel 4051, Switzerland; Department of Environmental Sciences, Zoology, University of Basel, Basel 4051, Switzerland

**Keywords:** population genomics, co-phylogeography, microsporidia, fungi, *Daphnia*, mutation rate

## Abstract

The determinants of variation in a species’ genome-wide nucleotide diversity include historical, environmental, and stochastic aspects. This diversity can inform us about the species’ past and present evolutionary dynamics. In parasites, the mode of transmission and the interactions with the host might supersede the effects of these aspects in shaping parasite genomic diversity. We used genomic samples from 10 populations of the microsporidian parasite *Ordospora colligata* to investigate present genomic diversity and how it was shaped by evolutionary processes, specifically, the role of phylogeography, co-phylogeography (with the host), natural selection, and transmission mode. Although very closely related microsporidia cause diseases in humans, *O. colligata* is specific to the freshwater crustacean *Daphnia magna* and has one of the smallest known eukaryotic genomes. We found an overlapping phylogeography between *O. colligata* and its host highlighting the long-term, intimate relationship between them. The observed geographic distribution reflects previous findings that *O. colligata* exhibits adaptations to colder habitats, which differentiates it from other microsporidian gut parasites of *D. magna* predominantly found in warmer areas. The co-phylogeography allowed us to calibrate the *O. colligata* phylogeny and thus estimate its mutation rate. We identified several genetic regions under potential selection. Our whole-genome study provides insights into the evolution of one of the most reduced eukaryotic genomes and shows how different processes shape genomic diversity of an obligate parasite.

## Introduction

Understanding a species’ genome-wide nucleotide diversity requires information about historical conditions (e.g. phylogeography and population structure), environmental conditions (e.g. adaptation to local climate), and stochastic processes (e.g. genetic drift and founder events; Marske *et al*. 2020; Horníková *et al*. 2021; Font-Porterias *et al*. 2021). The contribution of each factor to the observed genomic diversity is highly variable. For example, in species prone to small population sizes, alleles may reach fixation solely due to genetic drift (Leroy *et al*. 2021). In contrast, in larger populations, natural selection is more efficient in preventing deleterious mutations from being fixed and in deterministically increasing the frequency of beneficial alleles. For parasites, hosts are part of the environment, with interactions between host and parasite possibly having profound impact on the parasite's genomic diversity (Ebert and Fields 2020). A distinct feature of parasite life history is its mode of transmission. A parasite can be transmitted vertically (from parent to offspring), horizontally (no parent–progeny relationship between hosts) or mixed-mode (featuring vertical and horizontal transmission; Chrostek *et al*. 2017). The genetic diversity of specific regions under selection, or genome-wide allele frequencies might change due to the parasite's mode of transmission (Russell *et al*. 2020). Notably, the acquisition of vertical transmission by horizontally transmitted parasites was suggested to increase the potential for stochastic processes to produce random changes in the genome, because with vertical transmission population bottlenecks become more likely and thus effective population sizes, *N_e_*, would be reduced (Haag *et al*. 2020). Disentangling the effects of parasite-specific life histories from more general processes on a genomic level is still a challenge in the field of evolutionary genomics.

Microsporidia are a clade of intracellular parasites that is characterized by high variation in many aspects of life history and genomics. They cause diseases commonly referred to as microsporidiosis in agriculturally important animals, honeybees, and immunocompromised humans, among others (Buczek *et al*. 2020; Wadi and Reinke 2020) and are reported to infect even unicellular organisms (Murareanu *et al*. 2021). A growing body of research features them as a model clade for understanding evolutionary processes related to intracellular parasitism (Wadi and Reinke 2020; Murareanu *et al*. 2021). Microsporidia are phylogenetically associated with the fungi, but show high specialization to an intracellular life-style combined with a large variation in life histories (Corradi 2015). Most microsporidia do not have mitochondria but take up energy from their host using transmembrane transporters, although a few species have maintained mitochondria (e.g. Haag *et al*. 2014). Their genome size (about 2–50 Mb) and number of genes (about 2,000–4,000) vary extremely (Wadi and Reinke 2020), but microsporidia generally have a small number of genes and small genome size, including one of the smallest known eukaryotic genomes (Pombert *et al*. 2015). Recent work suggests that large genomes evolved in microsporidia due to the accumulation of repetitive elements when the strength of purifying selection decreases (de Albuquerque *et al*. 2020). For example, large, gene-sparse genomes evolved in the microsporidian genera *Hamiltosporidium* and *Nosema* after a switch from horizontal transmission to mixed-mode transmission, which reduced *N_e_* (Haag *et al*. 2020). However, only ∼18% of 1,440 described species are potentially vertically transmitted (Murareanu *et al*. 2021), while the more common exclusive horizontal transmission should allow for large *N_e_* and the maintenance of the typical streamlined, i.e. reduced and compact, genomes.

Hosts of several microsporidia are planktonic freshwater crustacea of the genus *Daphnia*, well-established model systems in ecology, evolution, and in the study of host–parasite interactions (Altermatt and Ebert 2008; Ebert 2008; Orlansky and Ben-Ami 2019). Several microsporidia that infect *D. magna* have been studied both ecologically, as well as on the genomic level, which is important when ecological scenarios are used to explain genome evolution. Examples include the microsporidium *Hamiltosporidium tvaerminnensis*, a microsporidium with a large genome size and mixed-mode of transmission (Ebert *et al*. 2001; Decaestecker *et al*. 2005; Goren and Ben-Ami 2013; Angst *et al*. 2022), and several microsporidian gut parasites—e.g. *Glugoides intestinalis*, *Mitosporidium daphniae*, and *Ordospora colligata*—that have small genomes, look superficially similar under the microscope, infect the same host tissue, and are of relatively low virulence to the host (Larsson *et al*. 1996, 1997; Haag *et al*. 2014). However, the three mentioned *D. magna* gut microsporidia diverge substantially on the genetic level (Williams *et al*. 2018). *Ordospora colligata* has received a lot of attention because it has one of the most reduced genomes across all eukaryotes (∼2.3 Mb) and is closely related to the microsporidian genus *Encephalitozoon* that primarily infects humans and other mammals (Pombert *et al*. 2015). This species features in several ecological and evolutionary studies (e.g. Ebert *et al*. 2000; Refardt and Ebert 2007; Kirk *et al*. 2019; Manzi *et al*. 2021), some of which suggest the species is adapted to colder habitats (Kirk *et al*. 2018). Compared with, for example, *H. tvaerminnensis*, *O. colligata* is suggested to exhibit large population sizes that may be a key factor for maintaining its streamlined genome (Haag *et al*. 2020). Importantly, *O. colligata* is transmitted entirely horizontally (Ebert 2005), and little is known about the evolution of exclusively horizontally transmitted microsporidian parasites.

In this population genomic study, we characterize genomic diversity across the species range of *O*. *colligata*. We aimed to understand the relative contribution of phylogeography, selection, and mode of transmission to shaping the variation in nucleotide diversity. The phylogeography of *D. magna*, the only known host of *O. colligata*, shows three main lineages and isolation by distance (IBD; Fields *et al*. 2015, 2018; Andras *et al*. 2018; Bekker *et al*. 2018). Based on neutral assumptions, we expected a shared phylogeography between host and obligate parasite, i.e. co-cladogenesis. Furthermore, because of *O*. *colligata*'s horizontal mode of transmission and its streamlined genome, we expected to find a constant, large population size, allowing for efficient selection. We expected to find a higher selection efficacy in *O*. *colligata* than previously published in *Hamiltosporidium*, which would support the hypothesis by Haag *et al*. (2020) that the mode of transmission is a driving factor for the evolution of genome size in microsporidia. At least in part, our population genomic study corroborates earlier speculation about mechanisms driving the evolution of strongly diverged genome sizes in microsporidia by investigating the relative importance and interplay of biologically meaningful processes shaping the variation in genomic diversity.

## Materials and methods

### *Daphnia magna* diversity panel

We used parasites derived from material collected within the framework of a large-scale biogeographic study of the host species, *D. magna* (Fields *et al*. 2015, 2018, 2022; Seefeldt and Ebert 2019). From each population, animals were brought to the laboratory and one iso-female line, i.e. clone, was created. We checked these clones for infections with microsporidia by phase-contrast microscopy, using squash-preparations or samples of the gut. The panel includes whole-genome sequencing of *D. magna* clones with illumina paired-end reads using HiSeq 2500 and NovaSeq 6000 sequencers.

### Samples

Whole-genome sequences of 10 *O. colligata* samples, each from a different *D. magna* clone collected from a different population, were obtained from the illumina sequencing (Table 1). Sequences from clones FI-SK-17-1, NO-V-7, and GB-EP-1 were reused from Haag *et al*. (2020) (NCBI database; SRA accession: SRP211974, Bioproject ID: PRJNA419750), sequences from clone RU-BAYA1-1 were reused from Angst *et al*. (2022) (NCBI database; SRA accession: SRP346323, Bioproject ID: PRJNA780787), and for clone FI-SK-17-1 additional sequencing was done. We used the *D. longispina*-specific *Ordospora pajunii* for analyses that needed an outgroup (NCBI database; Bioproject ID: PRJNA630072; de Albuquerque *et al*. 2022). Some samples with observed microsporidia gut infection (i.e. based on published criteria for identifying *O. colligata* infection) showed <1× average whole-genome coverage for *O. colligata*. To verify the presumed parasite at species level, we performed polymerase chain reactions (PCRs) of the small subunit of microsporidian ribosomal DNA followed by Sanger sequencing (Supplementary Table 1 and Methods).

**Table 1.** Sample information.

Sample ID	Country	Latitude	Longitude	Coverage
CN-WON-2	China	46.825017	124.378953	10×
FI-SK-17-1	Finland	59.8312191	23.2577045	19×
FI-SKW-2-1	Finland	59.8329692	23.2562571	26×
GB-EP-1	Great Britain	52.4535830	−1.9194440	71×
NO-V-7	Norway	67.68696	12.67197	70×
RU-BAYA1-1	Russia	53.017833	106.886833	29×
RU-KU1-2	Russia	55.303833	63.405333	21×
US-SP131-1	United States	44.3332	−68.0632	40×
US-SP15-1	United States	44.3331	−68.0629	55×
US-SP163-1	United States	44.3336	−68.0636	37×

*Ordospora colligata*'s average whole-genome coverage is denoted in the last column.

### Read mapping

We assessed raw sequencing reads for quality using FastQC v.0.11.7 (http://www.bioinformatics.babraham.ac.uk/projects/fastqc) and subsequently trimmed them to remove low-quality sequences and adapter contamination using Trimmomatic v.0.38 (Bolger *et al*. 2014). Unless otherwise stated, we used default parameters for bioinformatics programs. We assayed successful trimming using a second run of FastQC. We downloaded the genome assembly of *O. colligata* (NCBI database; Assembly name: ASM80326v; GenBank assembly accession: GCA_000803265.1, Bioproject accession: PRJNA210314; Pombert *et al*. 2015) from NCBI and used it as a reference for mapping quality-trimmed reads with BWA MEM v.0.7.17 (Li 2013), using default parameters. We converted SAM files to BAM files, coordinate-sorted individual BAM files, and removed unmapped reads using SAMtools v.1.9 (Li *et al*. 2009). Afterward, we added read groups and marked duplicates for individual BAM files using Picard Toolkit v.2.18.16 (Broad Institute 2020) and computed the average read depth using SAMtools function depth.

### Ploidy

We applied two different methods to infer the ploidy of *O. colligata* samples. First, we used ploidyNGS v.3.1.2 (Augusto Corrêa dos Santos *et al*. 2017) to visualize frequencies of putative alleles directly from individual BAM files. Second, we output reads which mapped to the reference genome to FASTQ files using BEDtools v.2.27.1 (Quinlan 2014) and subsequently used Kmercountexact.sh, a bash script included in BBMap v.38.22 (Bushnell 2016) to generate k-mer frequency histograms. Based on the results of this ploidy assessment, which were consistent across the two methodologies, *O. colligata* was treated as haploid in all analyses requiring ploidy assignment.

### Variant calling

For variant calling, we used GATK v.3.8 HaplotypeCaller in the haploid mode (McKenna *et al*. 2010; Van der Auwera *et al*. 2013). Specifically, we first generated GVCFs for individual BAM files and used the GATK function GenotypeGVCF to combine GVCFs into an all-site VCF. We filtered this VCF file to include only SNP variants (i.e. exclude INDEL variants) using VCFtools v.0.1.16 (Danecek *et al*. 2011).

### Coding sequences—single-copy orthologs

Phylogenetic analyses and tests for selection (especially its efficacy) mostly rely on protein-coding regions. To characterize variation in nucleotide diversity within the protein-coding sections of the *O. colligata* samples, we needed to extract subsets of the genome-wide VCF using the following approaches. Protein sequences of *O. colligata* (OC4; GenBank accession: JOKQ00000000.1) were downloaded from NCBI and complemented with those of the outgroup, *O. pajunii* (GenBank accession: JACCJH000000000.1; de Albuquerque *et al*. 2022). To find one-to-one orthologs between the species, we used protein datasets of the two as input for OrthoMCL v.2.0.9 (Li *et al*. 2003). Specifically, we followed the automated pipeline described at the following Github repository: https://github.com/apetkau/orthomcl-pipeline. We aligned the identified orthologous sequences of *O. colligata* and *O. pajunii* with PRANK v.170427 (Löytynoja 2014) using a custom script adapted from (Fields *et al*. 2022). After an initial survey of pairwise alignment quality, we implemented a masking step, wherein excessively divergent or poorly aligned sequences (divergence > 0.5%) were excluded from downstream analysis. We used the R package *seqinR* v.3.4-5 (Charif and Lobry 2007) for importing FASTA alignments and *PopGenome* v.2.7.1 (Pfeifer *et al*. 2014) for the manipulation of the VCF file in the next step. To produce multiple sequence alignments, we generated one alternative reference for each *O. colligata* sample using the GATK-functions SelectVariants and FastaAlternateReferenceMaker. We cut out coding sequences of interest from the alternative references with the script gff2fasta.pl (https://github.com/ISUgenomics/common_scripts). To align the resultant coding sequences of the *O. colligata* ingroup samples to the *O. colligata–O. pajunii* alignments, we used MAFFT v.7.407 (Katoh *et al*. 2002; Katoh and Standley 2013) and its –add option. Next, we masked all positions previously masked in the two species reference alignments in the multiple sequence-alignments using generate_masked_ranges.py (https://gist.github.com/danielecook) and BEDtools function maskfasta.

### Sequence variation and population genetic analyses

For calculating the average number of nucleotide differences (π) between *O. colligata* genomes, we used pixy v.0.95 (Korunes and Samuk 2021). Beforehand, we filtered out alleles with less than half or more than double the average sample coverage. Finally, we averaged the estimated π-values over one Kilobase pair (kb) windows. Similarly, we calculated the non-/synonymous per-site nucleotide diversity (π_N_ and π_S_) for the coding sequences of *O. colligata* using the script selectionStats.py (https://github.com/tatumdmortimer/popgen-stats). Both methods are consistent with theoretical expectations and comparable among species as they take invariant sites into account for their calculations (Korunes and Samuk 2021). The ratio of π_N_ and π_S_ was separately calculated for BUSCO genes (Benchmarking Universal Single-Copy Orthologs) identified using BUSCO v.4.0.1 (Seppey et al. 2019) and its microsporidia_odb10 database (Creation date: August 05, 2020) and compared with published π_N_/π_S_ values of *H. tvaerminnensis* (Angst *et al*. 2022).

### Population structure and phylogenetic analyses

To assess population structure in *O. colligata*, we used the R v.3.5.1 (R Core Team 2018) packages *SNPRelate* v.1.14.0 and *gdsfmt* v.1.16.0 (Zheng *et al*. 2012) for principal component analysis (PCA) as well as cluster analysis with the whole-genome polymorphism data. With the same data, we also estimated the maximum likelihood of sample ancestries using ADMIXTURE v.1.3.0 (Zhou *et al*. 2011) with ten replicates for both *K* = 2 and *K* = 3. For this analysis, we prepared the input using PLINK v1.90b6.21 (Chang *et al*. 2015) and the outputs were visually summarized using pong v.1.5 (Behr *et al*. 2016). Additionally, to test for IBD, we compared the samples’ pairwise relatedness with their pairwise geographic distance. Therefore, we used the R package *hierfstat* v.0.5-7 (Goudet and Thibaut 2020) to calculate the pairwise relatedness and *geodist* v.0.0.3 (Padgham and Sumner 2019) to calculate the geographic distance between samples with the geodesic measure. We measured the distance to the North American samples across the Bering Strait to account for the likely dispersal route of host and parasite given their population structure. For file import and format conversions, we used *VCFR* and *adegenet* v.2.1.2 (Jombart 2008) in R. We tested for association between relatedness and geographic distance using distance-based Moran's eigenvector maps and redundancy analysis (dbMEM analysis by RDA). Specifically, we transformed the explanatory variable, geographic distance, into dbMEMs using the *adespatial* v.0.3-14 (Dray *et al*. 2021) R package and decomposed the response variable, genetic relatedness, into principal components using the R base *stats* function prcomp. We then used the R package *vegan* v.2.5-7 (Oksanen *et al*. 2020) for RDA and assessed significance with 1,000 permutations.

In addition to looking at whole-genome diversity, we also focused directly on the phylogenetic signal in protein-coding regions of the genome. We concatenated previously extracted single-copy ortholog gene alignments into a single sequence and filtered for 4-fold degenerate sites with MEGA v.7.16.0617 (Kumar *et al*. 2016), which we used as input for Bayesian phylogenetic analysis in BEAST2 v.2.6.2 (Drummond *et al*. 2005; Bouckaert *et al*. 2019). We prepared inputs using the graphical user-interface application BEAUti, which is part of BEAST2. We used the GTR + G substitution model, because this was the most likely model for our data according to jModelTest v.2.1.10 (Darriba *et al*. 2012), a strict clock, the Yule model as tree prior, and a MCMC chain of 10,000,000 iterations. After observing a clear split between Western Eurasian and East Asian (plus North American) samples, we further used a most recent common ancestor prior with a log-normal distribution for this split and switched to a calibrated Yule model. Specifically, we used *M* = 1, *S* = 0.775, and offset = 0.45 for the log-normal distribution which translates to a median of 3.17 MYA and a 95% C.I. of 1.21–10.2 MYA and reflects the likely range for this split in the host (Cornetti *et al*. 2019). We investigated the convergence of the analysis using Tracer v.1.7.1 (Rambaut *et al*. 2018) and ensured that the effective sample size of the parameters was >200. For generating the final tree, the posterior sample of trees was summarized to a maximum clade credibility tree using TreeAnnotator, which is part of BEAST2, with the first 10% of the MCMC chain discarded as burn-in. Lastly, we visualized the obtained tree on a map of the Holarctic using the R package *phytools* v.0.7-20 (Revell 2012).

### Demographic history and rate of adaptive nucleotide substitutions

We applied the McDonald–Kreitman test (MKT; McDonald and Kreitman 1991) and a more recent derivate, the asymptotic MKT (Haller and Messer 2017), to estimate *α*. However, the imbalance between low intraspecific diversity and high divergence to the outgroup *O. pajunii* in the multi-sequence alignments of single-copy orthologs impeded reliable estimation of *α* (Jesús Murga-Moreno, personal communication). We refrained from estimating past and present *N_e_* using PSMC after determining that *O. colligata* is likely haploid and did not use site frequency spectrum-based methods for that because of our low sample size (overall and within lineages).

## Results

### Samples, mapping, and sequence variation

**Fig. 1.**
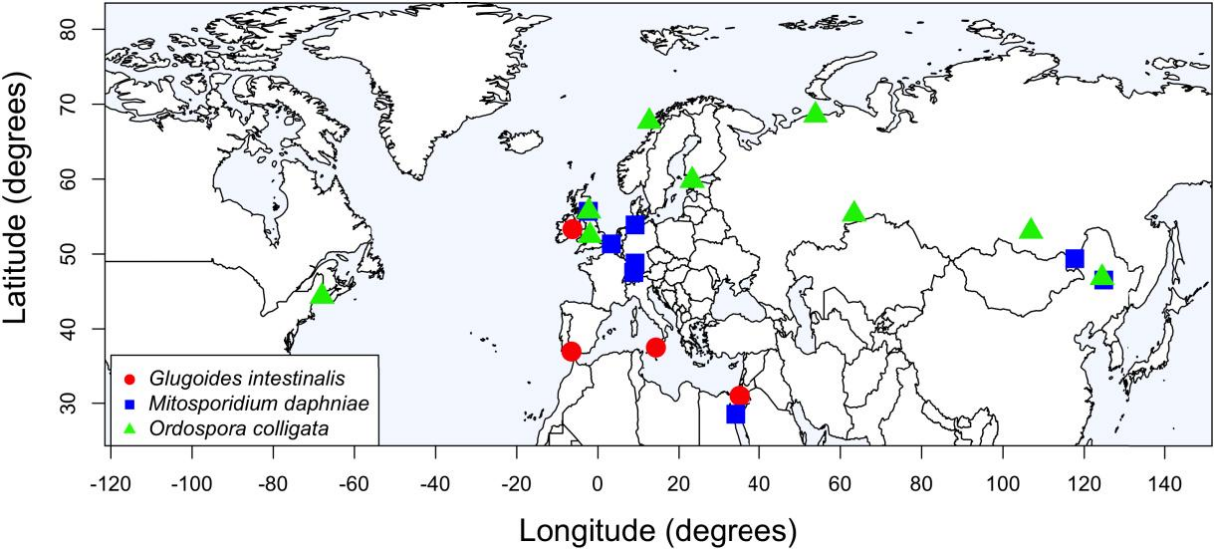
Sampling distribution of three *D. magna* gut microsporidia. Based on sampling within the framework of the *D. magna* diversity panel, we found wide-range distributions of the host species-specific microsporidia *Glugoides intestinalis* (circles), *Mitosporidium daphniae* (squares), and *Ordospora colligata* (triangles). *Ordospora colligata* has the widest distribution within the Holarctic host distribution, while the other two microsporidia have not been observed in Northern America.

The accuracy of microscopy in identifying *D. magna* microsporidian gut parasites on a species level was not as high as we had assumed. By using the combination of microscopy and PCR, we could reliably identify species, and we found that *D. magna* shows infections with microsporidian gut parasites across its Holarctic species range (Fig. 1, Supplementary Table 1). *Ordospora colligata* showed the widest geographic distribution but was absent from southern regions. Genomic samples of ten sequenced host clones sufficiently represented the *O. colligata* genome to describe its variation in genomic diversity (Table 1). The percentage of sequencing reads, originating from the combined sequencing of host and parasite, being mapped to the parasite's genome ranged from 0.22 to 2.97% for *O. colligata*. The average whole-genome coverage for *O. colligata* was >10× in all samples. We found a total number of 12,427 SNPs and a whole-genome estimate of π based on 1 kb windows of 0.003, approximately half a percent.

**Fig. 2.**
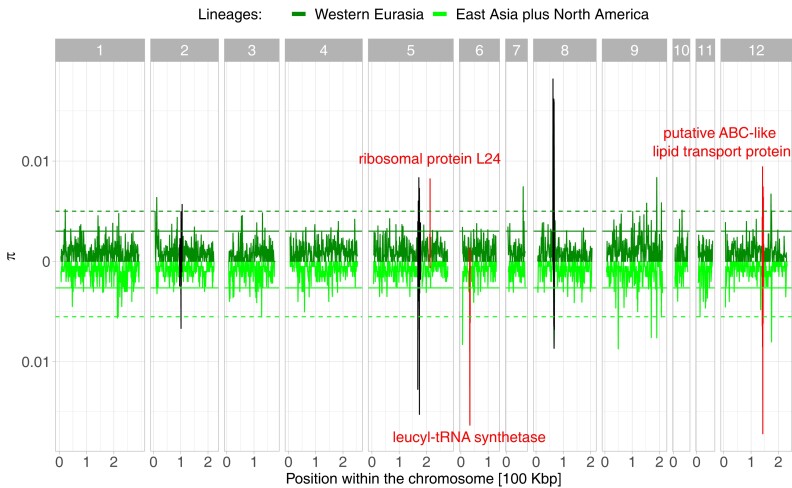
Nucleotide diverstiy (π) in *O. colligata*. Per-site π is averaged over 1 kb windows along the genome. The π values of both main *O. colligata* lineages are presented in 12 facets corresponding to the parasite's 12 chromosomes. We used the combined sample of East Asian and North American samples to have equal sample sizes for comparing lineages (*N* = 5). The 95th and 99th percentiles of each lineage are indicated with a solid and a dashed line, respectively. The first and last 5 kb per chromosome were masked for better presentation. Black windows indicate increased diversity at three genetic regions due to host-to-parasite horizontal gene transfer and the associated miss-mapping of sequencing reads (i.e. host reads mapping to the parasite's genome assembly). Annotated genes with increased π values mentioned in the discussion are indicated in red with their locus IDs: the ribosomal protein L24 (M896_051540), a leucyl-tRNA synthetase (M896_060290), and a putative ABC-like lipid transport protein (M896_121220).

The *O. colligata* genome has at least three regions that were horizontally transferred from the host (Pombert *et al*. 2015). Windows overlapping with these regions showed increased diversity due to unintentional mapping of *D. magna* reads (Fig. 2). Other windows within the 99th percentiles for π overlapped with functionally annotated genes [locus IDs as adopted from Pombert *et al*. (2015): the ribosomal protein L24 (M896_051540), a leucyl-tRNA synthetase (M896_060290), and a putative ABC-like lipid transport protein (M896_121220)], of which a putative ABC-like lipid transport protein additionally had the fourth highest π_N_/π_S_ ratio, i.e. it might experience strong positive or balancing selection (Supplementary Table 2). The remaining windows within the 99th percentiles for π did not overlap with annotated genes.

### Ploidy

**Fig. 3.**
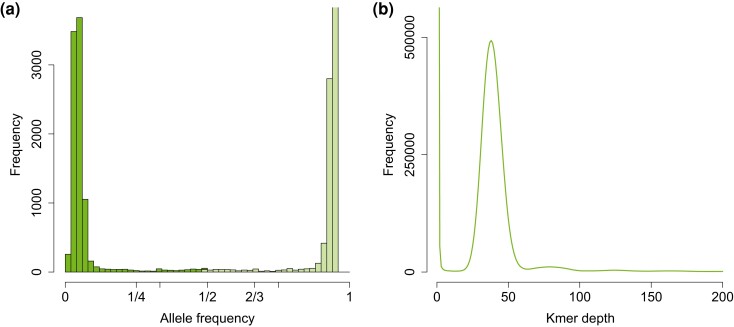
Graphical summaries of the two approaches to determine ploidy levels for *O. colligata* sample US-SP15-1. Histogram of allele frequency of putative alleles in the BAM file found using ploidyNGS (a), where reads supported a single variant for most positions. Few sites and no sharp central peak at intermediate allele frequencies are seen as would be the case in diploids. Dark and light shadings of green indicate reference and alternative allele, respectively. K-mer frequency histogram inferred using kmercountexact.sh (b), with a single peak for homozygous sites and no secondary peaks which would indicate di-/polyploidy.

Allele frequency signatures of *O. colligata* samples showed haploid characteristics in 8 out of 10 samples (Fig. 3, Supplementary Figs. 1 and 2). The other two were not as clear due to low levels of polymorphisms or potential multiple infections of *O. colligata*. Specifically, no prominent peaks were found in the putative allele frequency histogram calculated using ploidyNGS. One peak can be seen in the k-mer histogram around the average whole-genome coverage. Therefore, the trimmed sequencing reads supported a single variant for most positions along the genome and no signs of heterozygosity were visible, hence *O. colligata* was treated as haploid in all analyses.

### Population structure

**Fig. 4.**
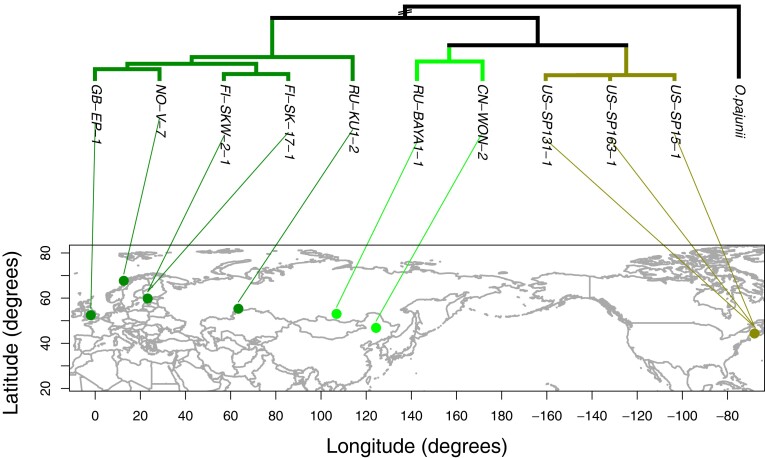
Geophylogeny of *O. colligata*. The tree is based on 4-fold degenerate sites of single copy orthologs when compared with the outgroup, *O. pajunii*. Tree tips are connected to the sampling locality by colored lines, whereby colors indicate the main lineages inferred from population structure analyses: Western Eurasian, East Asian, and North American. See Supplementary Fig. 6 for the correct branch length to *O. pajunii*, as it is out of scale here for better presentation.

**Fig. 5.**
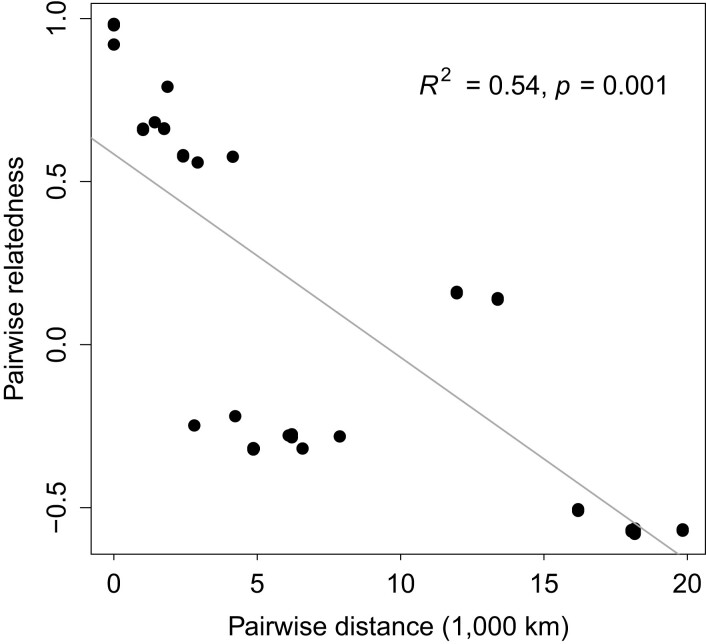
Isolation by distance (IBD). Pairwise relatedness is negatively correlated with geographic distance. Each point represents the comparison between two samples. Statistics derive from dbMEM analysis by RDA. The gray line is a regression line based on a linear model.

To determine the shared amounts of genomic variation between *O. colligata* samples, we used PCA on the whole-genome SNP data. PC1 separated samples in three clusters, which became even clearer in combination with PC2 (Supplementary Fig. 3). The two PCs explained a substantial amount of genomic variation, with 63.60 and 15.43%, respectively. The three clusters [Western Eurasian (*N* = 5), East Asian (*N* = 2), and North American (*N* = 3) cluster, named according to the geographic regions the samples in each cluster originate from] were also found in the cluster analysis, the admixture analysis, and the Bayesian phylogenetic tree estimation based on 4-fold degenerate sites (Fig. 4, Supplementary Figs. 4–6). Pairwise relatedness in *O. colligata* (Supplementary Table 3) was negatively correlated with geographic distance (Fig. 5; *R^2^* = 0.54, *P* = 0.001), suggesting IBD.

### Mutation rate estimation

After observing the same population structure in *O. colligata* as previously found in its only host, *D. magna*, we calibrated the molecular clock in the phylogenetic analysis using the fossil-calibrated divergence time between Western Eurasian and East Asian (plus North American) host samples (Cornetti *et al*. 2019). The median divergence time between *O. colligata* Western Eurasian and East Asian (plus North American) lineages given the posterior distribution from the Bayesian model was 1.61 MYA (95% HPD interval: 0.62–4.19 MYA), which is largely overlapping with the host divergence time estimate (1.3–9.3 MYA; Cornetti *et al*. 2019) used as a prior. The median clock rate estimate was 1.04 × 10^−9^ per site per year (95% HPD interval: 2.33 × 10^−10^–2.07 × 10^−9^ per site per year). Given published generation times of microsporidia (i.e. 63 h; Kramer 1965) this would correspond to a mutation rate of 7.48 × 10^−12^ per site per generation (95% HPD interval: 1.68 × 10^−12^–1.49 × 10^−11^ per site per generation). However, given its Northern distribution, *O. colligata*, like its host, endures the long winters as resting stage (spore) without reproducing. Assuming a resting time of 4–6 months, its mutation rate might be between 1.12 × 10^−11^ and 1.50 × 10^−11^ per site per generation (2.51 × 10^−12^–2.98 × 10^−11^). These values are comparable with what has previously been reported for fungi (see e.g. Zhu *et al*. 2014; Ene *et al*. 2018; Bezmenova *et al*. 2020).

### Efficient purifying selection

We assessed the efficacy of selection in *O. colligata* to test hypotheses about the evolution of the wide range of genome sizes observed in microsporidia. Therefore, we compared the ratio of nonsynonymous to synonymous polymorphisms, π_N_/π_S_, between BUSCO (*n* = 416) and non-BUSCO (*n* = 1,810) genes. The former are conserved genes among most microsporidia and are therefore expected to be under purifying selection. Indeed, BUSCO genes had a 1.5 times lower median π_N_/π_S_ ratio (0.085) than non-BUSCO genes (0.130). These values are close to an estimate for BUSCO genes in the large genome sized microsporidium *H. tvaerminnensis* (0.102). However, in *H. tvaerminnensis*, non-BUSCO genes have a three times higher median π_N_/π_S_ ratio (0.306), which is higher than all other (non-)BUSCO gene values mentioned here (Kruskal–Wallis *χ*^2^(3) = 309.83, *P* < 0.001).

## Discussion

The study of population genetics aims to understand temporal and spatial variation in genetic diversity, ideally within a framework of the ecology of the focal species. For parasites, this includes host–parasite interactions and the mode of transmission. Here, we take advantage of a model system in host–parasite evolutionary ecology, the planktonic freshwater species *D. magna*, to explain genomic diversity, genome architecture, and population structure of one of its specific parasites, the microsporidium *O. colligata*. Its host, *D. magna*, shows a clear split in its Holarctic distribution into a Western Eurasian and an East Asian (plus North American) lineage, the latter of which is subsequently divided into East Asian and North American lineages (Bekker *et al*. 2018; Fields *et al*. 2018; Cornetti *et al*. 2019). We find a pattern of co-cladogenesis with overlapping phylogeography between *O. colligata* and its host, which allows us to estimate the mutation rate of a microsporidium for the first time. Furthermore, we observe IBD, providing an explanation for the relationships among populations. We show evidence for efficient genome-wide purifying selection in *O. colligata*, which supports the hypothesis that horizontally transmitted microsporidia were able to evolve and maintain small, streamlined genomes due to high effective population sizes (Haag *et al*. 2020).

### Host–parasite co-phylogeography

The close interaction of the intracellular microsporidia and their hosts might shape the phylogeography of the parasites to be congruent to their host's phylogeography—the within-species equivalent of the Fahrenholz rule (Fahrenholz 1913), which states that host and parasite phylogenies show a pattern of co-cladogenesis. A strict co-phylogeography is expected if the parasite species’ mode of transmission is uniquely vertical (Werren *et al*. 2008), consistent with the expectation of co-dispersal as the mechanism of parasite spread (Page 2003). Previous attempts by Pelin *et al*. (2015) and Angst *et al*. (2022) to reconstruct a co-phylogeography based on whole-genome data for two microsporidian species, *Nosema ceranae* and *H. tvaerminnensis*, respectively, remained unsuccessful, possibly due to high migration (human driven in the case of *N. ceranae*) or non-simultaneous host–parasite expansions. In contrast, the phylogeography of *O. colligata* is congruent with the host's phylogeography suggesting a long-term association and co-dispersal with the host. Specifically, both host and parasite phylogenies consisted of the same three geographically distinct lineages; the first lineage encompasses Western Eurasian samples, the second East Asian, and the third Northern American samples. Furthermore, in contrast to the previously mentioned microsporidia (*N. ceranae* and *H. tvaerminnensis*), we found a pattern of species-wide IBD in *O. colligata*. IBD has also been described for the host, as well as for a bacterial parasite of *D. magna*, *Pasteuria ramosa* (Andras *et al*. 2018; Fields *et al*. 2018). This pattern further strengthens the assumption of co-dispersal throughout the history of *O. colligata* and *D. magna*.

We have so far not detected *O. colligata* in southern regions. Diagnostic sequencing revealed that parasite samples from the Middle East, which have been identified as *O. colligata* using light microscopy (Goren and Ben-Ami 2013), are likely other species, with very similar morphology and pathology (Fig. 1). However, given the growing evidence that *O. colligata* shows an adaptation to colder habitats, it was not surprising to not find it in southern regions (Kirk *et al*. 2018). In contrast to *O. colligata*, we confirmed the presence of the *D. magna-*specific microsporidia *G. intestinalis* and *M. daphniae* in Southern Europe using microscopy and PCR (Fig. 1). Based on our results, future research should be careful relying on microscopy only for species identification, as we observed several cases of misidentification of microsporidia infecting *D. magna*.

By calibrating the phylogenetic tree of *O. colligata* with the divergence time of the Western Eurasian and East Asian (plus North American) host lineages, a time which has previously been estimated using fossil data (Cornetti *et al*. 2019), we provide the first estimated mutation rate of a microsporidium. Fungi, to which the microsporidia are phylogenetically associated, have been shown to have low mutation rates (e.g. Zhu *et al*. 2014; Ene *et al*. 2018; Bezmenova *et al*. 2020). Our estimate for *O. colligata* falls within the range of previously published mutation rate estimates of fungi. Therefore, we find no evidence that intracellular microsporidia evolve more rapidly than their free-living relatives, which might be expected based on the expectation that parasitism may accelerate the evolutionary process (Moran 1996). However, no other approaches to estimate mutation rates, for example, mutation accumulation experiments, have been performed for microsporidia so far.

### Genome evolution

The evolution of different genome sizes in microsporidia is an ongoing research area of great interest (Parisot *et al*. 2014; Pombert *et al*. 2015; de Albuquerque *et al*. 2020; Angst *et al*. 2022). In microsporidia, genome streamlining is associated with the abandonment of entire metabolic pathways and fewer transposable elements. Furthermore, the maintenance of streamlined microsporidian genomes was possible due to efficient purifying selection, which is only possible with large effective population sizes (Haag *et al*. 2020). In contrast, small effective population sizes, for example, in species that exhibit population bottlenecks during vertical transmission, increase the power of stochastic processes and reduce selection efficacy (Lynch 2007; Haag *et al*. 2020). Also, the proliferation of repetitive elements is less constrained in species with small effective population sizes. Therefore, we wanted to estimate the effective population size and the rate of adaptive substitution, *α* (as a measure for selection efficacy) in the horizontally transmitted *O. colligata* and compare it with another microsporidian parasite of *D. magna*, *H. tvaerminnensis*, which has a mixed-mode transmission and a small effective population size (Angst *et al*. 2022). We would need more diversity by adding more *O. colligata* samples to the dataset or a more closely related outgroup to reliably estimate *α* using MKTs. However, consistent with the expectation of weaker purifying selection in microsporidia of larger genome sizes and additional vertical transmission, we found elevated π_N_/π_S_ ratios in non-BUSCO genes in *H. tvaerminnensis* compared with *O. colligata*. By implication, this means that there is relatively efficient purifying selection in *O. colligata*, likely due to its entirely horizontal transmission and the presumably high *N_e_*. In a previous comparison between the genera *Hamiltosporidium* and *Ordospora*, mixed-mode transmission and larger genome size has been shown to be associated with larger *d_N_*/*d_S_* ratios (Haag *et al*. 2020). We extend the evidential basis with our between-species comparison of population samples.

### Describing genomic variation

We estimated genomic variation of the species-specific parasite *O. colligata* with samples covering a large part of the host's geographic distribution. The use of reference genome-based analyses could bias our estimates of sample differentiation downwards. However, our results and therefore conclusions would not likely be qualitatively different from reference genome-free approaches. The low genomic diversity in *O. colligata* made certain analyses unreliable, i.e. inferring historical *N_e_* and MKTs. Our genomic analyses to determine ploidy levels suggest that *O. colligata* is haploid (we did not conduct cytological analyses). However, two samples were somewhat ambiguous with regard to the haploid pattern (Supplementary Figs. 1 and 2). We speculate that this was in part due to the quantity of segregating polymorphisms and the potential presence of multiple infections of *O. colligata* in the same sample. Our methods to infer the ploidy level of *O. colligata* would fail if *O. colligata* is di- or polyploid with extremely low levels of heterozygosity, an unlikely genomic characteristic.

While low levels of genomic diversity might constrain some population genetic analyses, other important signals might be clearer. For example, because much of the *O. colligata* genome is depauperate in SNP diversity, regions with an increased genetic diversity stand out from the larger genomic context (Fig. 2). Some of these distinctly diverse regions have increased diversity due to host-to-parasite horizontal gene transfer, which Pombert *et al*. (2015) previously described (chromosomes 2, 5, and 8). Specifically, the observed excess diversity may arise as the result of the unintentional mapping of *D. magna* reads to these transferred regions; thus, both the host and parasite variants are conflated. More relevant to the present study is how other outlier regions might be affected differently by evolution. Indeed, some genes with functions relevant to the evolution of microsporidia-specific traits show high sequence variation, e.g. the ribosomal protein L24 (M896_051540), a leucyl-tRNA synthetase (M896_060290), and a putative ABC-like lipid transport protein (M896_121220). The ribosomal protein L24 has been reported by Liu *et al*. (2016) to be differentially expressed before and after the germination of spores of the microsporidium *Nosema bombycis*, suggesting its importance for the regulation of transcriptional and translation activities during the infection process. Like other protein synthetases, Melnikov *et al*. (2018) reported that the leucyl-tRNA synthetase has degenerated in the microsporidium *Vavraia culicis*. Malfunctioning synthetases are hypothesized to produce more diverse proteomes in microsporidia than expected. ABC-like lipid transport proteins are important in parasitic protists, like microsporidia, for their role in nutrient salvage (Dean *et al*. 2014). While most ABC transporters are outward exporters, some are described as importers in parasitic protists. These importers might contribute to the parasite's nutrient uptake when living inside the host cell. Moreover, the putative ABC-like lipid transport protein M896_121220 has the fourth highest π_N_/π_S_ ratio, which is an indicator for positive selection, especially recognizable when compared directly to the genomic background. Among the proteins with high π_N_/π_S_ ratios (Supplementary Table 2), many are annotated as hypothetical proteins. The inability to annotate these genes based on sequence similarity may already be a sign of (1) their fast-evolving nature and (2) their specific function in the context of coevolution between *D. magna* and *O. colligata*.

## Conclusion

The extreme genomic architectures of microsporidia have been known for a while. To understand such large-scale genomic variation, knowledge about the different species’ biology is indispensable. Previously intractable problems in intracellular parasites’ past and present evolutionary dynamics can now be studied using genomic diversity at a fine scale. Furthermore, investigating the evolution of different genome sizes in microsporidia is facilitated by comparing parasites evolving in the same host, where the host factor can be assumed to be similar. Therefore, this study profits from comparisons between two genera of microsporidia, *Hamiltosporidium* and *Ordospora*, which share the host, *D. magna*. The genomes of these microsporidian genera are affected differently by coevolution with the same host. Mainly, we think that the acquisition of vertical transmission in *Hamiltosporidium* led to the expansion of its genome. Contrarily, in the horizontally transmitted *Ordospora*, large effective population size and efficient purifying selection could have helped maintaining its compact genome. This study adds to the growing research of microsporidia supporting the observation that the mode of transmission plays an important role in the evolution of genome size. This hypothesis would benefit from larger comparative studies based on whole-genome data across the clade of the microsporidia.

## Supplementary Material

jkad017_Supplementary_Data

## Data Availability

Analysis scripts are available at https://github.com/pascalangst/Angst_etal_2022_G3, the raw VCF file is deposited at https://doi.org/10.6084/m9.figshare.21701606, and raw data are deposited at the NCBI SRA database (BioProject ID PRJNA814405). Supplemental material available at G3 online.
