# Supplementary material for: Population genetic analysis of the microsporidium Ordospora colligata reveals the role of natural selection and phylogeography on its extremely compact and reduced genome
Source: G3 (Bethesda). 2023 Jan 19;13(3):jkad017. doi: 10.1093/g3journal/jkad017 (PMC9997559; doi:10.1093/g3journal/jkad017)

Supplemental Information for:

Population genetic analysis of the microsporidium *Ordospora colligata* reveals the role of natural selection and phylogeography on its extremely compact and reduced genome

by

Pascal Angst[1*], Dieter Ebert[1], and Peter D. Fields[1]

[1] Department of Environmental Sciences, Zoology, University of Basel, Basel, Switzerland

[*] Corresponding author: pascal.angst@unibas.ch

Supplementary methods

PCR of small subunit of microsporidian ribosomal DNA and Sanger sequencing

We treated one animal per *D. magna* clone with three antibiotics (streptomycin, tetracycline, ampicillin) at a concentration of 50 mg/L each for 24 h to reduce non-focal DNA. To aid gut evacuation, we fed animals after 0 h and 12 h with dextran beads (Sephadex 'Small' by Sigma Aldrich: 50 mm diameter) at a concentration of 0.5 g/100 mL (Dukić et al., 2016). Afterward, we moved individuals into separate wells of an unskirted 96-well PCR plate (Eppendorf) and removed excess fluid with a sterile pipette. Subsequently, we crushed individuals using a customized rack of metallic pestles. To each well, we added 20 µL microLYSIS-Plus DNA release buffer (Microzone, West Sussex, UK) and used the manufacturer's tough cell lysis protocol (65 °C for 15 min; 96 °C for 2 min; 65 °C for 4 min; 96 °C for 1 min; 65 °C for 1 min; 96 °C for 30 s) for incubation in an Eppendorf Mastercycler Nexus. Following this DNA extraction, we diluted the products to 0.1 × with deionized water and set up the PCR reactions. Each 50 µL PCR took place in an Eppendorf Mastercycler Nexus, with each reaction consisting of 29.5 µL deionized water, 10 µL of the diluted sample, 5 µL 10 × PCR buffer, 2 µL forward and reverse primer, 1 µL dNTPs and 0.5 µL Taq DNA polymerase. We used universal primers targeting the small subunit of microsporidian ribosomal DNA (V1f: 5'-CACCAGGTTGATTCTGCCTGAC-3'; 1342r: 5'-ACGGGCGGTGTGTACAAAGAACAG-3'; Madyarova et al., 2015). The PCR conditions were 95 °C for 15 min, followed by 40 cycles of 95 °C for 30 s, 67.5 °C for 90 s, and 72 °C for 90 s, and then 72 °C for 7 min. PCR products were sequenced by Microsynth (Balgach, Switzerland)

using the Sanger method on ABI 3730/3730xl machines (Applied Biosystems; Thermo Fisher Scientific). We compared the obtained sequence data to the NIH genetic sequence database (GenBank; Benson et al., 2013) with the Basic Local Alignment Search Tool for nucleotides (BLASTN) on the NCBI website (https://blast.ncbi.nlm.nih.gov/). The success of the DNA extraction was verified before submission with an additional PCR using a *D. magna*-specific pair of primers and the same PCR conditions, i.e., positive control on PCR conditions. Samples with a microscopically detected microsporidian infection and a positive *D. magna* control but without a PCR product for the microsporidian universal marker were assigned to infection with *M. daphniae*, another gut microsporidium (Haag et al., 2014) which is phenotypically very similar to *O. colligata*, but for which the primers would not be capable of generating an amplicon because it is a very basal microsporidium.

References

Benson, D. A., Cavanaugh, M., Clark, K., Karsch-Mizrachi, I., Lipman, D. J., Ostell, J., & Sayers, E. W. (2013). GenBank. *Nucleic Acids Research*, *41*(Database issue), D36-42. https://doi.org/10.1093/nar/gks1195

Dukić, M., Berner, D., Roesti, M., Haag, C. R., & Ebert, D. (2016). A high-density genetic map reveals variation in recombination rate across the genome of *Daphnia magna*. *BMC Genetics*, *17*(1), 137. https://doi.org/10.1186/s12863-016-0445-7

Haag, K. L., James, T. Y., Pombert, J.-F., Larsson, R., Schaer, T. M. M., Refardt, D., & Ebert, D. (2014). Evolution of a morphological novelty occurred before genome compaction in

a lineage of extreme parasites. *Proceedings of the National Academy of Sciences*, *111*(43), 15480–15485. https://doi.org/10.1073/pnas.1410442111

Madyarova, E. V., Adelshin, R. V., Dimova, M. D., Axenov-Gribanov, D. V., Lubyaga, Y. A., & Timofeyev, M. A. (2015). Microsporidian Parasites Found in the Hemolymph of Four Baikalian Endemic Amphipods. *PLOS ONE*, *10*(6), e0130311. https://doi.org/10.1371/journal.pone.0130311

Supplementary tables

Table S1. Further microsporidian infections in the collection. Gi = *Glugoides intestinalis*, Hm = *Hamiltosporidium magnivora*, Ht = *Hamiltosporidium tvaerminnensis*, Md = *Mitosporidium daphniae*, Oc = *Ordospora colligata*. In some genomic samples, there were no sequencing reads of the parasites that were previously identified by microscopy. This led to try to identify the species with PCR using a universal marker for microsporidia. When there were spores visible under the microscope but no PCR product, the sample was tentatively assigned to be infected with *Mitosporidium daphniae*, an early diverging microsporidium, for which the microsporidian primer pair does not work.

Clone-label	Microscopy suggested Infection	PCR suggested Infection
BE-OM-1 infected O.c.2	Oc	?
BE-KN2-1 infected G.I.	Gi	Md
BE-WH1-2 infected G.i.	Gi	Gi
CH-H-1 infected G.i.	Gi	Md
CH-Z-1 infected G.i.	Gi	Md
CN-WON-1	Oc	Oc
CN-WON-3	Oc	Oc

CN-XIU-1	Md	Md
CN-YUE-1	Md	Md
CZ-N2-1 infected G.i.2	Gi, Oc	Gi
DE-G1-106 infected G.i.	Gi	Md
DE-K-35-9 infected G.i.	Gi, Oc	Gi
DE-R1-1 infected G.i.	Gi	Gi
DE-R1-1 infected M.d.	Md	Md
DE-S2-1 infected G.i	Gi	Md
DE-S3-3infected G.i	Gi	Md
EG-ELIAS-1 G.i.	Gi	Md
ES-DO1-1 infected O.c.	Oc, Ht	Gi
FI-SK-58-2	Ht	NA
GB-EK1-1	Md	Md
GB-EK1-32 infected G.i.	Gi	Md
IE-DUB-1 infected O.c.	Oc	Gi
IL-BN-2	Hm	NA
IL-EY-17 infected O.c.	Oc	Gi
IL-YERU-16 infected O.c.	Oc	Gi
IR-GG-102	Hm	NA
IT-ISR3-2 infected	Oc	Gi
IT-PER-2 infected O.c.	Oc	Gi
IT-PER-2	Ht	NA
IT-PER-10	Ht	NA
RU-AST2-1	Gi	NA

RU-HA4-2 G.i.	Gi	Md
RU-NE1-34 O.c.	Oc	Oc
US-ConnecticutValley M.d.	Md	Md

Table S2. List of *O. colligata* coding sequence (CDS) alignments with π_N/π_S values. The single copy orthologous protein of *O. pajunii* is denoted in the last column. Per-CDS π and π_N/π_S value were calculated using selectionstats.py. The gene annotation is derived from the original record (NCBI database; Assembly name: ASM80326v; GenBank assembly accession: GCA_000803265.1, Bioproject accession: PRJNA210314)

**Locus_tag**	**π**	**π_N/π_S**	**Ortholog**	**Annotation**
M896_010050	0.018745939	0.85449963	Cg_02C_49	hypothetical protein
M896_010080	0	Inf	Cg_01_4	hypothetical protein
M896_010090	0	Inf	Cg_01_6	hypothetical protein
M896_010100	0.001939479	1.10132974	Cg_01_7	fructose-1%2C6-bisphosphate aldolase
M896_010110	0.001010101	Inf	Cg_01_8	hypothetical protein
M896_010120	0.001341922	Inf	Cg_01_9	dynein light chain
M896_010130	0.001410039	1.67706168	Cg_01_10	hypothetical protein
M896_010140	0.001786183	0.7054238	Cg_01_11	hypothetical protein
M896_010150	0.004151404	0.054145	Cg_01_12	hypothetical protein
M896_010160	0	Inf	Cg_01_13	ribosomal protein L2P
M896_010180	0	Inf	Cg_01_14	U3 small nucleolar ribonucleoprotein
M896_010190	0.004188713	1.25639364	Cg_01_15	hypothetical protein
M896_010200	0.002078435	1.4731405	Cg_01_16	DEAD-like helicase
M896_010210	0.002092488	Inf	Cg_01_17	ribosome biogenesis protein BRX1
M896_010220	0.002316406	0.06993267	Cg_01_18	zuotin-like protein
M896_010230	0.001986044	0.27600555	Cg_01_19	cell differentiation protein Rcd1
M896_010240	0	Inf	Cg_01_20	hypothetical protein
M896_010250	0.000335008	0	Cg_01_21	hypothetical protein

M896_010270	0.001202501	Inf	Cg_01_22	hypothetical protein
M896_010280	0	Inf	Cg_01_23	hypothetical protein
M896_010290	0.000408998	0	Cg_01_24	cleavage and polyadenylation specificity factor
M896_010300	0.004299404	Inf	Cg_01_25	hypothetical protein
M896_010310	0.00428027	2.11268212	Cg_01_26	DNA repair protein Rad4
M896_010320	0	Inf	Cg_01_27	actin
M896_010330	0	Inf	Cg_01_28	forkhead/HNF3 transcription factor
M896_010340	0.00355209	0.10344828	Cg_01_29	ribosomal biogenesis protein
M896_010350	0.003064108	1.47857781	Cg_01_30	hypothetical protein
M896_010360	0	Inf	Cg_01_31	RNA polymerase II transcriptional regulation
M896_010370	0.004803241	0.62137428	Cg_01_32	Fe-S cluster assembly protein IscU
M896_010380	0.002480308	0.4859561	Cg_01_33	hypothetical protein
M896_010390	0.002608242	0	Cg_01_34	hypothetical protein
M896_010400	0.003779634	0.78539249	Cg_01_35	peripheral Golgi membrane protein
M896_010410	0.002244669	Inf	Cg_01_36	putative RNA-binding protein
M896_010420	0.004008715	0.25395567	Cg_01_37	small nuclear ribonucleoprotein
M896_010430	0.006518519	1.09413384	Cg_01_39	hypothetical protein
M896_010440	0.002741324	Inf	Cg_01_40	hypothetical protein
M896_010450	0.001496321	0.15625141	Cg_01_42	hypothetical protein
M896_010460	0.004644516	0.4113428	Cg_01_43	hypothetical protein
M896_010470	0.00331675	2.12546125	Cg_01_44	hypothetical protein
M896_010480	0.00379295	0.10511826	Cg_01_45	hypothetical protein
M896_010490	0.004472397	1.50461636	Cg_01_46	DNA-directed RNA polymerase I
M896_010500	0.003118908	1.07469627	Cg_01_47	hypothetical protein
M896_010510	0.001161946	Inf	Cg_01_48	hypothetical protein
M896_010520	0.005359276	1.41994849	Cg_01_49	serine/threonine protein kinase

M896_010530	0.002658238	0.98038902	Cg_01_51	hypothetical protein
M896_010540	0.002974186	0.12404593	Cg_01_52	phosphoacetylglucosamine mutase
M896_010550	0.001698284	0.23423818	Cg_01_53	subunit of RNA polymerase II transcription factor
M896_010560	0	Inf	Cg_01_54	thioredoxin reductase-like protein
M896_010570	0.002539683	0	Cg_01_55	hypothetical protein
M896_010580	0.004786862	0.1450719	Cg_01_56	eukaryotic translation initiation factor 2
M896_010590	0.002552365	0.68646106	Cg_01_57	hypothetical protein
M896_010600	0.000823045	0	Cg_01_58	thioredoxin
M896_010610	0.001068153	0	Cg_01_59	uridine kinase
M896_010630	0.000979816	0.1410386	Cg_01_60	thymidine kinase
M896_010640	0.001763668	Inf	Cg_01_61	HRD ubiquitin ligase complex protein
M896_010650	0.001654081	Inf	Cg_01_62	hypothetical protein
M896_010670	0.002737928	0.59051736	Cg_01_63	RING Zn-finger domain-containing protein
M896_010680	0.002079912	Inf	Cg_01_64	hypothetical protein
M896_010690	0.001129944	Inf	Cg_01_65	trehalose-6-phosphate synthase
M896_010700	0.001358025	Inf	Cg_01_66	hypothetical protein
M896_010710	0.00048011	Inf	Cg_01_67	hypothetical protein
M896_010720	0.003040034	1.23050006	Cg_01_68	CCCH-type Zn-finger protein
M896_010730	0.004148148	2.18560075	Cg_01_69	putative ribonucleoprotein
M896_010740	0.001377018	Inf	Cg_01_70	hypothetical protein
M896_010750	0.002411714	Inf	Cg_01_71	hypothetical protein
M896_010760	0.003400627	0.24071869	Cg_01_72	trehalose 6-phosphate phosphatase
M896_010770	0	Inf	Cg_01_73	hypothetical protein
M896_010780	0.001706776	Inf	Cg_01_74	hypothetical protein
M896_010790	0.002533103	Inf	Cg_01_75	hypothetical protein

M896_010800	0.002325581	0.33647059	Cg_01_76	hypothetical protein
M896_010810	0.001435544	Inf	Cg_01_77	ribosomal protein S12
M896_010820	0.00242963	0.4886231	Cg_01_78	thioredoxin-like protein
M896_010830	0.006990089	3.15791091	Cg_01_79	ubiquitin conjugating enzyme E2
M896_010840	0.001115573	Inf	Cg_01_80	hypothetical protein
M896_010850	0.002104377	Inf	Cg_01_81	hypothetical protein
M896_010860	0	Inf	Cg_01_82	Tat binding protein 1-interacting protein
M896_010870	0.002093967	0.19454274	Cg_01_83	aldo-keto reductase
M896_010880	0.002204586	Inf	Cg_01_84	hypothetical protein
M896_010890	0.003574074	2.44253578	Cg_01_85	WD domain-containing protein
M896_010900	0.001966623	2.09894662	Cg_01_86	hypothetical protein
M896_010910	0.000793651	0	Cg_01_87	ubiquitin-conjugating enzyme E2
M896_010920	0.003036753	0.17901694	Cg_01_88	hypothetical protein
M896_010930	0.001088629	Inf	Cg_01_89	cell cycle control microtubule-binding protein
M896_010940	0.006901311	0.24484536	Cg_01_90	general negative regulator of transcription
M896_010950	0.001724476	0.13979232	Cg_01_91	hypothetical protein
M896_010960	0.001296017	0	Cg_01_92	Rad25-like DNA repair helicase
M896_010970	0.001285304	0.50242175	Cg_01_94	hypothetical protein
M896_010980	0.001462304	0.10264648	Cg_01_95	tRNA/rRNA cytosine-C5-methylase
M896_010990	0.001721551	2.54682884	Cg_01_96	hypothetical protein
M896_011000	0	Inf	Cg_08A_2	SCF ubiquitin ligase and anaphase-promoting complex protein
M896_011010	0.004910053	1.38436482	Cg_01_97	hypothetical protein
M896_011030	0.001332549	0.67364662	Cg_01_98	hypothetical protein
M896_011040	0.010642827	Inf	Cg_01_99	cytochrome B5
M896_011050	0.000575004	0.7301511	Cg_01_100	hypothetical protein

M896_011060	0.001343747	0.56708075	Cg_01_101	S8 serine protease
M896_011070	0.002226631	Inf	Cg_01_102	hypothetical protein
M896_011080	0.002080732	0.25009754	Cg_01_103	cyclin K-like protein
M896_011090	0.002943232	1.77162301	Cg_01_104	chromosome segregation ATPase
M896_011100	0.001538863	0.80761068	Cg_01_105	hypothetical protein
M896_011110	0.00130593	Inf	Cg_01_106	large subunit of replication factor C
M896_011120	0.001308273	0.44743429	Cg_01_107	hypothetical protein
M896_011130	0.000945626	1.00854857	Cg_01_108	hypothetical protein
M896_011140	0.002041706	0.18702264	Cg_01_110	dynamin
M896_011150	0.001304932	0	Cg_01_111	guanylate kinase
M896_011160	0.001312224	0	Cg_01_112	AAA+ ATPase
M896_011170	0.00130552	0.15074788	Cg_01_113	hypothetical protein
M896_011180	0.001109515	Inf	Cg_01_114	hypothetical protein
M896_011190	0.003703704	0.82164634	Cg_01_115	hypothetical protein
M896_011200	0.002008788	0.30274779	Cg_01_116	hypothetical protein
M896_011210	0.004084967	0	Cg_01_117	nuclear distribution C-like protein
M896_011220	0.00318734	0.30058828	Cg_01_118	WD40 domain-containing protein
M896_011230	0.003268543	0.33030686	Cg_01_119	WD40 domain-containing protein
M896_011240	0	Inf	Cg_01_120	frataxin
M896_011250	0.002877026	0.78961309	Cg_01_121	Rho-associated coiled-coil domain-containing protein
M896_011260	0.003843996	0.55141551	Cg_01_122	hypothetical protein
M896_011270	0.004224537	0.625	Cg_01_123	subunit of transcription initiation factor TFIID
M896_011280	0.00390193	0.68674287	Cg_01_124	hypothetical protein
M896_011290	0	Inf	Cg_01_125	hypothetical protein
M896_011300	0.004995693	0.29479374	Cg_01_126	putative RNA-binding protein

M896_011310	0.001230969	Inf	Cg_01_127	septin
M896_011320	0.008354866	Inf	Cg_01_128	hypothetical protein
M896_011330	0.00285658	3.4852768	Cg_01_129	chitin synthase
M896_011340	0	Inf	Cg_01_130	hypothetical protein
M896_011350	0.00139237	0.26599683	Cg_01_131	putative serine/threonine protein kinase
M896_011360	0	Inf	Cg_01_132	hypothetical protein
M896_011370	0.00239899	0.90475551	Cg_01_133	hypothetical protein
M896_011380	0.001987353	0.96621647	Cg_01_134	putative Cdc48 ATPase
M896_011390	0.001432981	Inf	Cg_01_135	subunit of pre-mRNA cleavage GTPase
M896_011400	0.002373247	Inf	Cg_01_136	tyrosinyl-tRNA synthetase
M896_011410	0.002257572	0.65058808	Cg_01_137	hypothetical protein
M896_011420	0.002754036	Inf	Cg_01_138	hypothetical protein
M896_011430	0.002469136	Inf	Cg_01_140	putative proteasome regulatory complex protein
M896_011440	0.000757576	Inf	Cg_01_141	hypothetical protein
M896_011450	0.00409636	0.43985994	Cg_01_142	putative RIO kinase
M896_011460	0.003276353	1.17750901	Cg_01_143	RNA pol Rpb4 domain-containing protein
M896_011470	0.000877461	Inf	Cg_01_144	hypothetical protein
M896_011480	0.001508916	0.15600364	Cg_01_145	hypothetical protein
M896_011490	0.002572802	0.09987333	Cg_01_146	putative proliferating cell nuclear antigen
M896_011500	0.001394091	0.61201781	Cg_01_147	putative centromere/microtubule binding protein
M896_011510	0.002493108	0.97502812	Cg_01_148	WD G-beta repeat domain-containing protein
M896_011520	0.002122122	0.15831407	Cg_01_149	ADP-ribosylation factor family domain-containing protein
M896_011530	0.002751323	0.18122644	Cg_01_150	type-B DNA-directed DNA polymerase
M896_011540	0.003760684	1.69570557	Cg_01_151	subunit 10 of anaphase-promoting complex

M896_011550	0.002844281	0.08844973	Cg_01_152	hypothetical protein
M896_011560	0.001203704	0.88978826	Cg_01_153	putative SAM dependent methyltransferase
M896_011570	0.001730038	0.11754634	Cg_01_154	putative superfamily II RNA helicase
M896_011580	0.002523047	0.67199931	Cg_01_155	phosphatidylinositol transfer protein
M896_011590	0.004587542	0.38894797	Cg_01_156	ribosomal protein S9
M896_011600	0.002614379	Inf	Cg_01_157	small nuclear ribonucleoprotein
M896_011610	0.005705706	Inf	Cg_01_158	ribosomal protein L21
M896_011620	0.007520786	1.78087997	Cg_01_159	hypothetical protein
M896_011630	0.002402402	Inf	Cg_01_160	SecE/Sec61-gamma subunit of protein translocation complex
M896_011650	0.004746788	0.3402747	Cg_01_161	hypothetical protein
M896_011660	0.000705467	Inf	Cg_01_162	HAM1 domain-containing protein
M896_011670	0.002558635	0.57333016	Cg_01_163	6-phosphogluconate dehydrogenase
M896_011680	0.003703704	0.1283164	Cg_01_164	hypothetical protein
M896_011690	0.001458151	Inf	Cg_01_165	PX domain-containing protein
M896_011700	0.003802733	0.246482	Cg_01_166	hypothetical protein
M896_011710	0.000750117	Inf	Cg_01_167	hypothetical protein
M896_011720	0.0018107	0.08248233	Cg_01_168	serine/threonine protein phosphatase 2A-like protein
M896_011730	0.002539683	0.16925422	Cg_01_169	putative GTPase
M896_011740	0.001711672	0.04708031	Cg_01_171	hypothetical protein
M896_011750	0.001730703	0	Cg_01_172	hypothetical protein
M896_011760	0.002651515	0.10824321	Cg_01_173	minichromosome maintenance protein
M896_011770	0.00331384	0.08689655	Cg_01_174	hypothetical protein
M896_011780	0.000746714	0	Cg_01_175	peptidase C48 domain-containing protein
M896_011790	0.007726692	0.90826483	Cg_01_176	hypothetical protein

M896_011800	0.003977611	0.12328915	Cg_01_177	hypothetical protein
M896_011810	0.001229314	0	Cg_01_178	serine palmitoyltransferase
M896_011820	0.003534392	0.1894303	Cg_01_179	hypothetical protein
M896_011830	0.00210887	0.86473191	Cg_01_180	RhoGAP domain-containing protein
M896_011840	0.001858788	0.10797392	Cg_01_181	hypothetical protein
M896_011850	0.001361807	0.25876578	Cg_01_182	putative membrane protein
M896_011860	0.004004711	Inf	Cg_01_183	putative pseudouridine synthase
M896_011870	0.001561365	0.18042913	Cg_01_184	ribosomal protein S6e
M896_011880	0.000877655	Inf	Cg_01_185	hypothetical protein
M896_011890	0.002011696	Inf	Cg_01_186	glucose-6-phosphate isomerase
M896_011900	0.004277517	Inf	Cg_01_187	hypothetical protein
M896_011910	0.003441595	3.43073342	Cg_01_188	putative serine/threonine kinase
M896_011920	0	Inf	Cg_01_189	Ran GTPase-activating protein
M896_011930	0.004523608	0.71771351	Cg_01_190	hypothetical protein
M896_011940	0.000335008	Inf	Cg_01_191	putative ribosomal protein L1
M896_011960	0	Inf	Cg_01_192	hypothetical protein
M896_011970	0.003472222	0.70340796	Cg_01_193	putative amino acid transporter
M896_011980	0.003892028	0.1875635	Cg_01_194	hypothetical protein
M896_011990	0.001082954	0.44444444	Cg_01_195	Fcf1 domain-containing protein
M896_012000	0.003844901	0.53945753	Cg_01_196	hypothetical protein
M896_012010	0.003000686	0.36648091	Cg_01_197	FAT domain-containing protein
M896_012020	0.00617284	Inf	Cg_01_198	hypothetical protein
M896_012030	0.003779912	0.17087064	Cg_01_199	hypothetical protein
M896_012040	0.003753086	0.14084459	Cg_01_200	hypothetical protein
M896_012050	0.000642479	0	Cg_01_201	putative transcription initiation factor TFIIIB
M896_012060	0.002153316	Inf	Cg_01_202	hypothetical protein

M896_012070	0.00280713	1.59236889	Cg_01_203	hypothetical protein
M896_012080	0.003724166	0.76611437	Cg_01_204	ribosomal protein L10-like protein
M896_012090	0.007215007	0.48609865	Cg_01_205	hypothetical protein
M896_012100	0.002604803	Inf	Cg_01_206	putative phosphoesterase
M896_012110	0.001489533	Inf	Cg_01_207	subunit C of CCAAT-binding factor
M896_012120	0.002621929	1.16381788	Cg_01_208	calcineurin-like phosphoesterase
M896_012130	0.004675398	0.68594541	Cg_01_209	hypothetical protein
M896_012140	0.001932367	Inf	Cg_01_210	hypothetical protein
M896_012150	0.001046244	0.55310522	Cg_01_211	hypothetical protein
M896_012160	0.003903305	Inf	Cg_01_212	putative GTPase
M896_012170	0.003130511	Inf	Cg_01_213	putative subunit E of vacuolar ATP synthase
M896_012180	0.003246274	0.84061396	Cg_01_214	hypothetical protein
M896_012190	0.00201788	0.40929513	Cg_01_215	beta-CASP domain-containing protein
M896_012200	0.003828548	0.07998347	Cg_01_216	hypothetical protein
M896_012210	0.002344116	Inf	Cg_01_217	hypothetical protein
M896_012220	0.002830992	0.45363525	Cg_01_218	eukaryotic peptide chain release factor eRF1
M896_012230	0.002292769	0.24328445	Cg_01_219	subunit RSC8 of RSC chromatin remodeling complex
M896_012240	0.004004004	0.22561649	Cg_01_220	hypothetical protein
M896_012250	0.00653046	0.33631234	Cg_01_221	DNA polymerase alpha/epsilon-like protein
M896_012260	0.002866763	0.55277567	Cg_01_222	phosphoglycerate kinase
M896_012270	0.002262713	0.12306085	Cg_01_223	acetyl-CoA synthetase
M896_012280	0.002275225	0.54023621	Cg_01_224	putative DNA mismatch repair protein
M896_012290	0.00456621	1.48472849	Cg_01_225	beta type-4 subunit of proteasome
M896_012300	0	Inf	Cg_01_226	hypothetical protein
M896_012310	0.005372405	0.57260008	Cg_01_227	subunit Rpb11 of RNA polymerase II

M896_012320	0.002159594	0.16097527	Cg_01_228	glycerol 3 phosphate dehydrogenase
M896_012330	0.001333333	0.21150662	Cg_01_229	phosphomannomutase
M896_012340	0.000510856	0	Cg_01_230	ribosomal protein S3AE
M896_012350	0.002664324	0.44835523	Cg_01_231	putative nitric-oxide synthase
M896_012360	0.004719764	0.03512364	Cg_01_232	hypothetical protein
M896_012370	0.002554278	0.38788231	Cg_01_233	WD40 domain-containing protein
M896_012380	0.003483682	0.54597084	Cg_01_235	hypothetical protein
M896_012390	0.004553734	Inf	Cg_01_236	hypothetical protein
M896_012400	0.00071817	0.11654908	Cg_01_237	hypothetical protein
M896_012410	0.003367003	Inf	Cg_01_238	protein phosphatase inhibitor
M896_012420	0.003037687	0.62382626	Cg_01_240	hypothetical protein
M896_012430	0.002880658	0.62722689	Cg_01_241	hypothetical protein
M896_012440	0.002301994	0.74834356	Cg_01_242	hypothetical protein
M896_012450	0.00203828	0.08524519	Cg_01_243	hypothetical protein
M896_012460	0.002270884	2.17280164	Cg_01_244	hypothetical protein
M896_012470	0.001095462	0	Cg_01_245	hypothetical protein
M896_012480	0.001563031	0.31595031	Cg_01_246	putative Sar1 GTPase
M896_012500	0.00222475	0.72859528	Cg_01_247	hypothetical protein
M896_012510	0.001884253	1.90905218	Cg_01_248	kinesin motor domain-containing protein
M896_012520	0.007267645	0.85092913	Cg_01_250	hypothetical protein
M896_020020	0.001302932	Inf	Cg_02A_1	hypothetical protein
M896_020030	0.001725178	0.98563066	Cg_02A_2	hypothetical protein
M896_020040	0.000938967	Inf	Cg_02A_3	hypothetical protein
M896_020050	0.000814625	Inf	Cg_02A_4	aminotransferase
M896_020060	0.002259887	1.29421719	Cg_02A_5	thioredoxin domain-containing protein
M896_020070	0.000857055	Inf	Cg_02A_6	hypothetical protein

M896_020080	0.00316358	Inf	Cg_02A_7	hypothetical protein
M896_020090	0.003420167	1.9242766	Cg_02A_8	hypothetical protein
M896_020110	0.001058201	Inf	Cg_02B_1	ribosomal protein L6
M896_020120	0.003524229	0.06099234	Cg_02B_2	beta subunit of transcription initiation factor IIF
M896_020130	0.002987862	0	Cg_02B_3	ribosomal protein S17
M896_020140	0.002962963	0	Cg_02B_4	S26 type I signal peptidase
M896_020150	0.005163818	0.69874052	Cg_02B_5	ribosomal protein L7a
M896_020160	0	Inf	Cg_02B_6	ubiquitin
M896_020170	0.001364522	0.08122931	Cg_02B_8	poly(A) polymerase
M896_020180	0.001364522	Inf	Cg_02B_9	putative histone-like transcription factor
M896_020190	0.000846922	Inf	Cg_02B_10	hypothetical protein
M896_020210	0.000914495	Inf	Cg_02B_11	hypothetical protein
M896_020220	0.001119105	0	Cg_02B_12	HscB-like chaperone
M896_020230	0.002515202	0.32415793	Cg_02B_13	hypothetical protein
M896_020240	0.002769864	0.79524564	Cg_02B_14	dead box helicase
M896_020250	0.00130527	0.37249795	Cg_02B_15	hypothetical protein
M896_020260	0.001062733	Inf	Cg_02B_16	hypothetical protein
M896_020270	0.001022431	Inf	Cg_02B_17	hypothetical protein
M896_020280	0.001431493	0.69494389	Cg_02B_18	hypothetical protein
M896_020290	0.001929012	Inf	Cg_02B_19	hypothetical protein
M896_020300	0	Inf	Cg_02B_20	ribosomal protein L5
M896_020310	0.002923977	0.13333333	Cg_02B_21	hypothetical protein
M896_020320	0.001952695	0.31286979	Cg_02B_22	hypothetical protein
M896_020330	0.002746567	0.31752447	Cg_02B_23	hydroxyacylglutathione hydrolase
M896_020340	0.003311735	2.42754934	Cg_02B_24	hypothetical protein

M896_020350	0.002498282	0.44778049	Cg_02B_26	hypothetical protein
M896_020360	0.001757444	0.2524731	Cg_02B_27	catalytic protein kinase
M896_020370	0.00138001	Inf	Cg_02B_28	hypothetical protein
M896_020390	0.005072464	0.43454385	Cg_02B_29	hypothetical protein
M896_020410	0.00134959	0	Cg_02B_30	subunit delta of TCP-1 chaperonin
M896_020420	0.003003003	0.20060904	Cg_02B_31	mitogen-activated protein kinase
M896_020430	0.000758808	0	Cg_02B_32	hypothetical protein
M896_020440	0.001661475	Inf	Cg_02B_33	hypothetical protein
M896_020450	0.002283557	0.03217879	Cg_02B_34	26S proteasome regulatory complex protein
M896_020460	0.001218535	0.16074627	Cg_02B_35	hypothetical protein
M896_020470	0.001223242	1.19732137	Cg_02B_36	nucleoporin autopeptidase domain-containing protein
M896_020480	0.002079272	0	Cg_02B_37	gamma subunit of transcription initiation factor IIA
M896_020490	0.001023123	0.4095334	Cg_02B_38	glutathione peroxidase
M896_020500	0.001219326	0.09015589	Cg_02B_39	Sec1-like intracellular trafficking protein
M896_020510	0	Inf	Cg_02B_41	hypothetical protein
M896_020520	0.001460616	0	Cg_02B_42	Ras-like GTP binding protein
M896_020530	0.001040129	0.06894734	Cg_02B_43	hypothetical protein
M896_020540	0.000794786	Inf	Cg_02B_44	hypothetical protein
M896_020550	0.002425201	0.09078422	Cg_02B_45	hypothetical protein
M896_020560	0.00225802	0.27966594	Cg_02B_47	hypothetical protein
M896_020570	0.001271979	0.80658138	Cg_02B_48	beta type-3 subunit of proteasome
M896_020580	0.001560758	2.61579864	Cg_02B_49	putative E1-E2 ATPase
M896_020590	0.000882585	Inf	Cg_02B_50	hypothetical protein
M896_020610	0.004542278	1.11639377	Cg_02B_51	hypothetical protein

M896_020620	0	Inf	Cg_02B_52	small subunit of replication factor C
M896_020630	0.002383253	0.36582654	Cg_02B_53	hypothetical protein
M896_020640	0.001252885	0.18056655	Cg_02B_54	hypothetical protein
M896_020650	0.001143425	0.46940559	Cg_02B_55	GATA zinc finger domain-containing protein
M896_020660	0.003794778	0.41067285	Cg_02B_56	putative RNA-binding protein
M896_020670	0.002904145	0.10488197	Cg_02B_57	Sof1 domain-containing U3 snoRNP protein
M896_020680	0.000524934	0	Cg_02B_58	hypothetical protein
M896_020690	0.001362282	0	Cg_02B_59	hypothetical protein
M896_020700	0.002331265	Inf	Cg_02B_60	hypothetical protein
M896_020710	0.00114899	0.03937497	Cg_02B_61	Smp2-like plasmid maintenance protein
M896_020730	0.006466466	0.28326446	Cg_02B_63	hypothetical protein
M896_020740	0	Inf	Cg_02B_65	hypothetical protein
M896_020750	0.001410935	Inf	Cg_02B_66	hypothetical protein
M896_020760	0.000861326	Inf	Cg_02B_67	putative Ran GTPase binding protein
M896_020770	0.004276441	Inf	Cg_02B_68	hypothetical protein
M896_020780	0.002717003	0.87216806	Cg_02B_69	hypothetical protein
M896_020790	0.004454626	0.32999905	Cg_02B_70	hypothetical protein
M896_020800	0.001619626	0.35015252	Cg_02B_71	Hsp70-like protein
M896_020840	0.003240741	0.6440703	Cg_02C_54	ribosomal protein S8
M896_020860	0.000653595	Inf	Cg_02C_56	ribosomal protein L35Ae
M896_020870	0.00505698	Inf	Cg_02C_57	hypothetical protein
M896_020880	0.002948222	0.59245018	Cg_02C_58	hypothetical protein
M896_020890	0.001328751	0.10105195	Cg_02C_59	Suf domain-containing protein
M896_020900	0.001016703	Inf	Cg_02C_60	thioredoxin reductase
M896_020910	0	Inf	Cg_02C_61	hypothetical protein
M896_020920	0.00136876	Inf	Cg_02C_62	Emp24/gp25L domain-containing protein

M896_020930	0.0004329	0	Cg_02C_63	transport protein particle complex protein
M896_020940	0.004884595	Inf	Cg_02C_64	hypothetical protein
M896_020950	0.002642844	0.46128492	Cg_02C_65	hypothetical protein
M896_020960	0.001642772	1.34502924	Cg_02C_66	hypothetical protein
M896_020970	0.003544214	0.29260116	Cg_02C_67	hypothetical protein
M896_020980	0.004004004	Inf	Cg_02C_68	LSM domain-containing protein
M896_020990	0.000650329	0.39879121	Cg_02C_69	hypothetical protein
M896_021000	0.002204586	Inf	Cg_02C_70	hypothetical protein
M896_021020	0	Inf	Cg_02C_71	putative nascent polypeptide-associated complex protein
M896_021030	0.001676677	Inf	Cg_02C_72	subunit alpha of protein prenyltransferase
M896_021040	0.002382118	0.86590853	Cg_02C_73	hypothetical protein
M896_021050	0	Inf	Cg_02C_74	ubiquitin domain-containing protein
M896_021060	0.002210275	0.94133632	Cg_02C_75	Rad3-like DNA-binding helicase
M896_021070	0.001436008	Inf	Cg_02C_76	heat shock protein 90
M896_021080	0.00303757	0.91521659	Cg_02C_77	hypothetical protein
M896_021090	0.001960077	0.18870268	Cg_02C_78	protein kinase domain-containing protein
M896_021100	0.001143118	Inf	Cg_02C_79	microsomal signal peptidase
M896_021110	0.001380328	0.09116657	Cg_02C_80	DNA replication licensing factor
M896_021120	0.000935279	Inf	Cg_02C_81	hypothetical protein
M896_021130	0.002763385	Inf	Cg_02C_82	nuclear transport factor 2
M896_021140	0.002429943	0.52634722	Cg_02C_83	hypothetical protein
M896_021150	0.00337132	0.14209539	Cg_02C_84	hypothetical protein
M896_021160	0.012913799	1.78067583	Cg_02C_85	homeobox domain-containing protein
M896_021170	0.001339906	0.40966864	Cg_02C_86	tRNA synthetase class I
M896_021180	0.002380952	Inf	Cg_02C_87	DNA ligase

M896_021190	0.001642315	2.20377907	Cg_02C_88	Sec23/Sec24-like protein
M896_021200	0.002121583	0.90472382	Cg_02C_89	chromosome condensation complex Condensin
M896_021210	0.004269547	0.34653795	Cg_02C_90	transcription initiation factor TFIID
M896_021220	0.0004004	Inf	Cg_02C_91	hypothetical protein
M896_021230	0.001169591	Inf	Cg_02C_92	mitosis protein Dim1
M896_021240	0.005934343	0.40806975	Cg_02C_93	hypothetical protein
M896_021250	0.0018107	0.75900621	Cg_02C_94	hypothetical protein
M896_021260	0.001959379	4.43704438	Cg_02C_95	dolichyl-phosphate-mannose-protein
M896_021270	0.001583468	0.31862857	Cg_02C_96	hypothetical protein
M896_021280	0.001424501	2.55077479	Cg_02C_97	hypothetical protein
M896_021290	0.001725998	Inf	Cg_02C_98	hypothetical protein
M896_021300	0.001146601	1.54505819	Cg_02C_99	hypothetical protein
M896_021310	0	Inf	Cg_02C_100	transcription factor E2F
M896_021320	0.001827802	Inf	Cg_02C_101	prolyl-tRNA synthetase
M896_021330	0.002136526	0.66665362	Cg_02C_102	trehalase
M896_021340	0.003024213	0.55431973	Cg_02C_103	peptidase M48 domain-containing protein
M896_021350	0.005334692	0.27218973	Cg_02C_104	hypothetical protein
M896_021360	0	Inf	Cg_02C_105	hypothetical protein
M896_021370	0.003550182	0.76717358	Cg_02C_106	hypothetical protein
M896_021380	0.002015504	0.16683854	Cg_02C_107	subunit A of RAB protein geranylgeranyltransferase
M896_021390	0.001851852	Inf	Cg_02C_108	hypothetical protein
M896_021400	0.000474074	Inf	Cg_02C_109	hypothetical protein
M896_021410	0.002162162	0.22339473	Cg_02C_110	hypothetical protein
M896_021420	0.004302652	0.29710459	Cg_02C_111	serine/threonine kinase

M896_021430	0.001957071	0.47691321	Cg_02C_112	Sec23 domain-containing protein
M896_021440	0.003433897	0	Cg_02C_113	prenylated RAB acceptor 1
M896_021450	0.002616747	0.4118451	Cg_02C_114	hypothetical protein
M896_021470	0.002296469	0.13573342	Cg_02C_115	alanyl-tRNA synthetase
M896_021480	0.003809524	0.31643681	Cg_02C_117	subunit A of transcription initiation factor TFIID
M896_021490	0.0039675	0.24585153	Cg_02C_118	hypothetical protein
M896_021500	0.002175476	0.1073574	Cg_02C_119	putative DEAD-box helicase
M896_021510	0.003708972	0.41818705	Cg_02C_120	hypothetical protein
M896_021520	0.00043573	Inf	Cg_02C_121	putative Per1-like membrane protein
M896_021550	0.001270879	0	Cg_02C_124	ATP-dependent RNA helicase
M896_021560	0.002113776	0.07677599	Cg_02C_125	glycosyltransferase
M896_021570	0.001351717	Inf	Cg_02C_126	Ca2+-binding protein
M896_021580	0.002063492	Inf	Cg_02C_127	hypothetical protein
M896_021590	0.001169591	Inf	Cg_02C_128	RNA-binding protein
M896_021600	0.002176584	0.1684744	Cg_02C_129	ribosomal protein S3
M896_021610	0.001294498	1.24410616	Cg_02C_130	RIO protein kinase
M896_021620	0.001975309	0.68408473	Cg_02C_131	hypothetical protein
M896_021630	0.004601571	Inf	Cg_02C_132	ribosomal protein S28
M896_021640	0.004383081	1.37181773	Cg_02C_133	hypothetical protein
M896_021650	0.002926383	1.41867846	Cg_02C_134	hypothetical protein
M896_021660	0.006070732	0.21252856	Cg_02C_135	DNA repair protein Mms21
M896_021670	0.002460222	0.10195448	Cg_02C_136	histone acetyltransferase
M896_021680	0.003292181	0.84983515	Cg_02C_137	endochitinase
M896_021690	0.002617458	Inf	Cg_02C_138	C-terminal domain of replication factor C
M896_021700	0.001786183	0	Cg_02C_139	hypothetical protein

M896_021710	0.001998824	Inf	Cg_02C_140	ribosomal protein S15A
M896_021720	0.001102984	0.71422951	Cg_02C_141	minichromosome maintenance protein
M896_021730	0.002502503	Inf	Cg_02C_142	hypothetical protein
M896_021740	0.004032922	Inf	Cg_02C_143	glutaredoxin
M896_021750	0.000861326	0.29261759	Cg_02C_144	ribosome stability and mRNA decay protein
M896_021760	0.001815182	Inf	Cg_02C_145	DNA helicase TIP49
M896_021780	0.000513067	Inf	Cg_02C_146	hypothetical protein
M896_021790	0.004487475	1.52558852	Cg_02C_147	hypothetical protein
M896_021800	0.002324037	1.48912188	Cg_02C_148	P-type ATPase
M896_021810	0.002394314	Inf	Cg_02C_149	Rab GTPase
M896_021820	0.001026495	Inf	Cg_02C_150	hypothetical protein
M896_021830	0	Inf	Cg_02C_151	hypothetical protein
M896_021840	0.000255428	Inf	Cg_02C_153	hypothetical protein
M896_021850	0	Inf	Cg_02C_154	hypothetical protein
M896_021860	0	Inf	Cg_02C_155	hypothetical protein
M896_021870	0.002203857	Inf	Cg_02C_156	hypothetical protein
M896_021880	0.000798403	0.25354462	Cg_02C_157	hypothetical protein
M896_030080	0.0043409	0.68284812	Cg_03_105	chromatin organization modifier protein
M896_030090	0.002469136	Inf	Cg_03_106	hypothetical protein
M896_030100	0.002893519	Inf	Cg_03_107	hypothetical protein
M896_030110	0.001373737	Inf	Cg_03_108	nucleotide sugar transporter
M896_030120	0.005196241	Inf	Cg_03_109	hypothetical protein
M896_030130	0.001722653	Inf	Cg_03_110	putative transcription initiation factor IIF auxiliary protein
M896_030140	0.002741467	Inf	Cg_03_111	ankyrin-like protein
M896_030150	0.010441292	0.42946165	Cg_03_112	hypothetical protein

M896_030160	0.001080247	Inf	Cg_03_113	Ca2+-binding protein
M896_030170	0.002153316	Inf	Cg_03_114	Rho-like GTPase
M896_030180	0.003122731	Inf	Cg_03_115	hypothetical protein
M896_030190	0.00292583	1.97664328	Cg_03_116	Snf2-like DNA/RNA helicase
M896_030200	0.003233912	Inf	Cg_03_117	WD40 domain-containing protein
M896_030210	0	Inf	Cg_03_118	subunit B of CCAAT-binding transcription factor
M896_030220	0.000904722	Inf	Cg_03_119	hypothetical protein
M896_030230	0.004320988	0.48911652	Cg_03_120	hypothetical protein
M896_030240	0.002889703	0.84044028	Cg_03_121	ribosomal protein L18
M896_030250	0.003186908	Inf	Cg_03_122	hypothetical protein
M896_030260	0.003328645	0.44813293	Cg_03_123	hypothetical protein
M896_030270	0.00047619	Inf	Cg_03_124	histone H3
M896_030280	0.001731948	Inf	Cg_03_125	hypothetical protein
M896_030290	0.001614885	0.679398	Cg_03_126	tRNA nucleotidyltransferase/poly(A) polymerase
M896_030300	0.002939447	Inf	Cg_03_127	Rab GTPase
M896_030310	0.002341201	1.5542255	Cg_03_128	hypothetical protein
M896_030320	0.001562692	Inf	Cg_03_129	Cdc48-like ATPase
M896_030330	0.000296956	0.2529844	Cg_03_130	hypothetical protein
M896_030350	0.000675858	Inf	Cg_03_131	putative sugar kinase
M896_030360	0.000993413	0.29918737	Cg_03_132	PelA-like RNA-binding protein
M896_030370	0.000534188	0.61572652	Cg_03_133	histone deacetylase
M896_030380	0.001839588	0.63313028	Cg_03_134	Myg1-like protein
M896_030390	0.001570101	3.91324622	Cg_03_135	hypothetical protein
M896_030400	0.00220817	Inf	Cg_03_136	regulatory subunit of ATP-dependent 26S proteasome

M896_030420	0.003025561	0.56817533	Cg_03_137	hypothetical protein
M896_030430	0.003139856	Inf	Cg_03_138	molybdopterin biosynthesis protein MoeB
M896_030440	0.00139693	Inf	Cg_03_139	hypothetical protein
M896_030450	0.001786711	Inf	Cg_03_140	cytidylate kinase
M896_030460	0	Inf	Cg_03_141	hypothetical protein
M896_030470	0.000646465	Inf	Cg_03_142	hypothetical protein
M896_030480	0.00229455	Inf	Cg_03_143	adenine nucleotide alpha hydrolase
M896_030490	0.000613196	Inf	Cg_03_144	hypothetical protein
M896_030510	0.00326147	0.3248074	Cg_03_1	hypothetical protein
M896_030520	0.002527233	0	Cg_03_2	peroxiredoxin
M896_030530	0.004098584	0.32321933	Cg_03_3	peptidase S8-like protein
M896_030550	0.003132648	0.06923077	Cg_03_4	homeodomain-containing transcription factor
M896_030560	0.001033592	0.15866655	Cg_03_5	Sac-like phosphoinositide polyphosphatase
M896_030570	0.002040364	Inf	Cg_03_6	calponin
M896_030580	0.002595758	Inf	Cg_03_7	hypothetical protein
M896_030590	0.001760176	0.54443667	Cg_03_8	DNA repair Rad51-like protein
M896_030600	0.002308802	0.16	Cg_03_9	putative subunit of V-type ATP synthase
M896_030610	0	Inf	Cg_03_10	hypothetical protein
M896_030620	0.003271077	0.54530664	Cg_03_11	N-myristoyl transferase
M896_030630	0.002645503	0.27556185	Cg_03_12	hypothetical protein
M896_030640	0.003462812	0.21345807	Cg_03_13	phosphatidylinositol 4-kinase
M896_030660	0.001556811	0.14099574	Cg_03_14	hypothetical protein
M896_030670	0.001266774	0.19944006	Cg_03_15	flap endonuclease 1
M896_030680	0	Inf	Cg_03_16	large chain of transcription initiation factor IIA
M896_030690	0.001101101	0.11605387	Cg_03_18	hypothetical protein
M896_030700	0.000659134	0	Cg_03_19	hypothetical protein

M896_030710	0	Inf	Cg_03_20	putative subunit of transcription initiation factor TFIIIB
M896_030720	0.001082954	0.24918831	Cg_03_21	Cdc6-like protein
M896_030730	0.000920447	Inf	Cg_03_22	hypothetical protein
M896_030740	0.001249092	0.19212188	Cg_03_23	14-3-3-like signal transduction protein
M896_030750	0.000774107	0	Cg_03_24	hypothetical protein
M896_030760	0.001916681	2.46837349	Cg_03_25	SCP/PR1 domain-containing protein
M896_030770	0.002642713	0.06383632	Cg_03_26	serine/threonine protein kinase
M896_030780	0.003606663	1.01401049	Cg_03_27	hypothetical protein
M896_030790	0.001611653	0.18689785	Cg_03_28	hypothetical protein
M896_030800	0.002303143	0	Cg_03_29	ribosomal protein L7
M896_030810	0.001768933	0.25764739	Cg_03_30	putative G2/M transition transcriptional factor
M896_030820	0.002354354	2.81597527	Cg_03_31	ubiquitin-activating enzyme E1
M896_030830	0.002600473	0.08611919	Cg_03_32	putative tRNA-intron endonuclease
M896_030840	0.004038066	0.49900784	Cg_03_33	protein kinase
M896_030850	0.000995237	0.04071951	Cg_03_34	Sec1-like intracellular trafficking protein
M896_030860	0.002264511	0.35213329	Cg_03_35	haspin serine/threonine kinase
M896_030870	0.000893092	0.7331784	Cg_03_36	hypothetical protein
M896_030880	0.003220612	0.27110838	Cg_03_37	hypothetical protein
M896_030890	0.001638807	0	Cg_03_39	hypothetical protein
M896_030900	0.002126075	0.49933264	Cg_03_40	hypothetical protein
M896_030910	0.002962963	0.76388889	Cg_03_41	small nuclear ribonucleoprotein
M896_030920	0.002541757	0.59049959	Cg_03_42	hypothetical protein
M896_030930	0.0026046	Inf	Cg_03_43	beta-tubulin
M896_030940	0.004861111	0.18064601	Cg_03_44	putative sedlin
M896_030950	0.001247563	Inf	Cg_03_45	ubiquitin-related modifier-like protein

M896_030960	0.003449528	0.66042003	Cg_03_46	hypothetical protein
M896_030970	0.003227881	0.19663889	Cg_03_47	hypothetical protein
M896_030980	0.003327056	0.30386323	Cg_03_48	hypothetical protein
M896_030990	0	Inf	Cg_03_49	RNA-binding protein
M896_031000	0.002164502	Inf	Cg_03_50	RNA-binding protein
M896_031010	0.002180194	2.25525933	Cg_03_51	RNA binding domain-containing protein
M896_031020	0.001139601	Inf	Cg_03_53	ribosomal protein L34
M896_031030	0.002220257	5.91549879	Cg_03_54	exoribonuclease R
M896_031050	0.001840234	2.0231777	Cg_03_55	ATP-dependent 6-phosphofructokinase
M896_031060	0.000819403	Inf	Cg_03_56	ribonuclease HII
M896_031070	0.001722995	0.516649	Cg_03_57	isopeptidase T
M896_031080	0.001481481	0.13137336	Cg_03_58	ribosomal protein S14
M896_031090	0.001668335	0.12784091	Cg_03_59	hypothetical protein
M896_031100	0.000793651	0.20480405	Cg_03_60	protein kinase
M896_031110	0.001867414	Inf	Cg_03_61	homeodomain-containing transcription factor
M896_031120	0.001028807	Inf	Cg_03_62	Rab GTPase-interacting Golgi membrane protein
M896_031140	0.002580108	0.79170647	Cg_03_64	vacuolar import and degradation protein
M896_031150	0.001618837	Inf	Cg_03_65	putative GTPase-activating protein
M896_031160	0.00256021	3.87616822	Cg_03_66	Rho/Rac/Cdc42-like GTPase guanine nucleotide exchange factor
M896_031170	0.001456513	0.63482892	Cg_03_67	MutS-like mismatch repair ATPase
M896_031180	0.001765719	0	Cg_03_68	synaptobrevin/VAMP-like protein
M896_031190	0.001411456	0	Cg_03_69	heat shock protein 70
M896_031200	0.002316081	1.78826605	Cg_03_70	hypothetical protein
M896_031210	0.002341551	0.98767814	Cg_03_71	subunit C of vacuolar H+-ATPase V1

M896_031220	0	Inf	Cg_03_72	subunit M of DNA-directed RNA polymerase
M896_031230	0.003959935	1.07060145	Cg_03_73	subunit 2 of splicing factor 3a
M896_031240	0.001108893	Inf	Cg_03_74	ADP-ribosylation factor-like GTPase
M896_031250	0.000955043	Inf	Cg_03_75	sirtuin-like protein
M896_031260	0.002350427	Inf	Cg_03_76	hypothetical protein
M896_031270	0.000329048	Inf	Cg_03_77	subunit B of DNA-directed RNA polymerase
M896_031280	0.001388889	0.58769561	Cg_03_78	WD40 domain-containing protein
M896_031290	0.00169714	Inf	Cg_03_79	Spt16/Cdc68-like protein
M896_031300	0.001843158	0.40128792	Cg_03_80	hypothetical protein
M896_031310	0.000880301	0.88459888	Cg_03_81	hypothetical protein
M896_031320	0.00119431	2.36063346	Cg_03_82	ABCG-like transporter
M896_031330	0.006530214	0.48815094	Cg_03_83	hypothetical protein
M896_031340	0.000854701	0	Cg_03_84	hypothetical protein
M896_031350	0.001456157	0	Cg_03_85	hypothetical protein
M896_031360	0.004906205	1.41571061	Cg_03_86	U6 snRNA associated Sm-like protein
M896_031380	0	Inf	Cg_03_87	hypothetical protein
M896_031390	0.005206656	1.69680902	Cg_03_88	phosphatidylinositol-4-phosphate 5-kinase
M896_031410	0.001667893	0.74696545	Cg_03_89	ribosomal protein L13
M896_031420	0.000486618	0	Cg_03_90	ribosomal protein S16
M896_031430	0.002043423	Inf	Cg_03_91	subunit F of V-type ATPase
M896_031440	0.004921005	0.72120383	Cg_03_92	SCF ubiquitin ligase Skp1-like protein
M896_031460	0.001510721	2.42826583	Cg_03_94	UTP-glucose-1-phosphate uridylyltransferase
M896_031470	0.002149518	5.5592077	Cg_03_95	subunit gamma of vesicle coat complex
M896_031480	0	Inf	Cg_03_96	hypothetical protein
M896_040020	0.001807643	0.71883382	Cg_05F_3	PCI domain-containing protein
M896_040030	0.005754183	0.33839177	Cg_04_212	hypothetical protein

M896_040040	0.001423761	0.49895941	Cg_04_211	hypothetical protein
M896_040050	0.002361615	Inf	Cg_04_210	translation initiation factor IF-2
M896_040060	0.001446759	Inf	Cg_04_209	hypothetical protein
M896_040070	0.0013225	Inf	Cg_04_208	hypothetical protein
M896_040080	0.005158449	0.53226758	Cg_04_207	hypothetical protein
M896_040090	0.005544467	0.39772727	Cg_04_206	hypothetical protein
M896_040100	0.003005904	0.10359702	Cg_04_205	hypothetical protein
M896_040110	0.003654756	Inf	Cg_04_204	ER translocation protein Sec61
M896_040120	0.003222703	Inf	Cg_04_203	O-sialoglycoprotein endopeptidase
M896_040130	0.00351311	2.99419921	Cg_04_202	HrpA-like helicase
M896_040140	0.002764084	2.96730311	Cg_04_201	hypothetical protein
M896_040150	0.002401302	Inf	Cg_04_200	Rab GTPase
M896_040160	0.003241754	Inf	Cg_04_199	hypothetical protein
M896_040170	0.003004535	0.43953451	Cg_04_198	hypothetical protein
M896_040180	0.001505249	0.89562584	Cg_04_197	hypothetical protein
M896_040190	0.000925926	0.38816456	Cg_04_196	hypothetical protein
M896_040200	0.001931114	Inf	Cg_04_195	hypothetical protein
M896_040210	0	Inf	Cg_04_194	hypothetical protein
M896_040220	0.000991543	Inf	Cg_04_193	hypothetical protein
M896_040230	0.002147947	0.99031166	Cg_04_192	hypothetical protein
M896_040240	0.000855503	0.90214339	Cg_04_191	hypothetical protein
M896_040250	0.00262674	1.28482099	Cg_04_190	TATA binding protein associated factor 4-like protein
M896_040260	0.002548785	0.24664879	Cg_04_189	hypothetical protein
M896_040270	0.003047351	0.22691766	Cg_04_188	hypothetical protein
M896_040280	0.001674969	0.3179821	Cg_04_187	Ca2+-binding actin-bundling fimbrin/plastin

M896_040290	0.003242148	1.26786759	Cg_04_186	hypothetical protein
M896_040300	0.001525054	3.94695854	Cg_04_185	telomerase reverse transcriptase
M896_040310	0.001856738	0.8822441	Cg_04_184	hypothetical protein
M896_040320	0.001374225	Inf	Cg_04_183	subunit alpha of proteasome
M896_040330	0.00132716	Inf	Cg_04_182	DWNN ubiquitin-like domain-containing protein
M896_040340	0.003854694	2.00255845	Cg_04_181	pre-ribosome nuclear export protein
M896_040350	0.001738473	0.27702905	Cg_04_180	hypothetical protein
M896_040360	0.003006967	Inf	Cg_04_179	hypothetical protein
M896_040370	0.002973978	0.39107418	Cg_04_178	hypothetical protein
M896_040390	0.002188552	0.28644435	Cg_04_177	subunit alpha of mRNA capping enzyme
M896_040410	0.002578959	2.3392881	Cg_04_176	superfamily II DNA/RNA helicase
M896_040420	0.002200527	Inf	Cg_04_175	hypothetical protein
M896_040430	0.008112875	0.11226872	Cg_04_174	hypothetical protein
M896_040440	0.002505202	1.39214713	Cg_04_173	type-B delta catalytic subunit of DNA polymerase
M896_040450	0.001851852	Inf	Cg_04_172	histone H4
M896_040460	0.007626886	1.10966844	Cg_04_171	histone H3
M896_040470	0.000940623	Inf	Cg_04_170	hypothetical protein
M896_040480	0	Inf	Cg_04_168	hypothetical protein
M896_040490	0.001423886	Inf	Cg_04_167	subunit beta of T complex protein 1
M896_040500	0.002386831	1.05284068	Cg_04_166	hypothetical protein
M896_040510	0.001362567	0.0461944	Cg_04_165	hypothetical protein
M896_040520	0.001959379	0.76876877	Cg_04_164	hypothetical protein
M896_040530	0.001297879	Inf	Cg_04_163	hypothetical protein
M896_040540	0.001921042	Inf	Cg_04_162	hypothetical protein

M896_040550	0.004204204	Inf	Cg_04_160	ribosomal protein S29
M896_040560	0	Inf	Cg_04_159	hypothetical protein
M896_040570	0	Inf	Cg_04_158	ribosomal protein S5
M896_040580	0.002659069	0.265625	Cg_04_157	hypothetical protein
M896_040590	0.001916602	0.31062626	Cg_04_155	hypothetical protein
M896_040610	0.003116385	3.42480298	Cg_04_154	hypothetical protein
M896_040620	0.000819252	Inf	Cg_04_153	transcription elongation factor S-II
M896_040630	0.000927472	Inf	Cg_04_152	hypothetical protein
M896_040650	0.003744856	0.34888602	Cg_04_151	protein phosphatase 2A
M896_040660	0.001186624	0.40462376	Cg_04_150	putative glucose transporter
M896_040670	0.001545103	0.41454023	Cg_04_149	SAGA/ADA complex histone acetyltransferase
M896_040680	0.003560149	1.95512985	Cg_04_148	hypothetical protein
M896_040690	0.002147469	0.5280638	Cg_04_147	HrpA-like helicase
M896_040700	0.001634738	0.85954927	Cg_04_146	DHHC-type Zn finger domain-containing protein
M896_040710	0.002134338	Inf	Cg_04_145	hypothetical protein
M896_040720	0.002222222	Inf	Cg_04_144	RNA polymerase II transcription factor
M896_040740	0.00140802	Inf	Cg_04_143	protein tyrosine phosphatase
M896_040750	0.00141759	1.46713313	Cg_04_142	26S proteasome regulatory complex protein
M896_040760	0.001378122	Inf	Cg_04_141	hypothetical protein
M896_040770	0.00667334	0.83003195	Cg_04_140	ribosomal protein L27
M896_040780	0.00359147	0.28275266	Cg_04_139	hypothetical protein
M896_040790	0.002731567	1.86831108	Cg_04_138	DNA topoisomerase II
M896_040800	0.000416667	Inf	Cg_04_137	hypothetical protein
M896_040810	0.001453224	0.1277081	Cg_04_136	hypothetical protein
M896_040820	0.002334267	Inf	Cg_04_135	hypothetical protein

M896_040840	0.001267057	Inf	Cg_04_130	heat shock transcription factor
M896_040850	0.00301619	0.56260042	Cg_04_129	hypothetical protein
M896_040860	0.000888889	Inf	Cg_04_128	hypothetical protein
M896_040870	0.002704779	Inf	Cg_04_126	non-ATPase subunit 7 of 26S proteasome
M896_040880	0.001952862	0.61713228	Cg_04_125	hypothetical protein
M896_040890	0.000333667	Inf	Cg_04_124	hypothetical protein
M896_040900	0.000815794	Inf	Cg_04_123	ribosomal protein SA
M896_040920	0.002432691	0.68861602	Cg_04_122	dimethyladenosine transferase
M896_040930	0.003731343	1.16044757	Cg_04_121	hypothetical protein
M896_040940	0.000457081	0.08975059	Cg_04_120	MdlB-type ABC transporter
M896_040960	0.001405358	0.47366091	Cg_04_119	E3 ubiquitin-protein ligase
M896_040970	0.00342241	0.51423614	Cg_04_118	hypothetical protein
M896_040980	0.000663746	0.13662266	Cg_04_117	putative RNA-binding protein
M896_040990	0.0015273	1.33975388	Cg_04_116	hypothetical protein
M896_041010	0.000771605	Inf	Cg_04_115	uridine diphosphate-N-acetylglucosamine
M896_041020	0	Inf	Cg_04_114	hypothetical protein
M896_041030	0.002085994	Inf	Cg_04_113	hypothetical protein
M896_041040	0.002496329	0.73675944	Cg_04_112	lysyl-tRNA synthetase
M896_041050	0.003024575	0.63961461	Cg_04_111	X-prolyl aminopeptidase
M896_041060	0.003735632	0.42403249	Cg_04_110	hypothetical protein
M896_041070	0.001576504	Inf	Cg_04_109	putative methyltransferase
M896_041080	0.001910086	Inf	Cg_04_108	ubiquitin-conjugating enzyme E2
M896_041090	0.00237037	0.23853211	Cg_04_107	ribosomal protein S11
M896_041100	0.003342693	1.02291845	Cg_04_106	putative lipase
M896_041110	0.001092459	0.15657745	Cg_04_105	putative dolichol kinase
M896_041120	0.002868555	Inf	Cg_04_104	Rab11-like GTPase

M896_041130	0.002149871	0.46836677	Cg_04_103	vacuolar protein sorting-associated protein
M896_041140	0.002007869	0.14252772	Cg_04_102	PP2A-like phosphoprotein phosphatase
M896_041150	0.002080378	0.42932347	Cg_04_101	mitotic phase inducer phosphatase
M896_041160	0.001897019	Inf	Cg_04_100	peptidyl-tRNA hydrolase
M896_041170	0.002429943	0.51053806	Cg_04_99	putative 6-phosphogluconolactonase
M896_041180	0.007149758	0.76205358	Cg_04_98	ribosomal protein L22
M896_041190	0.003816993	0.35022845	Cg_04_97	seryl-tRNA synthetase
M896_041200	0.002031122	0.58634839	Cg_04_96	subunit of U5 snRNP spliceosome
M896_041210	0.000495893	Inf	Cg_04_95	hypothetical protein
M896_041220	0.001399556	Inf	Cg_04_94	translation initiation factor 6
M896_041230	0.001559454	Inf	Cg_04_93	Sm-like ribonucleoprotein
M896_041240	0.002678385	0.13758957	Cg_04_92	hypothetical protein
M896_041260	0.004575163	0.92915811	Cg_04_89	Nop56p-like protein
M896_041270	0.004063986	Inf	Cg_04_88	hypothetical protein
M896_041280	0.002012882	1.09930716	Cg_04_87	asparagine synthase
M896_041290	0.00151005	1.31535173	Cg_04_86	minichromosome maintenance protein
M896_041300	0.005291005	0.79615967	Cg_04_85	hypothetical protein
M896_041310	0.002512179	0.44256414	Cg_04_84	cohesin
M896_041320	0.003006967	Inf	Cg_04_83	hypothetical protein
M896_041330	0.002155716	Inf	Cg_04_82	pseudouridine synthase
M896_041340	0.003653942	0.51168871	Cg_04_81	subunit beta of phenylalanyl-tRNA synthetase
M896_041350	0.002024804	0.66373703	Cg_04_79	superfamily II RNA helicase
M896_041370	0.007380174	2.41681575	Cg_04_78	hypothetical protein
M896_041380	0.002446103	0.9068609	Cg_04_77	chromosome segregation ATPase
M896_041390	0.000984529	Inf	Cg_04_76	subunit of endoplasmic reticulum translocation complex

M896_041400	0.001742919	Inf	Cg_04_75	subunit of transcription initiation factor TFIID
M896_041410	0.004840484	Inf	Cg_04_74	hypothetical protein
M896_041420	0.006887589	0.96466067	Cg_04_73	homeodomain-containing protein
M896_041430	0.001804589	2.84915994	Cg_04_72	signal recognition particle GTPase
M896_041440	0.000522108	0	Cg_04_71	hypothetical protein
M896_041450	0.002253997	1.6988106	Cg_04_70	myosin heavy chain
M896_041460	0	Inf	Cg_04_69	ribosomal protein L29e
M896_041470	0.001458151	Inf	Cg_04_68	hypothetical protein
M896_041480	0.002469136	Inf	Cg_04_67	ribosomal protein S27
M896_041490	0.001021115	Inf	Cg_04_66	subunit theta of T-complex protein 1
M896_041500	0.000560224	Inf	Cg_04_65	homeodomain-containing protein
M896_041510	0.004208099	Inf	Cg_04_64	hypothetical protein
M896_041520	0.004206676	0.24946439	Cg_04_63	hypothetical protein
M896_041530	0.003128371	Inf	Cg_04_62	dolichol-phosphate mannosyltransferase
M896_041540	0.001649732	0.55547804	Cg_04_61	topoisomerase IA
M896_041550	0.000486618	Inf	Cg_04_59	hypothetical protein
M896_041560	0.005201966	0.39446158	Cg_04_58	actin-like protein
M896_041570	0.001879729	0.86292611	Cg_04_56	translation elongation factor EF-1 alpha
M896_041580	0.002161506	0.17543388	Cg_04_55	subunit A of DNA topoisomerase VI
M896_041590	0.004670913	0.77369041	Cg_04_54	hypothetical protein
M896_041600	0.002418226	0.67363933	Cg_04_53	alpha-1%2C2 mannosyl-transferase
M896_041610	0.002539182	0.66975432	Cg_04_52	valyl-tRNA synthetase
M896_041620	0.002039721	0.19669692	Cg_04_51	hypothetical protein
M896_041630	0.004541591	0.29956319	Cg_04_50	subunit beta of pyruvate dehydrogenase
M896_041640	0	Inf	Cg_04_49	translation initiation factor IF1A
M896_041650	0.003414352	0.49215792	Cg_04_48	amino acid permease

M896_041660	0.001863499	0.11052669	Cg_04_47	SWIB domain-containing protein
M896_041670	0.003128594	0.27003078	Cg_04_46	ubiquitin fusion degradation protein 2
M896_041680	0.001284958	Inf	Cg_04_45	thymidylate kinase
M896_041690	0.00302204	0.25655053	Cg_04_44	hypothetical protein
M896_041700	0.001311359	Inf	Cg_04_43	hypothetical protein
M896_041710	0.001079797	Inf	Cg_04_42	WD40 domain-containing protein
M896_041720	0.003420875	2.74933679	Cg_04_41	nuclear protein export factor
M896_041730	0.002432825	Inf	Cg_04_40	hypothetical protein
M896_041740	0.001295592	Inf	Cg_04_39	lysophospholipid acyltransferase
M896_041750	0.001531295	2.33281493	Cg_04_38	superfamily II RNA helicase
M896_041760	0.000883691	0.62480102	Cg_04_37	putative RNA-binding protein
M896_041770	0.001670844	Inf	Cg_04_36	ribosomal protein L32
M896_041780	0.001044216	Inf	Cg_04_35	hypothetical protein
M896_041790	0.003333333	0.11269959	Cg_04_33	hypothetical protein
M896_041800	0.000979968	0.09126816	Cg_04_32	hypothetical protein
M896_041810	0.002276867	0.12599772	Cg_04_31	hypothetical protein
M896_041820	0.002035002	0	Cg_04_30	ribosomal protein S10
M896_041840	0.001593999	0.23743637	Cg_04_29	hypothetical protein
M896_041850	0.00308642	Inf	Cg_04_28	ribosomal protein L13a
M896_041860	0.001970055	Inf	Cg_04_27	hypothetical protein
M896_041870	0.00174526	1.08449703	Cg_04_26	subunit beta' of DNA-directed RNA polymerase
M896_041890	0.002682895	1.30147277	Cg_04_25	hypothetical protein
M896_041910	0.002934564	Inf	Cg_04_24	hypothetical protein
M896_041920	0.005372405	0.7063997	Cg_04_23	hypothetical protein
M896_041930	0.003106332	3.45880462	Cg_04_22	hypothetical protein

M896_041940	0.002939447	0	Cg_04_21	hypothetical protein
M896_041950	0.00198059	Inf	Cg_04_20	TATA-box binding protein
M896_041960	0.001446759	Inf	Cg_04_19	hypothetical protein
M896_041970	0.001486126	Inf	Cg_04_18	hypothetical protein
M896_041980	0.001063727	Inf	Cg_04_17	Na+/H+ antiporter
M896_041990	0.002660913	0.25626779	Cg_04_16	Zn-finger protein
M896_042000	0.001832917	0.76631942	Cg_04_15	hypothetical protein
M896_042010	0.001128748	0.3680418	Cg_04_14	regulator of chromosome condensation
M896_042020	0.000251572	0	Cg_04_13	tRNA-binding domain-containing CsaA-like protein
M896_042030	0.001171257	Inf	Cg_04_12	hypothetical protein
M896_042040	0.002762084	0.27719767	Cg_04_11	GTPase activating protein
M896_042050	0.001514221	0.19164691	Cg_04_10	hypothetical protein
M896_042060	0.004901961	1.41350803	Cg_04_9	subunit beta of mRNA capping enzyme
M896_042070	0.003537866	Inf	Cg_04_8	GTP-binding nuclear protein
M896_042080	0.000555556	Inf	Cg_04_7	hypothetical protein
M896_042090	0.000424798	0	Cg_04_6	hypothetical protein
M896_042100	0.001011941	Inf	Cg_04_5	hypothetical protein
M896_042110	0	Inf	Cg_04_4	hypothetical protein
M896_042120	0.001572723	1.13738237	Cg_04_3	hypothetical protein
M896_042130	0.001814059	0.1280262	Cg_04_2	hypothetical protein
M896_042140	0	Inf	Cg_04_1	hypothetical protein
M896_050040	0	Inf	Cg_05A_60	subunit D of vacuolar-type H+-ATPase
M896_050050	0.000627746	Inf	Cg_05A_59	hypothetical protein
M896_050080	0.002239906	1.02774247	Cg_05A_56	signal recognition particle GTPase
M896_050090	0.003808328	Inf	Cg_05A_55	ribose 5-phosphate isomerase

M896_050100	0.003568202	Inf	Cg_05A_54	subunit Rpn7 of 26S proteasome regulatory complex
M896_050120	0.002968961	0.82949551	Cg_05A_53	hypothetical protein
M896_050130	0.002247547	0.11092437	Cg_05A_52	subunit C34 of DNA-directed RNA polymerase III
M896_050140	0.000361337	Inf	Cg_05A_51	hypothetical protein
M896_050150	0.002743484	0	Cg_05A_50	hypothetical protein
M896_050160	0.002018822	Inf	Cg_05A_49	subunit gamma of T-complex protein 1
M896_050180	0	Inf	Cg_05A_47	histone H3/H4-like protein
M896_050190	0.002524312	0.36207778	Cg_05A_46	RNase PH-like protein
M896_050200	0.000896735	0.69095255	Cg_05A_45	putative P-loop ATPase/acetyltransferase fusion protein
M896_050210	0.002760943	0.43682144	Cg_05A_44	U1 snRNP-specific protein C
M896_050230	0.001099537	Inf	Cg_05A_43	acyl-CoA cholesterol acyltransferase
M896_050240	0.002159704	Inf	Cg_05A_42	1-acyl-SN-glycerol-3-phosphate acyltransferase
M896_050250	0.001571268	1.26338924	Cg_05A_41	kinesin-like protein
M896_050260	0.001185185	0.2345679	Cg_05A_40	hypothetical protein
M896_050270	0.000768403	0	Cg_05A_39	inorganic pyrophosphatase
M896_050280	0.001427469	Inf	Cg_05A_37	Rho GTPase
M896_050290	0.001944444	0	Cg_05A_36	hypothetical protein
M896_050300	0	Inf	Cg_05A_35	putative actin depolymerization factor
M896_050310	0.002867384	Inf	Cg_05A_34	hypothetical protein
M896_050320	0.002145002	0.50040225	Cg_05A_33	mRNA capping enzyme
M896_050330	0.000973007	0.38836256	Cg_05A_32	hypothetical protein
M896_050340	0.006688778	1.30876875	Cg_05A_31	ribosomal protein S23

M896_050350	0.000620516	Inf	Cg_05A_30	regulatory complex subunit of 26S proteasome
M896_050360	0.002225209	Inf	Cg_05A_29	ATP/ADP translocase
M896_050370	0.001224847	Inf	Cg_05A_28	hypothetical protein
M896_050380	0.002279202	0.90564593	Cg_05A_27	ATP-dependent rRNA helicase
M896_050390	0.003697125	2.64838989	Cg_05A_26	hypothetical protein
M896_050410	0.003652263	Inf	Cg_05A_25	fragile histidine family hydrolase
M896_050420	0.001172928	Inf	Cg_05A_24	hypothetical protein
M896_050430	0.00285748	0.93231785	Cg_05A_23	hypothetical protein
M896_050440	0.003355371	0.43757375	Cg_05A_22	ATP/ADP translocase
M896_050450	0.002603969	0.38895317	Cg_05A_21	uracil-DNA glycosylase
M896_050460	0.001851852	1.05547445	Cg_05A_20	ATP/ADP translocase
M896_050470	0.000881834	0	Cg_05A_19	subunit alpha of 20S proteasome
M896_050480	0.003244838	1.37634137	Cg_05A_18	putative short chain dehydrogenase
M896_050490	0.000750117	Inf	Cg_05A_17	AGC serine/threonine kinase
M896_050500	0.00333855	0.27896841	Cg_05A_16	putative TFIIF-interacting CTD phosphatase
M896_050510	0.003120796	0.5671125	Cg_05A_15	phosphoinositide 3-kinase
M896_050520	0.000864895	1.51794341	Cg_05A_13	replication factor A protein 1
M896_050530	0	Inf	Cg_05A_12	hypothetical protein
M896_050540	0.000887166	0.46113687	Cg_05A_11	importin
M896_050550	0.00281294	1.48291408	Cg_05A_10	subunit eta of T-complex protein 1
M896_050570	0.003552251	1.03536676	Cg_05A_7	hypothetical protein
M896_050580	0.000532141	Inf	Cg_05A_6	MYST histone acetyltransferase
M896_050590	0	Inf	Cg_05A_5	ribosomal biogenesis protein
M896_050600	0.000874349	0.62142295	Cg_05A_4	HrpA-like helicase
M896_050610	0.003292181	0.34450356	Cg_05A_3	ATP-dependent RNA helicase

M896_050620	0.00045819	Inf	Cg_05A_2	subunit of translation initiation factor 2B
M896_050630	0.001965056	0.48955817	Cg_05D_1	MutS-like mismatch repair ATPase
M896_050640	0.003125	0.78765761	Cg_05D_2	hypothetical protein
M896_050650	0.00328673	0.5132996	Cg_05D_3	hypothetical protein
M896_050660	0.002862986	1.27862187	Cg_05D_4	methionine aminopeptidase 2
M896_050670	0.003890924	1.15798005	Cg_05D_5	hypothetical protein
M896_050680	0.002991823	1.42208001	Cg_05D_6	subunit of transcription initiation factor TFIID
M896_050690	0.002373247	0	Cg_05D_7	hypothetical protein
M896_050700	0.002030344	0.67762628	Cg_05D_8	small subunit of replication factor C
M896_050710	0.002540705	0.63141725	Cg_05D_9	hypothetical protein
M896_050720	0	Inf	Cg_05D_10	putative methyltransferase
M896_050740	0.001851852	2.9941245	Cg_05D_12	chromosome segregation protein
M896_050750	0.002851153	Inf	Cg_05D_13	fibrillarin
M896_050760	0.00109979	0.08508698	Cg_05D_14	HMG-like nuclear protein
M896_050770	0.002310858	0.50527682	Cg_05D_15	subunit of U1 snRNP
M896_050780	0.001415146	0.27428768	Cg_05D_16	hypothetical protein
M896_050790	0.002362708	Inf	Cg_05D_17	hypothetical protein
M896_050800	0.00216644	0.58934642	Cg_05D_18	glycerol-3-phosphate dehydrogenase
M896_050810	0.001595026	Inf	Cg_05D_19	hypothetical protein
M896_050820	0.002350932	1.69732892	Cg_05D_20	putative RNA-processing beta-lactamase
M896_050830	0.00191752	Inf	Cg_05D_21	long-chain acyl-CoA synthetase
M896_050840	0.000883838	Inf	Cg_05D_22	ribonucleoside-diphosphate reductase
M896_050850	0.000441501	Inf	Cg_05D_23	ubiquitin-conjugating enzyme
M896_050860	0.002555772	Inf	Cg_05D_24	hypothetical protein
M896_050870	0.006814815	Inf	Cg_05D_25	putative kinase
M896_050880	0.002116402	1.03490816	Cg_05D_26	hypothetical protein

M896_050890	0.000243309	Inf	Cg_05D_27	hypothetical protein
M896_050900	0.001564945	Inf	Cg_05D_28	ribosomal protein L27
M896_050910	0.003713176	1.99819147	Cg_05D_29	kinesin-like protein
M896_050920	0.004002389	Inf	Cg_05D_30	hypothetical protein
M896_050930	0.001880053	0.53012685	Cg_05D_31	hypothetical protein
M896_050940	0.002558666	0.81698184	Cg_05D_32	hypothetical protein
M896_050960	0.000580304	Inf	Cg_05D_33	subunit B of V-type ATP synthase
M896_050970	0.010185185	0.35235096	Cg_05D_34	DNA directed RNA polymerase
M896_050980	0.002743484	Inf	Cg_05D_35	hypothetical protein
M896_050990	0.001392111	1.88469134	Cg_05D_36	phosphoglyceromutase
M896_051000	0.003703704	Inf	Cg_05D_37	hypothetical protein
M896_051010	0.003632819	0.83979445	Cg_05D_38	subunit B of DNA polymerase alpha
M896_051020	0.005770411	1.06691919	Cg_05D_39	chromatin remodeling protein
M896_051030	0.003600823	0.27890121	Cg_05D_40	DnaJ-like protein
M896_051040	0.001117818	0.12805392	Cg_05D_41	polyadenylate-binding protein
M896_051060	0.000549511	Inf	Cg_05D_42	subunit of transcription initiation factor TFIID
M896_051070	0.001303449	0.16200789	Cg_05D_43	subunit 2 of origin recognition complex
M896_051080	0.002426694	1.17790364	Cg_05D_44	guanine nucleotide exchange factor
M896_051090	0.002710716	0.48039477	Cg_05D_45	hypothetical protein
M896_051100	0.002436647	Inf	Cg_05D_46	hypothetical protein
M896_051110	0.002258831	0.95240851	Cg_05D_47	hypothetical protein
M896_051120	0.002729045	0.50554899	Cg_05D_48	Rad14-like DNA excision repair protein
M896_051130	0.001268861	Inf	Cg_05D_49	ubiquitin fusion-degradation protein
M896_051140	0.002956803	1.77423768	Cg_05D_50	hypothetical protein
M896_051150	0.001595442	Inf	Cg_05D_51	subunit beta of casein kinase II
M896_051160	0.00193613	1.03147387	Cg_05D_52	DNA polymerase epsilon

M896_051170	0	Inf	Cg_05D_53	hypothetical protein
M896_051180	0.003748446	3.53379156	Cg_05D_54	importin beta
M896_051190	0.002095113	0.28421181	Cg_05D_55	hypothetical protein
M896_051200	0.001152263	Inf	Cg_05D_56	lysophospholipid acyltransferase
M896_051210	0.001239057	0.52697981	Cg_05D_57	PP2A-like protein phosphatase
M896_051220	0.002300437	Inf	Cg_05D_58	hypothetical protein
M896_051230	0	Inf	Cg_05D_59	ribosomal protein L44
M896_051240	0.001338688	Inf	Cg_05D_60	ubiquitin-conjugating enzyme E2
M896_051250	0.00137741	1.19970799	Cg_05D_61	SNF2-like helicase
M896_051330	0.002935863	Inf	Cg_05D_62	nucleotide excision repair endonuclease Nef1
M896_051340	0.001817558	0.51592582	Cg_05D_63	Rrp4-like RNA-binding protein
M896_051350	0.002727414	1.65511061	Cg_05D_64	putative RNA-processing beta-lactamase
M896_051360	0.001362963	Inf	Cg_05D_65	hypothetical protein
M896_051370	0.004280328	1.08269922	Cg_05D_66	phosphatidylinositol 5-phosphate phosphatase
M896_051380	0.00194298	0.89140133	Cg_05D_67	ubiquitin-protein ligase
M896_051390	0.000560917	Inf	Cg_05D_68	hypothetical protein
M896_051400	0.001054131	0.93842802	Cg_05D_69	hypothetical protein
M896_051410	0.0022087	0.27145504	Cg_05D_70	hypothetical protein
M896_051420	0.000363306	Inf	Cg_05D_71	regulatory subunit of ATP-dependent 26S proteasome
M896_051430	0.003003003	1.37624386	Cg_05D_72	chromatin remodeling transcription factor
M896_051440	0.001224105	Inf	Cg_05D_73	PAP2-like phosphatidic acid phosphatase
M896_051450	0	Inf	Cg_05D_74	beta type-6 subunit of proteasome
M896_051460	0.002469136	1.0318323	Cg_05D_75	glutaminyl-tRNA synthetase
M896_051470	0	Inf	Cg_05D_76	hypothetical protein

M896_051480	0.001977796	0.89979039	Cg_05D_77	insulinase-like Zn-dependent peptidase
M896_051490	0	Inf	Cg_05D_78	putative homeodomain-containing transcription factor
M896_051500	0.001076658	Inf	Cg_05D_79	hypothetical protein
M896_051510	0.000818382	0.77131783	Cg_05D_80	hypothetical protein
M896_051520	0.001615368	0.19317147	Cg_05D_81	hypothetical protein
M896_051550	0.00405855	0.73268293	Cg_05E_2	White-like ABC transporter
M896_051560	0	Inf	Cg_05E_3	hypothetical protein
M896_051570	0.002564103	0.41585815	Cg_05E_4	ubiquitin-protein ligase
M896_051580	0.001833517	0	Cg_05E_5	hypothetical protein
M896_051590	0.001413627	0	Cg_05E_6	ribosomal protein S24
M896_051610	0.002973777	1.48231848	Cg_05E_7	subunit D2 of Sm-like ribonucleoprotein
M896_051620	0.001304121	0	Cg_05E_8	choline-phosphate cytidylyltransferase
M896_051630	0.001006441	Inf	Cg_05E_9	Mnd1-like meiotic recombination protein
M896_051640	0.003703704	1.80503145	Cg_05E_10	hypothetical protein
M896_051650	0.005175038	Inf	Cg_05E_11	hypothetical protein
M896_051660	0.001771801	0.95284924	Cg_05E_12	hypothetical protein
M896_051670	0.001108958	0.21013728	Cg_05E_13	hypothetical protein
M896_051680	0.002388889	0.26201754	Cg_05E_15	spore wall protein Swp1b
M896_051690	0.001831591	0.07420233	Cg_05E_16	ATP-dependent RNA helicase
M896_051700	0.002391789	0.16250365	Cg_05E_17	enolase
M896_051710	0.005787037	0.5467944	Cg_05E_18	hypothetical protein
M896_051720	0.001298929	0.29759001	Cg_05E_19	dynamin-like vacuolar protein-sorting protein
M896_051730	0.002394029	0.03013875	Cg_05E_20	hypothetical protein
M896_051740	0.000576132	0	Cg_05E_21	hypothetical protein
M896_051750	0.00333478	0.21413704	Cg_05E_22	cellular morphogenesis/cytokinesis regulation

M896_051760	0.001968906	0.147443	Cg_05E_23	Spc97/Spc98-like spindle pole body protein
M896_051770	0.000589971	0.26571251	Cg_05E_24	cyclophilin-type peptidylprolyl cis-trans
M896_051780	0.002575827	0.4405346	Cg_05E_25	leucyl aminopeptidase
M896_051790	0.003257158	0.21847203	Cg_05E_26	hypothetical protein
M896_051800	0.000794566	0.17005697	Cg_05E_27	glycyl-tRNA synthetase
M896_051810	0.001151298	Inf	Cg_05E_28	hypothetical protein
M896_051820	0.003106332	3.38372962	Cg_05E_29	hypothetical protein
M896_051830	0.002270012	Inf	Cg_05E_30	hypothetical protein
M896_051840	0.001448954	1.32253913	Cg_05E_31	elongation factor 3
M896_051850	0.001776163	0.1968006	Cg_05E_32	putative DNA primase
M896_051860	0.003578458	0.24365821	Cg_05E_33	putative subunit of t-SNARE complex
M896_051870	0	Inf	Cg_05E_34	putative G10 protein
M896_051880	0.002338494	0.17138373	Cg_05E_35	putative gamma-glutamyltransferase
M896_051890	0.001624431	0.19269358	Cg_05E_36	hypothetical protein
M896_051900	0.002819092	Inf	Cg_05E_37	putative ATP-dependent RNA helicase
M896_051910	0.002228369	0.28257284	Cg_05E_39	putative subunit of Mre11
M896_051930	0.002379451	0.52286545	Cg_05F_27	hypothetical protein
M896_051940	0	Inf	Cg_05F_26	LSM domain-containing protein
M896_051950	0.001490248	Inf	Cg_05F_25	ribosomal protein S4-like protein
M896_051960	0.004923999	Inf	Cg_05F_24	putative DNA replication protein kinase
M896_051970	0.003179145	0.26173285	Cg_05F_23	alpha subunit of proteasome
M896_051980	0	Inf	Cg_05F_22	PUA domain-containing protein
M896_052000	0.003379883	0.34588929	Cg_05F_21	putative nucleotidyl transferase
M896_052010	0.000912242	0.26786954	Cg_05F_20	putative Zn-dependent protease
M896_052020	0.003609701	0.69529453	Cg_05F_19	glutaredoxin domain-containing protein
M896_052030	0.002291896	0.13797437	Cg_05F_18	peptidase M48 domain-containing protein

M896_052040	0.00146871	0	Cg_05F_17	putative alpha subunit of proteasome
M896_052050	0.003398118	0.26258776	Cg_05F_16	hypothetical protein
M896_052060	0.001061498	0	Cg_05F_15	hypothetical protein
M896_052070	0.001861547	0.13469207	Cg_05F_14	hypothetical protein
M896_052080	0.000831444	Inf	Cg_05F_13	hypothetical protein
M896_052100	0.000718629	0.31745836	Cg_05F_12	hypothetical protein
M896_052110	0.000519818	0	Cg_05F_11	hypothetical protein
M896_052120	0	Inf	Cg_05F_10	hypothetical protein
M896_052130	0	Inf	Cg_05F_9	ribosomal protein L30E
M896_052140	0	Inf	Cg_05F_8	hypothetical protein
M896_052150	0.000269974	Inf	Cg_05F_7	hypothetical protein
M896_052160	0.001527778	0.39621314	Cg_05F_6	protein kinase domain-containing protein
M896_052170	0.001819813	0.14356279	Cg_05F_5	acyl-CoA thioesterase domain-containing protein
M896_052180	0.001711459	0.42695421	Cg_05F_4	DNA replication factor RFC1
M896_060010	0.000569801	Inf	Cg_07_67	putative zinc finger protein
M896_060020	0.001386182	Inf	Cg_07_66	hypothetical protein
M896_060050	0.010344288	0.55279732	Cg_06A_69	hypothetical protein
M896_060060	0.001870813	Inf	Cg_06A_68	hypothetical protein
M896_060070	0.002786273	Inf	Cg_06A_67	hypothetical protein
M896_060080	0.001196581	Inf	Cg_06A_65	hypothetical protein
M896_060090	0.001664731	1.74155275	Cg_06A_64	transketolase
M896_060100	0.002792957	0.76660959	Cg_06A_63	hypothetical protein
M896_060110	0.004248717	0.50189419	Cg_06A_62	hypothetical protein
M896_060120	0.001317217	0.13156376	Cg_06A_61	hypothetical protein
M896_060130	0	Inf	Cg_06A_60	hypothetical protein

M896_060140	0.004152637	0.19490587	Cg_06A_59	hypothetical protein
M896_060150	0.002514861	0.20869068	Cg_06A_58	hypothetical protein
M896_060160	0.002279202	0.44438819	Cg_06A_57	hypothetical protein
M896_060170	0.002536783	Inf	Cg_06A_56	hypothetical protein
M896_060180	0.001811374	0.52133917	Cg_06A_55	hypothetical protein
M896_060190	0.004643449	0.27754237	Cg_06A_54	hypothetical protein
M896_060200	0.000899165	0	Cg_06A_53	putative ATPase
M896_060210	0.002003888	0.1032121	Cg_06A_52	Rad3-like DNA repair helicase
M896_060220	0.000770077	0	Cg_06A_51	subunit beta of translation initiation factor 2
M896_060230	0.001969395	0.31638808	Cg_06A_50	beta-tubulin folding cofactor D
M896_060240	0.003142536	0.39166299	Cg_06A_49	hypothetical protein
M896_060250	0.001558016	0.19113785	Cg_06A_48	polar tube protein 2
M896_060260	0.002463353	1.15368148	Cg_06A_47	polar tube protein 1
M896_060270	0.000925926	Inf	Cg_06A_46	putative E2F transcription factor
M896_060280	0	Inf	Cg_06A_45	subunit of transport protein particle complex
M896_060290	0.006713717	0.2703444	Cg_06A_44	leucyl-tRNA synthetase
M896_060300	0.002363453	1.07452316	Cg_06A_42	hypothetical protein
M896_060310	0.003462158	0.72705315	Cg_06A_41	hypothetical protein
M896_060320	0.001414898	0.43352872	Cg_06A_40	queuine tRNA-ribosyltransferase
M896_060330	0	Inf	Cg_06A_39	RNA polymerases N
M896_060340	0.001782233	Inf	Cg_06A_38	PPIase/rotamase
M896_060350	0.001663818	1.19491272	Cg_06A_37	minichromosome maintenance protein
M896_060360	0.002469136	0.14871691	Cg_06A_36	subunit of transcription initiation factor IIE
M896_060370	0.002401488	Inf	Cg_06A_35	single-stranded DNA-binding replication protein
M896_060380	0.001624631	1.22378038	Cg_06A_34	hypothetical protein

M896_060390	0.005902472	1.32315907	Cg_06A_33	hypothetical protein
M896_060400	0.000350877	Inf	Cg_06A_32	RNase PH-like exoribonuclease
M896_060410	0.001893939	0.28099508	Cg_06A_31	Mn2+-dependent serine/threonine protein kinase
M896_060430	0.00291439	0.55951847	Cg_06A_30	subunit of dynactin complex
M896_060450	0.001341574	0.40288084	Cg_06A_29	ribosome biogenesis GTP-binding protein
M896_060460	0.002572016	0.30843009	Cg_06A_28	dUTPase
M896_060470	0.00100852	1.14969295	Cg_06A_27	hypothetical protein
M896_060480	0.00342556	0.63800148	Cg_06A_26	hypothetical protein
M896_060490	0.001006441	Inf	Cg_06A_25	N-acetyltransferase
M896_060500	0.001455884	0.37136439	Cg_06A_24	subunit Nup170 of nuclear pore complex
M896_060510	0.001449848	0.43450142	Cg_06A_23	Tom40-like porin
M896_060520	0.002628903	0.12135686	Cg_06A_22	subunit zeta of T-complex protein 1
M896_060530	0.001927541	0.8319986	Cg_06A_21	hypothetical protein
M896_060540	0.000936569	Inf	Cg_06A_20	Tub-like protein
M896_060550	0.000942418	0	Cg_06A_19	TPR repeat-containing protein
M896_060560	0.001618837	0.09556787	Cg_06A_18	hypothetical protein
M896_060570	0.003689404	0.30218757	Cg_06A_17	hypothetical protein
M896_060580	0.002259402	0	Cg_06A_16	hypothetical protein
M896_060590	0.005333333	1.35234331	Cg_06A_15	hypothetical protein
M896_060600	0.003843123	0.33364757	Cg_06A_14	hypothetical protein
M896_060610	0	Inf	Cg_06A_13	subunit RPB3 of DNA-directed RNA polymerase II
M896_060620	0.004542664	0.23836741	Cg_06A_12	putative HemK-like methylase
M896_060630	0.002440492	1.30994292	Cg_06A_11	histidyl-tRNA synthetase
M896_060640	0.002421164	0.44756506	Cg_06A_10	hypothetical protein

M896_060650	0.003060546	Inf	Cg_06A_8	adenylate kinase
M896_060660	0.003030303	1.16618965	Cg_06A_7	hypothetical protein
M896_060670	0.002120051	0.15895978	Cg_06A_6	hypothetical protein
M896_060680	0.002039118	0	Cg_06A_5	hypothetical protein
M896_060690	0.001913171	0.06732145	Cg_06A_4	hypothetical protein
M896_060700	0.00394714	0.63349224	Cg_06A_3	hypothetical protein
M896_060710	0.002125076	0.46391753	Cg_06A_2	phosphotyrosyl phosphatase activator
M896_060720	0.001709402	0	Cg_06A_1	ribonucleoside-diphosphate reductase
M896_060730	0.00078693	0	Cg_06B_2	hypothetical protein
M896_060740	0.001986928	0.34240302	Cg_06B_3	subunit of vesicle coat complex
M896_060760	0.001758324	0.08136168	Cg_06B_4	symplekin domain-containing protein
M896_060770	0.000711359	Inf	Cg_06B_5	aspartyl-tRNA synthetase
M896_060780	0.001951793	0.21561285	Cg_06B_6	hypothetical protein
M896_060790	0.00242963	0	Cg_06B_7	hypothetical protein
M896_060800	0.001462348	0.3252666	Cg_06B_8	Snf2/Rad54-like helicase
M896_060810	0.001376676	0.14266779	Cg_06B_9	mRNA turnover and stability protein
M896_060820	0	Inf	Cg_06B_10	subunit A of RNA polymerase II
M896_060830	0.002763958	Inf	Cg_06B_12	hypothetical protein
M896_060840	0.001806685	0.33978446	Cg_06B_13	hypothetical protein
M896_060850	0.001972625	1.52461251	Cg_06B_14	subunit Sec63 of preprotein translocase
M896_060860	0.002395587	0.19810666	Cg_06B_15	hypothetical protein
M896_060870	0.002923977	Inf	Cg_06B_16	hypothetical protein
M896_060880	0.000649773	0	Cg_06B_17	ribosomal protein L5
M896_060900	0.001137699	Inf	Cg_06B_18	ubiquitin C-terminal hydrolase
M896_060910	0.002842206	0.44414597	Cg_06B_19	pre-mRNA splicing helicase
M896_060920	0.002343084	0.14323617	Cg_06B_20	P-type ATPase

M896_060930	0.003506889	0.96998833	Cg_06B_22	hypothetical protein
M896_060940	0.001564114	Inf	Cg_06B_23	hypothetical protein
M896_060950	0.001047254	0.3711771	Cg_06B_24	hypothetical protein
M896_060960	0.002506628	0.54879968	Cg_06B_25	hypothetical protein
M896_060970	0.000688365	Inf	Cg_06B_26	subunit epsilon of t-complex protein 1
M896_070050	0.000838574	Inf	Cg_07_65	Myb-like transcription factor
M896_070060	0.002706956	0.09373498	Cg_07_64	RNA 3'-terminal phosphate cyclase
M896_070070	0.000569801	Inf	Cg_07_63	ribulose-5-phosphate 3-epimerase
M896_070080	0.001555556	Inf	Cg_07_62	zinc finger domain-containing protein
M896_070090	0.000387597	0	Cg_07_61	ribosomal protein L19
M896_070100	0.003042328	0.2969919	Cg_07_59	mitochondrial sulfhydryl oxidase
M896_070110	0.000878443	0	Cg_07_58	subunit of transcription initiation factor TFIIIB
M896_070120	0.000779727	0	Cg_07_57	ribosomal protein S18
M896_070130	0.003196881	0	Cg_07_56	ribosomal protein L36
M896_070140	0.001851852	0.15687811	Cg_07_55	hypothetical protein
M896_070150	0.002222222	0	Cg_07_54	hypothetical protein
M896_070160	0	Inf	Cg_07_53	small nuclear ribonucleoprotein
M896_070170	0.002624672	Inf	Cg_07_52	hypothetical protein
M896_070180	0.001571268	0.18671152	Cg_07_51	hypothetical protein
M896_070190	0.001851852	Inf	Cg_07_50	hypothetical protein
M896_070200	0.000346545	Inf	Cg_07_49	GTP-dependent nucleic acid-binding protein EngD
M896_070210	0.003237095	0.99261988	Cg_07_48	hypothetical protein
M896_070220	0.002266446	Inf	Cg_07_47	hypothetical protein
M896_070230	0.005668934	Inf	Cg_07_46	hypothetical protein
M896_070240	0.006385696	Inf	Cg_07_45	hypothetical protein

M896_070250	0.00378174	3.34654261	Cg_07_44	hypothetical protein
M896_070260	0.002203065	0.48298005	Cg_07_43	TruD-like pseudouridine synthase
M896_070270	0.002494331	Inf	Cg_07_42	hypothetical protein
M896_070280	0.001084838	1.52219787	Cg_07_41	ATP-dependent RNA helicase
M896_070300	0.003683684	0.02460154	Cg_07_40	hypothetical protein
M896_070310	0.000654798	0	Cg_07_39	HMG domain-containing chromatin-associated
M896_070320	0.001177146	Inf	Cg_07_38	hypothetical protein
M896_070330	0.002821869	0	Cg_07_37	hypothetical protein
M896_070340	0.002705542	0.32628173	Cg_07_36	ATP-binding protein
M896_070360	0.000914495	Inf	Cg_07_35	ATP-binding protein
M896_070370	0.000974659	0	Cg_07_34	ATP-binding protein
M896_070380	0.001122334	0.12375054	Cg_07_33	subunit DPH2 of diphthamide synthase
M896_070390	0.002825506	0.11768935	Cg_07_32	hypothetical protein
M896_070400	0.002238502	0.16564954	Cg_07_31	hypothetical protein
M896_070410	0	Inf	Cg_07_30	hypothetical protein
M896_070420	0.001939112	0	Cg_07_29	hypothetical protein
M896_070430	0.002809706	0.14628353	Cg_07_28	ferritin
M896_070440	0.001015808	0.12543646	Cg_07_27	hypothetical protein
M896_070450	0.000703497	Inf	Cg_07_26	hypothetical protein
M896_070460	0.002153316	0.26470588	Cg_07_25	hypothetical protein
M896_070470	0.001690689	0.66258694	Cg_07_23	SPX domain-containing vacuolar polyphosphate protein
M896_070480	0.001683502	0.5247296	Cg_07_21	hypothetical protein
M896_070490	0.00272073	0.09908695	Cg_07_20	translation elongation factor EF-1 alpha
M896_070510	0.005449735	0.18834591	Cg_07_19	Cdc50-like protein

M896_070520	0.002270995	0.35276357	Cg_07_18	Sec1-like vacuolar protein sorting-associated protein
M896_070530	0.001707552	0.47090614	Cg_07_17	hypothetical protein
M896_070540	0.002530864	1.27007364	Cg_07_16	L-type amino acid transporter
M896_070550	0.002459491	Inf	Cg_07_15	hypothetical protein
M896_070560	0.000930579	Inf	Cg_07_13	putative hemolysin III-like integral membrane protein
M896_070570	0.000315956	Inf	Cg_07_12	CCR4-NOT transcriptional regulation complex protein
M896_070580	0.000724884	Inf	Cg_07_11	DNA polymerase sigma
M896_070590	0	Inf	Cg_07_10	nucleoside diphosphate kinase
M896_070600	0.002479402	2.1577381	Cg_07_9	hypothetical protein
M896_070610	0	Inf	Cg_07_8	hypothetical protein
M896_070630	0.001596424	0.05808544	Cg_07_6	ER lumen protein retaining receptor
M896_070650	0.002065527	1.38116498	Cg_07_5	Psp1-like protein
M896_070660	0.003255485	0.49434599	Cg_07_4	hypothetical protein
M896_070670	0.001147275	Inf	Cg_07_3	hypothetical protein
M896_070680	0.003161698	0.8451796	Cg_07_2	hypothetical protein
M896_080010	0.001312336	0.20528192	Cg_02C_4	hypothetical protein
M896_080020	0.002407407	0.72151899	Cg_02C_5	hypothetical protein
M896_080040	0.001409374	0.88767456	Cg_02C_7	NGG1p interacting factor 3-like protein
M896_080050	0	Inf	Cg_02C_8	acidic ribosomal protein P2
M896_080060	0.002469136	Inf	Cg_02C_9	hypothetical protein
M896_080070	0	Inf	Cg_02C_10	gene silencing histone chaperone
M896_080080	0.001134857	4.55032677	Cg_02C_11	hypothetical protein
M896_080090	0.002957907	0.17019296	Cg_02C_12	hypothetical protein
M896_080100	0	Inf	Cg_02C_13	histidine acid phosphatase

M896_080110	0	Inf	Cg_02C_14	regulatory subunit of ATP-dependent 26S proteasome
M896_080120	0.002952603	0.19525832	Cg_02C_15	hypothetical protein
M896_080130	0.001851852	0.41903208	Cg_02C_16	subunit of transcription initiation factor IIE
M896_080140	0.0023216	0.53054951	Cg_02C_17	hypothetical protein
M896_080160	0.003233392	0.05992848	Cg_02C_18	mitochondrial import inner membrane translocase
M896_080170	0.002496306	0.13157814	Cg_02C_19	hypothetical protein
M896_080180	0.004007739	0.68032609	Cg_02C_20	hypothetical protein
M896_080190	0.003261698	0.26571719	Cg_02C_21	hypothetical protein
M896_080200	0.003607085	0.52863215	Cg_02C_22	hypothetical protein
M896_080210	0.001948718	1.80330075	Cg_02C_23	hypothetical protein
M896_080220	0.000939913	0.24212984	Cg_02C_24	tRNA/rRNA cytosine-C5-methylase
M896_080230	0.001926469	Inf	Cg_02C_25	RING-finger domain-containing ubiquitin ligase
M896_080240	0.002013664	Inf	Cg_02C_26	U2 snRNP/pre-mRNA association factor
M896_080250	0.001160705	1.40622916	Cg_02C_27	separase
M896_080260	0.00257649	Inf	Cg_02C_28	hypothetical protein
M896_080270	0.000716846	0	Cg_02C_29	serine/threonine protein kinase
M896_080280	0.001834545	0.09873138	Cg_02C_30	hypothetical protein
M896_080290	0.001216779	Inf	Cg_02C_31	RNA exonuclease
M896_080300	0.002230545	Inf	Cg_02C_32	chromosome segregation ATPase
M896_080310	0.002614379	Inf	Cg_02C_33	hypothetical protein
M896_080320	0.000939746	Inf	Cg_02C_34	ubiquitin carboxyl-terminal hydrolase
M896_080330	0.001305404	0.96903232	Cg_02C_35	hypothetical protein
M896_080340	0.003641352	Inf	Cg_02C_36	hypothetical protein
M896_080350	0.000658793	0.40020436	Cg_02C_37	hypothetical protein

M896_080360	0.001947446	Inf	Cg_02C_38	hypothetical protein
M896_080370	0.002035002	Inf	Cg_02C_39	hypothetical protein
M896_080380	0.003277614	Inf	Cg_02C_40	ribosomal protein L7Ae
M896_080390	0.001653439	Inf	Cg_02C_41	hypothetical protein
M896_080400	0.004009877	2.45718808	Cg_02C_43	hypothetical protein
M896_080410	0.00167127	1.16243862	Cg_02C_44	Cdc46/Mcm ATPase
M896_080420	0.0016122	Inf	Cg_02C_45	hypothetical protein
M896_080430	0.002557078	Inf	Cg_02C_46	hypothetical protein
M896_080440	0.001355932	0.41096374	Cg_02C_47	serine/threonine kinase
M896_080500	0	Inf	Cg_08A_90	putative ATP binding protein
M896_080510	0.003149257	Inf	Cg_08A_89	hypothetical protein
M896_080520	0.003730445	1.51363337	Cg_08A_88	EPP-like transporter
M896_080530	0.00110257	0.46429405	Cg_08A_87	hypothetical protein
M896_080540	0.003132777	Inf	Cg_08A_86	hypothetical protein
M896_080550	0.000711446	Inf	Cg_08A_85	26S proteasome regulatory complex protein
M896_080560	0.001275046	Inf	Cg_08A_84	adrenodoxin-like ferredoxin
M896_080570	0.002214657	1.66872847	Cg_08A_83	Rad50-like protein
M896_080580	0.004540024	Inf	Cg_08A_82	hypothetical protein
M896_080590	0.00123832	0.40407865	Cg_08A_81	hypothetical protein
M896_080600	0.002323893	Inf	Cg_08A_80	hypothetical protein
M896_080610	0	Inf	Cg_08A_79	hypothetical protein
M896_080620	0.001814059	Inf	Cg_08A_78	small ubiquitin-related modifier protein
M896_080630	0	Inf	Cg_08A_77	hypothetical protein
M896_080650	0.001618234	0.46589201	Cg_08A_76	chromosome segregation ATPase
M896_080660	0.00151005	0.30558962	Cg_08A_75	hypothetical protein
M896_080670	0.001590072	Inf	Cg_08A_74	subunit Sec62 of preprotein translocase

M896_080680	0.000727107	Inf	Cg_08A_73	hypothetical protein
M896_080690	0	Inf	Cg_08A_72	hypothetical protein
M896_080700	0	Inf	Cg_08A_71	hypothetical protein
M896_080710	0.002213369	0.16519435	Cg_08A_70	aquaporin
M896_080720	0.003161002	0.40421218	Cg_08A_69	hypothetical protein
M896_080730	0.000759259	0	Cg_08A_68	DnaJ-class molecular chaperone
M896_080740	0.001967112	3.38499302	Cg_08A_67	nuclear pore protein
M896_080750	0.000841751	Inf	Cg_08A_66	hypothetical protein
M896_080760	0.002336029	1.68561691	Cg_08A_65	hypothetical protein
M896_080770	0.000383555	Inf	Cg_08A_64	glyceraldehyde-3-phosphate dehydrogenase
M896_080780	0.002146733	Inf	Cg_08A_63	hypothetical protein
M896_080790	0.001652649	0.19524102	Cg_08A_62	acidic ribosomal protein P0
M896_080800	0.002696388	0.51054665	Cg_08A_61	Ski2-like helicase
M896_080810	0.001638807	Inf	Cg_08A_60	subunit Rpb8 of RNA polymerase
M896_080820	0.001282271	0.85705884	Cg_08A_58	subunit Cullin of E3 ubiquitin ligase
M896_080840	0.000644122	Inf	Cg_08A_55	hypothetical protein
M896_080850	0.000789622	0	Cg_08A_54	RNA polymerase II transcription
M896_080870	0.004042162	1.30220027	Cg_08A_53	TFIIF-interacting CTD phosphatase
M896_080880	0.005152979	2.28452592	Cg_08A_52	alpha/beta hydrolase domain-containing protein
M896_080890	0.001783983	0.30654544	Cg_08A_51	hypothetical protein
M896_080900	0.003890054	0.17387625	Cg_08A_50	hypothetical protein
M896_080910	0.000694444	Inf	Cg_08A_49	ribosomal protein L24
M896_080920	0.005185185	Inf	Cg_08A_48	putative subunit RPA3 of replication protein A
M896_080930	0.000995619	Inf	Cg_08A_47	subunit Rpb5 of DNA-directed RNA polymerase

M896_080940	0.002080732	Inf	Cg_08A_46	hypothetical protein
M896_080950	0	Inf	Cg_08A_45	hypothetical protein
M896_080960	0.000911321	Inf	Cg_08A_44	subunit DPH2 of diphthamide synthase
M896_080970	0.002436647	Inf	Cg_08A_43	hypothetical protein
M896_080980	0.00119911	0.79093608	Cg_08A_42	hypothetical protein
M896_080990	0.002071563	0.09713989	Cg_08A_41	hypothetical protein
M896_081000	0.000766284	Inf	Cg_08A_40	ribosomal protein L37a
M896_081010	0.000813224	0.21683115	Cg_08A_38	subunit POB3 of nucleosome-binding factor SPN
M896_081020	0.00295584	0.23952794	Cg_08A_37	WD40 domain-containing protein
M896_081030	0.005954416	0.31016049	Cg_08A_36	hypothetical protein
M896_081040	0.003127572	0.07809415	Cg_08A_35	subunit alpha of proteasome
M896_081050	0.003295421	1.26949737	Cg_08A_34	NAD kinase
M896_081060	0.002049957	0.05422188	Cg_08A_33	ATP-dependent RNA helicase
M896_081070	0.002288152	Inf	Cg_08A_32	hypothetical protein
M896_081080	0.005499439	0.27342366	Cg_08A_31	hypothetical protein
M896_081090	0.003190883	0.33649658	Cg_08A_30	hypothetical protein
M896_081100	0.001129575	1.03594267	Cg_08A_29	ATP-dependent DNA helicase RecQ
M896_081110	0.001477261	0.52076719	Cg_08A_28	subunit 3 of splicing factor 3a
M896_081120	0.001763668	0	Cg_08A_27	hypothetical protein
M896_081140	0.003840036	0.57106291	Cg_08A_25	tRNA-dihydrouridine synthase
M896_081150	0.001967593	Inf	Cg_08A_24	hypothetical protein
M896_081160	0.002493583	1.13530046	Cg_08A_23	alpha tubulin
M896_081170	0.003112356	Inf	Cg_08A_22	hypothetical protein
M896_081180	0.001601602	Inf	Cg_08A_21	hypothetical protein
M896_081190	0.001773781	Inf	Cg_08A_20	formin homology 2 domain-containing protein

M896_081200	0.001030596	Inf	Cg_08A_19	hypothetical protein
M896_081210	0.000995025	Inf	Cg_08A_18	hypothetical protein
M896_081220	0.003404415	0.68472085	Cg_08A_17	hypothetical protein
M896_081230	0.000214362	0	Cg_08A_16	Golgi nucleoside diphosphatase
M896_081240	0.003805175	0.85314404	Cg_08A_15	serine/threonine kinase
M896_081250	0.001503928	0.46348312	Cg_08A_14	glucosamine-fructose-6-phosphate
M896_081260	0.001186468	0	Cg_08A_13	hypothetical protein
M896_081270	0.004032604	0.66763472	Cg_08A_12	Hsp90 ATPase activator
M896_081280	0	Inf	Cg_08A_11	hypothetical protein
M896_081290	0	Inf	Cg_08A_10	hypothetical protein
M896_081300	0.001753697	0.57668126	Cg_08A_9	subunit beta of translation initiation factor IF-2
M896_081310	0.002211564	0.42755769	Cg_08A_8	rRNA methylase
M896_081320	0.001010101	0	Cg_08A_7	putative V-type ATP synthase
M896_081330	0.00464135	Inf	Cg_08A_6	hypothetical protein
M896_081340	0.001457683	0.23546566	Cg_08A_5	hypothetical protein
M896_081350	0.002682998	Inf	Cg_08A_3	ubiquitin/L40 ribosomal protein fusion
M896_081360	0.001481481	Inf	Cg_08A_1	ribosomal protein L22/L17e
M896_081370	0.001363819	0.32418637	Cg_08B_1	beta type-1 subunit of proteasome
M896_081380	0.001359369	0.92737385	Cg_08B_2	hypothetical protein
M896_081390	0.001657414	0.21968222	Cg_08B_3	Dopey-like leucine zipper transcription factor
M896_081400	0.003898635	Inf	Cg_08B_4	hypothetical protein
M896_081410	0.007070707	0.1014245	Cg_08B_5	ribosomal protein L37
M896_081420	0.001346801	0	Cg_08B_6	hypothetical protein
M896_081440	0.000758956	Inf	Cg_08B_7	hypothetical protein
M896_081460	0.005677534	0.1843736	Cg_08B_8	hypothetical protein

M896_081480	0.003392378	2.90684076	Cg_08B_10	type V P-ATPase
M896_081490	0.001413862	Inf	Cg_08B_11	hypothetical protein
M896_081510	0.003383739	0.84448821	Cg_08B_16	hypothetical protein
M896_081530	0.003703704	0.27319154	Cg_08B_18	hypothetical protein
M896_081540	0.001881246	Inf	Cg_08B_19	threonyl-tRNA synthetase
M896_081550	0.002645503	Inf	Cg_08B_20	hypothetical protein
M896_081560	0.00313647	1.50436823	Cg_08B_21	hypothetical protein
M896_081570	0.000982415	Inf	Cg_08B_22	hypothetical protein
M896_081580	0.002523659	0.88228926	Cg_08B_23	tRNA-dihydrouridine synthase
M896_081590	0	Inf	Cg_08B_24	hypothetical protein
M896_081600	0.002333841	0.46860137	Cg_08B_25	mRNA decapping enzyme 2
M896_081610	0.000713735	0.19448884	Cg_08B_26	regulatory subunit 4 of proteosome
M896_081620	0.001332268	Inf	Cg_08B_27	hypothetical protein
M896_081630	0	Inf	Cg_08B_28	hypothetical protein
M896_081640	0.001158504	Inf	Cg_08B_29	subunit alpha of phenylalanyl-tRNA synthetase
M896_081650	0.001865502	1.94084043	Cg_08B_30	hypothetical protein
M896_081660	0.004640272	Inf	Cg_08B_31	putative acetyltransferase
M896_081670	0.002081937	0.49321424	Cg_08B_32	transcriptional accessory-like protein
M896_081680	0.003751804	Inf	Cg_08B_33	ribosomal protein S2
M896_081690	0.002364066	0.50369932	Cg_08B_35	hypothetical protein
M896_081700	0	Inf	Cg_08B_36	hypothetical protein
M896_081710	0.00158584	0.57493713	Cg_08B_37	hypothetical protein
M896_081720	0.003902439	Inf	Cg_08B_38	MADS domain-containing protein
M896_081730	0	Inf	Cg_08B_39	subunit 7 of RNA polymerase II
M896_090020	0	Inf	Cg_09A_3	hypothetical protein
M896_090030	0.001234568	Inf	Cg_09A_4	hypothetical protein

M896_090040	0.001336767	Inf	Cg_09A_5	ABCG White-like ABC transporter
M896_090050	0.002366522	1.05939732	Cg_09A_6	hypothetical protein
M896_090060	0.000801833	Inf	Cg_09A_7	hypothetical protein
M896_090070	0.002763958	Inf	Cg_09A_8	putative small nuclear ribonucleoprotein
M896_090080	0.002723312	Inf	Cg_09A_9	hypothetical protein
M896_090090	0.002508961	Inf	Cg_09A_10	hypothetical protein
M896_090100	0	Inf	Cg_09A_11	small subunit of clathrin adaptor complex
M896_090110	0	Inf	Cg_09A_12	EF-Hand Ca2+-binding protein
M896_090120	0.000985389	Inf	Cg_09A_13	hypothetical protein
M896_090130	0.001702509	1.3325867	Cg_09A_14	hypothetical protein
M896_090140	0.002066277	Inf	Cg_09A_15	hypothetical protein
M896_090150	0.00546566	0.24980288	Cg_09A_16	hypothetical protein
M896_090160	0.002326363	1.20022626	Cg_09A_17	hypothetical protein
M896_090180	0.00255144	Inf	Cg_09A_18	cyclin-dependent protein kinase
M896_090190	0.001675485	Inf	Cg_09A_19	GTP-binding protein
M896_090200	0.000513809	Inf	Cg_09A_20	subunit H of vacuolar H+-ATPase V1
M896_090210	0.00222484	0.66278091	Cg_09A_21	cyclin
M896_090220	0.001038961	0.16044191	Cg_09A_23	hypothetical protein
M896_090230	0.000676819	Inf	Cg_09A_24	beta type-2 subunit of proteasome
M896_090240	0.00138214	0.15561736	Cg_09A_25	minichromosome maintenance protein
M896_090250	0.000713558	0	Cg_09A_26	hypothetical protein
M896_090260	0.002808849	0.1522553	Cg_09A_28	hypothetical protein
M896_090270	0	Inf	Cg_09A_29	DNA-directed RNA polymerase
M896_090280	0.002777778	Inf	Cg_09A_30	subunit H of ATP synthase
M896_090290	0.003870968	0.1013777	Cg_09A_31	putative GTPase
M896_090300	0.001665781	0.51546696	Cg_09A_32	hypothetical protein

M896_090310	0.003328645	0.20255488	Cg_09A_33	ribosomal protein S15
M896_090320	0.002496195	0.17856007	Cg_09A_34	hypothetical protein
M896_090330	0.003627075	0	Cg_09A_35	ribosomal protein L26
M896_090340	0.000842549	0	Cg_09A_36	RNA binding protein
M896_090350	0.005282802	0.33974359	Cg_09A_37	histidine triad nucleotide-binding protein
M896_090360	0.000959954	0.04161079	Cg_09A_38	tRNA/rRNA cytosine-C5-methylase
M896_090370	0	Inf	Cg_09A_39	histone H2B-like protein
M896_090380	0.002696629	0.29970695	Cg_09A_40	hypothetical protein
M896_090390	0.004227181	1.84644178	Cg_09A_41	hypothetical protein
M896_090400	0	Inf	Cg_09A_42	hypothetical protein
M896_090410	0.001909126	Inf	Cg_09A_43	hypothetical protein
M896_090420	0.00162174	Inf	Cg_09A_44	E1 ubiquitin activating enzyme-like protein
M896_090430	0.00462963	0.26001284	Cg_09A_45	peptidyl-prolyl cis-trans isomerase
M896_090440	0.000903342	Inf	Cg_09A_46	hypothetical protein
M896_090450	0.000589817	Inf	Cg_09A_47	cysteinyl-tRNA-synthetase
M896_090460	0	Inf	Cg_09A_48	hypothetical protein
M896_090470	0.0022285	0.12843549	Cg_09A_49	hypothetical protein
M896_090480	0.00177703	0.81416054	Cg_09A_50	RNA binding domain-containing protein
M896_090490	0.006475183	0.6557543	Cg_09A_51	hypothetical protein
M896_090500	0	Inf	Cg_09A_52	Sm-like protein
M896_090510	0.000980186	0.47891508	Cg_09A_53	arginyl-tRNA synthetase
M896_090520	0.000877533	Inf	Cg_09A_54	Zn-finger domain-containing protein
M896_090560	0.0019696	0	Cg_09A_55	hypothetical protein
M896_090570	0.002837153	0.38797907	Cg_09A_56	hypothetical protein
M896_090580	0.002955083	0.03677705	Cg_09A_57	ribosomal protein L18

M896_090590	0.002185792	0.268133	Cg_09A_58	chromosome condensation complex Condensin
M896_090600	0.002896407	0.43263151	Cg_09A_59	hypothetical protein
M896_090610	0.001936919	0.53058292	Cg_09A_60	catalytic subunit of DNA primase
M896_090620	0.001976977	0.66432036	Cg_09A_62	hypothetical protein
M896_090630	0.004063492	0.87872966	Cg_09A_63	subunit E' of DNA-directed RNA polymerase
M896_090640	0.000732601	Inf	Cg_09A_65	hypothetical protein
M896_090660	0.002010582	0.7438291	Cg_09A_66	gamma-tubulin
M896_090670	0.001003584	0	Cg_09A_67	subunit zeta of vesicle coat complex COPI
M896_090680	0.000647501	Inf	Cg_09A_68	hypothetical protein
M896_090690	0	Inf	Cg_09A_69	Golgi-to-ER retrieval protein
M896_090700	0.002629544	0.44305273	Cg_09A_70	hypothetical protein
M896_090710	0.002942097	0.32092215	Cg_09A_71	hypothetical protein
M896_090720	0.000614086	Inf	Cg_09A_73	Rab5-like GTPase
M896_090730	0.002988954	Inf	Cg_09A_74	MRP-like protein
M896_090740	0.003144455	0.66873447	Cg_09A_75	ERCC4-type nuclease
M896_090750	0.004368471	Inf	Cg_09A_76	subunit Rpb4 of RNA polymerase II
M896_090760	0.001678657	Inf	Cg_09A_77	8-oxoguanine DNA glycosylase
M896_090770	0.003113733	7.13009756	Cg_09A_78	hypothetical protein
M896_090780	0.005023414	0.93991313	Cg_09A_79	hypothetical protein
M896_090790	0.000925926	Inf	Cg_09A_80	signal recognition particle protein Srp19
M896_090800	0.002382194	Inf	Cg_09A_81	hypothetical protein
M896_090810	0.000787037	0	Cg_09A_82	ribosomal protein L4
M896_090820	0.001880262	0.87688507	Cg_09A_83	hypothetical protein
M896_090830	0.000945626	Inf	Cg_09A_84	subunit of mRNA deadenylase
M896_090840	0.001346801	Inf	Cg_09A_85	ubiquitin-conjugating enzyme E2

M896_090850	0.000713967	Inf	Cg_09A_86	ribosomal protein S4
M896_090860	0.003198653	0.51523968	Cg_09A_87	endonuclease III
M896_090870	0.001434943	Inf	Cg_09A_88	kinesin-like protein
M896_090880	0.002103338	Inf	Cg_09A_90	translation initiation factor 4E
M896_090890	0.000690989	0.15270822	Cg_09A_91	hypothetical protein
M896_090900	0.000493827	0	Cg_09A_92	hypothetical protein
M896_090910	0	Inf	Cg_09A_93	cyclic nucleotide-binding domain-containing protein
M896_090940	0.001559454	Inf	Cg_09A_94	hypothetical protein
M896_090950	0.000894479	Inf	Cg_09A_95	hypothetical protein
M896_090960	0.000252525	0	Cg_09A_96	heat shock transcription factor
M896_090970	0.002668071	Inf	Cg_09A_97	hypothetical protein
M896_090990	0.000958056	Inf	Cg_09A_98	hypothetical protein
M896_091000	0.002277396	Inf	Cg_09A_99	hypothetical protein
M896_091010	0.002032908	0.25277391	Cg_09A_100	hypothetical protein
M896_091020	0.003703704	0.11340051	Cg_09A_101	hypothetical protein
M896_091030	0.000868455	Inf	Cg_09A_102	hypothetical protein
M896_091050	0.000890313	Inf	Cg_09A_107	hypothetical protein
M896_091060	0.001217656	Inf	Cg_09A_106	ribosomal protein S13
M896_091080	0.001699083	0.10968764	Cg_09B_2	ATP-dependent RNA helicase
M896_091090	0.001081081	0.29887445	Cg_09B_3	histone acetyltransferase
M896_091100	0.003265048	0.40378022	Cg_09B_4	subunit beta of coatomer complex
M896_091110	0.000199005	0	Cg_09B_5	WD40 domain-containing protein
M896_091120	0.001017812	0.15043773	Cg_09B_6	Rad3-like DNA helicase
M896_091130	0.004232804	0.16168627	Cg_09B_7	hypothetical protein
M896_091140	0.003776325	0.13399148	Cg_09B_8	hypothetical protein

M896_091150	0.000429415	Inf	Cg_09B_9	putative short-chain alcohol dehydrogenase
M896_091160	0.003135983	Inf	Cg_09B_10	ribosomal protein L23
M896_091170	0.002962963	Inf	Cg_09B_11	hypothetical protein
M896_091180	0.003734313	Inf	Cg_09B_12	hypothetical protein
M896_091190	0.003105481	0.72298875	Cg_09B_13	hypothetical protein
M896_091200	0.002684336	0.49267902	Cg_09B_14	putative subunit p30 of RNase P/RNase MRP
M896_091210	0.007611281	0.47312754	Cg_09B_15	putative subunit p30 of RNase P/RNase MRP
M896_091220	0.002783675	0.07976026	Cg_09B_16	hypothetical protein
M896_091230	0.001693122	0	Cg_09B_17	hypothetical protein
M896_091250	0.002796847	0.89116303	Cg_09B_18	hypothetical protein
M896_091260	0.001634738	0.07569346	Cg_09B_19	hypothetical protein
M896_091270	0.002260235	0.43842744	Cg_09B_20	WD40 domain-containing protein
M896_091280	0.002759206	1.10830517	Cg_09B_21	hypothetical protein
M896_091290	0.001877359	0	Cg_09B_22	developmentally regulated GTP-binding protein
M896_091300	0.001346801	0.35169965	Cg_09B_23	hypothetical protein
M896_091310	0.003574368	0.03211918	Cg_09B_24	subunit beta of prenyltransferase
M896_091320	0.001221293	0.08338103	Cg_09B_25	ATP/ADP translocase
M896_091330	0.002855868	0.2253937	Cg_09B_26	hypothetical protein
M896_091340	0.001209373	0	Cg_09B_27	subunit M of DNA-directed RNA polymerase
M896_091350	0.002145215	0.16836989	Cg_09B_28	hypothetical protein
M896_091360	0.002494591	0.26797386	Cg_09B_29	TRAM protein transporter
M896_091370	0.009710551	0.90605229	Cg_09B_30	glutaredoxin
M896_091380	0.004983786	0.49764023	Cg_09B_31	GATA Zn-finger-containing transcription
M896_091390	0.000794786	Inf	Cg_09B_32	hypothetical protein
M896_091400	0.00144966	2.05406004	Cg_09B_33	phosphoinositide polyphosphatase

M896_091410	0.002798354	0.10285714	Cg_09B_34	hypothetical protein
M896_091420	0.001263962	Inf	Cg_09B_35	hypothetical protein
M896_091430	0.003143275	0.34453842	Cg_09B_36	hypothetical protein
M896_091440	0.002901519	0.16345535	Cg_09B_37	N2%2CN2-dimethylguanosine tRNA methyltransferase
M896_091450	0.00682121	0.41007874	Cg_09B_38	hypothetical protein
M896_091460	0.004088615	2.69421615	Cg_09B_39	serine/threonine kinase
M896_091470	0.004265448	0.42477208	Cg_09B_40	hypothetical protein
M896_091480	0.001698947	0.30365449	Cg_09B_41	hypothetical protein
M896_091490	0.003161123	0.11773733	Cg_09B_42	ribosome biogenesis regulatory protein
M896_091500	0.002890696	0.41843734	Cg_09B_43	vacuolar-type H+-ATPase
M896_091510	0.003312073	0.12414398	Cg_09B_44	putative pseudouridylate synthase
M896_091520	0.001146953	0	Cg_09B_45	hypothetical protein
M896_091530	0.005331089	0.69191686	Cg_09B_47	hypothetical protein
M896_091540	0.001427115	0	Cg_09B_48	ribosomal protein L10
M896_091550	0.000949668	0	Cg_09B_49	subunit alpha of type-2 proteasome
M896_091560	0.002539182	0.61781241	Cg_09B_50	hypothetical protein
M896_091570	0.000223199	Inf	Cg_09B_51	hypothetical protein
M896_091580	0.002579365	1.91488438	Cg_09B_52	putative subunit of exosome
M896_091590	0.002907208	0	Cg_09B_53	serine/threonine kinase
M896_091600	0.002026555	0.32655215	Cg_09B_54	cleavage and polyadenylation specificity factor
M896_091610	0.000496475	Inf	Cg_09B_55	chromatin remodeling bromodomain-containing protein
M896_091620	0.006251338	0.88606341	Cg_09B_56	hypothetical protein
M896_091630	0	Inf	Cg_09B_57	transcription elongation factor SPT4
M896_091640	0.001413627	Inf	Cg_09B_58	hypothetical protein

M896_091650	0.002578068	0.10279322	Cg_09B_59	hypothetical protein
M896_091660	0.00213791	0.46938776	Cg_09B_60	GTPase-activating protein
M896_091670	0.001957242	0.08140234	Cg_09B_61	hypothetical protein
M896_091680	0.001680486	Inf	Cg_09B_62	hypothetical protein
M896_091690	0.001977066	0.18810166	Cg_09B_64	hypothetical protein
M896_091700	0.002279202	0.1208802	Cg_09B_65	subunit of RNA polymerase III transcription factor IIIC
M896_091710	0.004008715	0.10681147	Cg_09B_66	ribosomal protein L14E/L6E/L27E
M896_091720	0.003691718	0.26964631	Cg_09B_67	cyclin-dependent protein kinase
M896_091730	0.002234994	Inf	Cg_09B_69	hypothetical protein
M896_091740	0.001953602	Inf	Cg_09B_70	hypothetical protein
M896_091750	0.001335241	Inf	Cg_09B_71	putative PP-loop ATPase
M896_091760	0.00085676	Inf	Cg_09B_72	glucose-6-phosphate 1-dehydrogenase
M896_091780	0.001298929	Inf	Cg_09B_73	subunit beta of type-5 proteasome
M896_091790	0.001257862	0.68961614	Cg_09B_74	hypothetical protein
M896_091800	0.001057348	0.75541744	Cg_09B_77	AAA ATPase
M896_091830	0.000246002	Inf	Cg_09C_1	cyclin-dependent protein kinase
M896_091840	0.002237654	Inf	Cg_09C_3	ADP-ribosylation factor 1
M896_091850	0.002095832	1.05904625	Cg_09C_5	hypothetical protein
M896_091860	0.00079905	Inf	Cg_09C_6	cyclin-dependent protein kinase
M896_091880	0.001414359	1.60717045	Cg_09C_8	hypothetical protein
M896_091890	0.001030169	0	Cg_09C_9	hypothetical protein
M896_091900	0.000750117	Inf	Cg_09C_10	ribosomal protein L12
M896_091910	0.001224612	0.40450508	Cg_09C_11	hypothetical protein
M896_091920	0.001040366	Inf	Cg_09C_12	hypothetical protein
M896_091930	0.003174603	0.23098395	Cg_09C_13	hypothetical protein

M896_091940	0.002527233	Inf	Cg_09C_14	hypothetical protein
M896_100020	0.000151172	Inf	Cg_10_6	hypothetical protein
M896_100030	0.003876774	1.46447334	Cg_10_7	hypothetical protein
M896_100040	0.001221293	Inf	Cg_10_8	hypothetical protein
M896_100050	0	Inf	Cg_10_9	hypothetical protein
M896_100060	0.002082948	2.25408652	Cg_10_10	hypothetical protein
M896_100070	0.006045126	0.55493063	Cg_10_11	RING-finger-containing E3 ubiquitin ligase
M896_100080	0	Inf	Cg_10_12	hypothetical protein
M896_100090	0.000972101	0.23723615	Cg_10_13	pyruvate kinase
M896_100100	0.001210361	Inf	Cg_10_14	hypothetical protein
M896_100110	0.000755124	Inf	Cg_10_15	hypothetical protein
M896_100120	0.001881624	Inf	Cg_10_16	histone deacetylase
M896_100130	0.003886777	0.22856188	Cg_10_17	hypothetical protein
M896_100140	0.000973236	0.2543235	Cg_10_18	hypothetical protein
M896_100150	0.001556178	0.49456522	Cg_10_19	Prp40-like splicing factor
M896_100160	0.001637427	0.24844857	Cg_10_20	hypothetical protein
M896_100170	0.000751476	Inf	Cg_10_21	beta type-7 subunit of proteasome
M896_100180	0.004557823	0.33592538	Cg_10_22	hypothetical protein
M896_100190	0.001863499	0.08310029	Cg_10_23	hypothetical protein
M896_100200	0.002867384	0.23287671	Cg_10_24	hypothetical protein
M896_100210	0.000801716	0	Cg_10_25	5'-3' exonuclease
M896_100220	0.004489867	0.36954529	Cg_10_26	subunit B of DNA polymerase delta
M896_100230	0.005041152	0.25003696	Cg_10_28	hypothetical protein
M896_100240	0.003966689	0.68065309	Cg_10_29	hypothetical protein
M896_100250	0	Inf	Cg_10_30	hypothetical protein
M896_100260	0.002751654	0.7332372	Cg_10_31	hypothetical protein

M896_100270	0.00152587	0.16806278	Cg_10_32	septin
M896_100280	0.002540705	0.2681189	Cg_10_33	ribonuclease
M896_100290	0.001607268	Inf	Cg_10_34	hypothetical protein
M896_100300	0	Inf	Cg_10_35	hypothetical protein
M896_100310	0.006643757	0.05771608	Cg_10_36	hypothetical protein
M896_100320	0.001678657	0.10004997	Cg_10_37	hypothetical protein
M896_100330	0.003059244	0.20875902	Cg_10_38	hypothetical protein
M896_100340	0.001647319	0.16701089	Cg_10_39	hypothetical protein
M896_100350	0.003284072	1.04529593	Cg_10_40	hypothetical protein
M896_100360	0.001893004	0.12665536	Cg_10_41	hypothetical protein
M896_100370	0.001008109	0.32444896	Cg_10_42	deoxyhypusine synthase
M896_100380	0.001723356	0.35443643	Cg_10_43	23S rRNA methylase
M896_100390	0.002405002	0	Cg_10_44	Yos1-like protein
M896_100400	0.002673893	0	Cg_10_45	Rad52 recombination DNA repair protein
M896_100410	0.002165909	0.27717842	Cg_10_46	ER to Golgi transport membrane protein
M896_100420	0.002043423	Inf	Cg_10_47	hypothetical protein
M896_100430	0.00497385	0.25464019	Cg_10_48	hypothetical protein
M896_100440	0.003849903	0.17264032	Cg_10_49	hypothetical protein
M896_100450	0.003543743	0.08346759	Cg_10_50	PP2A-like phosphoprotein phosphatase
M896_100460	0.000848176	0.59684721	Cg_10_51	hypothetical protein
M896_100480	0.000749064	Inf	Cg_10_53	hypothetical protein
M896_100490	0.001569366	Inf	Cg_10_54	hypothetical protein
M896_100500	0	Inf	Cg_10_55	cyclin
M896_100510	0.002222222	0.08793201	Cg_10_56	subunit of pyruvate dehydrogenase E1
M896_100520	0.001602772	Inf	Cg_10_57	hypothetical protein
M896_100530	0.002251271	0.41674717	Cg_10_58	hypothetical protein

M896_100540	0.00308642	Inf	Cg_10_59	glycosylphosphatidylinositol transamidase
M896_100550	0.003251215	Inf	Cg_10_60	hypothetical protein
M896_100560	0	Inf	Cg_10_61	hypothetical protein
M896_100570	0	Inf	Cg_10_62	hypothetical protein
M896_100580	0.001224612	0.37284961	Cg_10_63	hypothetical protein
M896_100590	0.000973545	Inf	Cg_10_64	hypothetical protein
M896_100600	0.00063234	Inf	Cg_10_65	hypothetical protein
M896_110010	0	Inf	Cg_11A_16	hypothetical protein
M896_110020	0.004433078	2.41659755	Cg_11A_15	Ndc80p-complex mitotic spindle protein
M896_110030	0.002984279	0.78466503	Cg_11A_14	asparaginyl-tRNA synthetase
M896_110040	0.002615999	0.77969599	Cg_11A_13	hypothetical protein
M896_110050	0.001054614	0.81537753	Cg_11A_11	hypothetical protein
M896_110060	0.001827291	0.47972693	Cg_11A_9	autoantigen NGP-1
M896_110070	0.00154321	0.65607164	Cg_11A_8	ATP-dependent RNA helicase
M896_110080	0.000831444	0.08908026	Cg_11A_7	hypothetical protein
M896_110090	0.003239875	2.05916447	Cg_11A_6	DNA repair flap endonuclease
M896_110100	0.002636535	Inf	Cg_11A_5	subunit Tfb5 of transcription factor TFIIH complex
M896_110110	0.002112933	Inf	Cg_11A_4	translation factor
M896_110120	0.001440329	Inf	Cg_11A_3	ribosomal biogenesis protein
M896_110130	0.002297485	Inf	Cg_11A_18	Yip1-interacting factor
M896_110140	0.001222987	Inf	Cg_11B_1	vacuole import and degradation protein
M896_110150	0.000428528	Inf	Cg_11B_2	NADPH cytochrome p450 reductase
M896_110160	0.002065336	2.5090022	Cg_11B_3	hypothetical protein
M896_110170	0	Inf	Cg_11B_4	Cdc73-like RNA polymerase II accessory factor
M896_110180	0.001429767	1.14916899	Cg_11B_5	hypothetical protein

M896_110190	0.003727806	0.48244477	Cg_11B_6	putative methyl transferase
M896_110200	0.005282332	0.67897912	Cg_11B_8	hypothetical protein
M896_110210	0.001685824	Inf	Cg_11B_9	hypothetical protein
M896_110230	0.001756614	0.06719054	Cg_11B_10	subunit I of vacuolar-type H+-ATPase
M896_110240	0.00143636	0.19131139	Cg_11B_11	DNA helicase
M896_110250	0.006304177	0.17512438	Cg_11B_12	small nuclear ribonucleoprotein
M896_110260	0.002046647	0.40130043	Cg_11B_13	hypothetical protein
M896_110270	0.001010101	0	Cg_11B_14	hypothetical protein
M896_110280	0.002079272	0.39285505	Cg_11B_15	hypothetical protein
M896_110290	0.00039784	0	Cg_11B_16	regulatory subunit of ATP-dependent 26S proteasome
M896_110310	0.002124834	Inf	Cg_11B_17	PHD zinc finger domain-containing protein
M896_110320	0.001761606	0.67066715	Cg_11B_18	subunit IKAP of IkappaB kinase complex
M896_110330	0.000991543	0.65651103	Cg_11B_19	undecaprenyl pyrophosphate synthase
M896_110340	0.002353182	Inf	Cg_11B_20	hypothetical protein
M896_110350	0.001833517	Inf	Cg_11B_21	DNA/RNA helicase
M896_110360	0.00080558	Inf	Cg_11B_22	hypothetical protein
M896_110370	0.000478412	Inf	Cg_11B_23	structural maintenance of chromosomes protein
M896_110380	0.002979066	0.19538984	Cg_11B_24	hypothetical protein
M896_110390	0.000986414	Inf	Cg_11B_25	hypothetical protein
M896_110400	0.000766284	0	Cg_11B_26	hypothetical protein
M896_110410	0.001045752	Inf	Cg_11B_27	membrane traffic protein
M896_110420	0.002763958	Inf	Cg_11B_28	deoxycytidylate deaminase
M896_110430	0.00082811	1.40684713	Cg_11B_29	myosin heavy chain
M896_110440	0.001040366	Inf	Cg_11B_30	hypothetical protein

M896_110450	0.004702628	1.29836863	Cg_11B_31	hypothetical protein
M896_110460	0.002259492	Inf	Cg_11B_32	hypothetical protein
M896_120010	0	Inf	Cg_12A_5	hypothetical protein
M896_120020	0.000291918	0	Cg_12A_6	protein kinase domain-containing protein
M896_120040	0.006418002	0.16363744	Cg_12A_8	putative transcription regulator protein
M896_120050	0.000569801	Inf	Cg_12A_9	hypothetical protein
M896_120070	0.000733753	Inf	Cg_12A_11	putative CDK-activating kinase assembly factor
M896_120080	0	Inf	Cg_12A_12	ribosomal protein S21e
M896_120090	0.002083333	0.52362771	Cg_12A_13	triosephosphate isomerase
M896_120100	0.002319783	0.06642467	Cg_12A_14	putative exportin 1
M896_120110	0	Inf	Cg_12A_15	dephospho-CoA kinase
M896_120120	0.004047346	0.15987362	Cg_12A_16	hypothetical protein
M896_120130	0.001686835	0.04663905	Cg_12A_17	hypothetical protein
M896_120150	0.005228758	0.8348153	Cg_12A_19	hypothetical protein
M896_120160	0.002815485	0.7708544	Cg_12A_20	zinc finger domain-containing protein
M896_120170	0.001468891	0.45053129	Cg_12A_21	putative RAB escort protein
M896_120180	0.004018913	0.23761875	Cg_12A_22	putative Rad5p-binding protein
M896_120200	0.005144033	Inf	Cg_12A_24	hypothetical protein
M896_120220	0.000745551	0.49759641	Cg_12A_25	subunit SEC6 of exocyst complex
M896_120230	0.000984529	Inf	Cg_12A_26	putative transmembrane adaptor Erv26
M896_120240	0.000444444	0	Cg_12A_27	hypothetical protein
M896_120260	0.001704051	Inf	Cg_12A_28	hypothetical protein
M896_120270	0.002469136	0.1158648	Cg_12A_29	Sel1 repeat domain-containing protein
M896_120280	0.001786183	Inf	Cg_12A_30	RNP domain-containing protein
M896_120290	0.002974303	0.20565626	Cg_12A_31	phosphoinositide 4-kinase
M896_120300	0.003631436	0.41680365	Cg_12A_32	ribosomal biogenesis protein

M896_120310	0.001624021	0.80446387	Cg_12A_33	kinesin motor domain-containing protein
M896_120320	0.001844968	0.44014178	Cg_12A_34	GATase1 CTP synthase
M896_120330	0.004994071	0.44175555	Cg_12A_35	hypothetical protein
M896_120340	0.004257131	0.08260292	Cg_12A_36	RNP domain-containing protein
M896_120360	0.001562744	Inf	Cg_12A_37	polysaccharide deacetylase domain-containing protein
M896_120370	0.002757017	Inf	Cg_12A_38	hypothetical protein
M896_120380	0.004112474	Inf	Cg_12A_39	hypothetical protein
M896_120390	0.001387614	Inf	Cg_12A_41	aminoacyl-tRNA ligase
M896_120400	0.003124116	0.72665374	Cg_12A_42	Hsp70-like protein
M896_120410	0.001968635	1.33885438	Cg_12A_43	putative Arf GTPase activating protein
M896_120420	0.005913477	0.39349407	Cg_12A_44	hypothetical protein
M896_120430	0.001971756	Inf	Cg_12A_45	regulatory subunit of proteasome
M896_120440	0.005506391	Inf	Cg_12A_47	hypothetical protein
M896_120450	0.002258356	0	Cg_12A_48	subunit NuA4 of histone acetyltransferase
M896_120460	0.000673401	0	Cg_12A_49	subunit AC19 of RNA polymerase
M896_120470	0.003416797	0.24607309	Cg_12A_50	hypothetical protein
M896_120480	0.001123196	4.13668242	Cg_12A_51	pre-mRNA cleavage and polyadenylation
M896_120490	0.003003003	0.25589289	Cg_12A_52	CDP-alcohol phosphatidyltransferase
M896_120500	0.003348943	0.242397	Cg_12A_53	hypothetical protein
M896_120510	0.002469136	Inf	Cg_12A_54	hypothetical protein
M896_120520	0.006046863	0.96079545	Cg_12A_55	inositol metabolism VAMP-associated protein
M896_120530	0.001395231	Inf	Cg_12A_56	PP1 serine/threonine phosphatase
M896_120540	0.001557632	0	Cg_12A_57	eukaryotic translation initiation factor eIF2A
M896_120550	0.002386279	0.86104317	Cg_12A_58	hypothetical protein
M896_120560	0.00244606	Inf	Cg_12A_59	GDP-mannose pyrophosphorylase

M896_120570	0.003071364	Inf	Cg_12A_60	hypothetical protein
M896_120580	0.000629882	Inf	Cg_12A_61	hypothetical protein
M896_120590	0.002209227	0.09866137	Cg_12A_62	ribosomal protein S10
M896_120600	0.00182716	Inf	Cg_12A_63	class 2 transcription repressor NC2 beta
M896_120610	0.002962963	0.23691099	Cg_12A_64	hypothetical protein
M896_120620	0.000364299	Inf	Cg_12A_65	putative RNA-binding protein
M896_120630	0.002992677	0.52372251	Cg_12A_66	putative exonuclease
M896_120650	0.002262922	3.76283847	Cg_12A_67	putative mRNA deadenylase
M896_120660	0.001583974	Inf	Cg_12A_68	hypothetical protein
M896_120670	0.001654846	0.57960868	Cg_12A_70	Sec23-like protein transport protein
M896_120680	0.002333042	1.82391865	Cg_12A_72	minichromosome maintenance protein
M896_120690	0.001493429	Inf	Cg_12A_73	putative ribosomal protein S8
M896_120700	0.001705948	0.11396287	Cg_12A_74	DNA repair protein Rad51
M896_120710	0.004122991	0.04801753	Cg_12A_75	subunit of transcription initiation factor TFIID
M896_120720	0.002409278	0.09895002	Cg_12A_76	AAA family ATPase
M896_120730	0.001572062	0.18274818	Cg_12A_77	HrpA-like helicase
M896_120750	0.003210641	0.30641974	Cg_12A_78	putative ethanolamine-phosphate
M896_120770	0.001679747	0.10527888	Cg_12A_79	methionyl-tRNA synthetase
M896_120780	0.003033276	0.26292482	Cg_12A_80	subunit of U2 snRNP spliceosome
M896_120790	0.003183886	0.42799172	Cg_12A_81	hypothetical protein
M896_120800	0.001251251	Inf	Cg_12A_82	hypothetical protein
M896_120810	0.003569512	0.62756968	Cg_12A_83	subunit alpha of translation initiation factor 2
M896_120820	0.002893519	0	Cg_12A_84	hypothetical protein
M896_120830	0.002353756	Inf	Cg_12A_85	HS6-type ribosomal protein
M896_120840	0.000828801	Inf	Cg_12A_86	hypothetical protein
M896_120850	0.000723851	Inf	Cg_12A_87	serine/threonine kinase

M896_120860	0.001493931	0	Cg_12A_88	hypothetical protein
M896_120870	0.007446394	0.84294719	Cg_12A_89	putative synaptobrevin/VAMP-like protein
M896_120880	0.004109589	0.06348253	Cg_12A_90	hypothetical protein
M896_120890	0.004270153	0.25406504	Cg_12A_91	hypothetical protein
M896_120900	0.003145611	0.47838328	Cg_12A_92	hypothetical protein
M896_120910	0.004890918	0.30091503	Cg_12A_93	hypothetical protein
M896_120920	0.002276508	0.06716124	Cg_12A_94	putative AAA+ class ATPase
M896_120930	0.002420721	Inf	Cg_12A_95	hypothetical protein
M896_120940	0.003217841	0.57091179	Cg_12A_96	putative proton-dependent oligopeptide transport protein
M896_120950	0.002816505	0.27669903	Cg_12A_97	hypothetical protein
M896_120960	0.00177884	0.28380827	Cg_12A_98	hypothetical protein
M896_120970	0.00215911	0	Cg_12A_100	superoxide dismutase
M896_120980	0.001730703	0.42217082	Cg_12A_101	hypothetical protein
M896_120990	0.002521823	0.12044006	Cg_12A_102	isoleucyl-tRNA synthetase
M896_121000	0.002298851	0.81953187	Cg_12A_103	hypothetical protein
M896_121010	0.002625272	0.41758265	Cg_12A_104	translation elongation factor 2
M896_121020	0.002962963	0.422323	Cg_12A_105	hypothetical protein
M896_121030	0.004086845	Inf	Cg_12A_106	putative membrane protein
M896_121040	0.000356779	0	Cg_12A_107	hypothetical protein
M896_121050	0.005023205	0.50447079	Cg_12A_108	hypothetical protein
M896_121060	0.002102102	0.39762686	Cg_12A_109	putative nucleotide binding protein
M896_121070	0.004375951	0.10746248	Cg_12A_110	putative mitochondrial ABC transporter
M896_121080	0.001585243	0.31481744	Cg_12A_112	hypothetical protein
M896_121090	0.001310541	0.34477555	Cg_12A_113	hypothetical protein
M896_121100	0.002384359	0.27986106	Cg_12A_114	hypothetical protein

M896_121110	0.001579521	0.31348499	Cg_12A_115	hypothetical protein
M896_121120	0	Inf	Cg_12A_116	hypothetical protein
M896_121140	0.001624538	0.7388912	Cg_12A_118	putative DNA mismatch repair enzyme
M896_121150	0.001700381	Inf	Cg_12A_119	DNA helicase
M896_121160	0.001732804	3.57369103	Cg_12A_120	catalytic subunit A of V/A-type ATP synthase
M896_121170	0.002845385	1.25011929	Cg_12A_121	cyclin-dependent protein kinase
M896_121180	0.00462963	0.30255005	Cg_12A_122	hypothetical protein
M896_121190	0.002992258	1.97115724	Cg_12A_123	hypothetical protein
M896_121200	0.002046784	0.26315789	Cg_12A_124	transport protein particle
M896_121210	0.003948696	0.45369097	Cg_12A_125	putative transcription factor
M896_121220	0.010525879	5.2402664	Cg_12A_131	putative ABC-like lipid transport protein
M896_121240	0.001920439	Inf	Cg_12A_134	hypothetical protein
M896_121250	0.001304513	0.08884113	Cg_12A_135	putative negative regulator of transcription
M896_121260	0.004615671	0.06037736	Cg_12A_136	ribosomal protein L15
M896_121270	0	Inf	Cg_12A_137	subunit of polyadenylation factor I complex
M896_121280	0.001388889	Inf	Cg_12A_138	putative DNA-directed RNA polymerase
M896_121290	0.00246085	0.70921797	Cg_12A_139	hypothetical protein
M896_121300	0.001997009	0.25251005	Cg_12A_141	ATP binding subunit of chaperone protease
M896_121310	0.004008715	Inf	Cg_12A_142	hypothetical protein
M896_121320	0.000968661	0.10362135	Cg_12A_143	diphthamide biosynthesis methyltransferase
M896_121330	0.00177492	0.18395178	Cg_12A_144	hypothetical protein
M896_121340	0.002069717	0.41699401	Cg_12A_146	hypothetical protein
M896_121350	0.001385621	0.20084469	Cg_12A_147	translation elongation factor
M896_121360	0.001991239	Inf	Cg_12A_148	subunit of class 2 transcription repressor NC2
M896_121370	0.001596424	0.47464941	Cg_12A_149	hypothetical protein
M896_121380	0.001315671	0.93193639	Cg_12A_150	hypothetical protein

M896_121390	0.002042484	0.55665796	Cg_12A_151	NIMA-like serine/threonine kinase
M896_121400	0.004264871	0.09979425	Cg_12A_152	putative inorganic ion transport protein
M896_121410	0.004585538	0.24221886	Cg_12A_153	putative S-adenosylmethionine-dependent
M896_121420	0.002516619	0.38375463	Cg_12A_154	hypothetical protein
M896_121430	0.003174603	0.21483627	Cg_12A_155	putative endonuclease
M896_121440	0.006325427	4.3977938	Cg_12A_156	subunit of t-SNARE complex
M896_121450	0.004196104	0.30272782	Cg_12A_157	hypothetical protein
M896_121460	0.000410142	Inf	Cg_12A_158	ATPase domain 1 of RNase L inhibitor
M896_121470	0.00329554	Inf	Cg_12A_159	putative spindle pole body associated protein
M896_121480	0.002030178	Inf	Cg_12A_160	hypothetical protein
M896_121490	0.001583468	Inf	Cg_12A_161	tRNA pseudouridine synthase domain-containing protein
M896_121500	0.001196581	Inf	Cg_12A_162	ribosomal protein S19
M896_121510	0.003740374	0.4699409	Cg_12A_163	hypothetical protein
M896_121520	0.002116402	Inf	Cg_12A_164	inorganic phosphate transport protein
M896_121530	0.001506591	Inf	Cg_12A_165	hypothetical protein
M896_121540	0.001646091	Inf	Cg_12A_166	transcriptional repressor
M896_121550	0.002048131	Inf	Cg_12A_167	putative proteasome
M896_121560	0.003423592	Inf	Cg_12A_169	hypothetical protein
M896_121570	0.000614086	Inf	Cg_12A_170	hypothetical protein
M896_121580	0.000798838	0.67485667	Cg_12A_171	putative GTPase-activating protein
M896_121590	0.003866504	Inf	Cg_12A_172	hypothetical protein
M896_121600	0.003047619	0.33211168	Cg_12A_173	hypothetical protein
M896_121610	0.002827255	Inf	Cg_12A_174	hypothetical protein
M896_121620	0.001666667	0.82879954	Cg_12A_175	RNA binding repeat domain-containing protein

M896_121630	0.000932923	Inf	Cg_12A_176	putative amino acid permease
M896_121640	0.000814147	0	Cg_12A_177	hypothetical protein
M896_121650	0.00179937	Inf	Cg_12A_178	importin beta binding domain-containing protein
M896_121660	0.002126879	0.90078979	Cg_12A_179	cysteine desulfurase/transaminase
M896_121670	0.000586029	Inf	Cg_12A_180	UDP-N-acetylglucosamine pyrophosphorylase
M896_121680	0.002536783	0.54014371	Cg_12A_181	Man1-Src1p-C-terminal domain-containing protein
M896_121690	0.00210114	Inf	Cg_12A_182	hypothetical protein
M896_121700	0.001804589	0.62087665	Cg_12A_183	hypothetical protein
M896_121710	0.001809627	0.49452658	Cg_12A_184	putative heat shock protein
M896_121720	0.000877655	Inf	Cg_12A_185	hypothetical protein
M896_121730	0.001041667	0	Cg_12A_186	hypothetical protein
M896_121740	0.00339307	0.43573366	Cg_12A_187	hypothetical protein
M896_121750	0.002502503	0.55433393	Cg_12A_188	putative major facilitator superfamily protein
M896_121760	0.001403687	Inf	Cg_12A_189	putative major facilitator superfamily permease
M896_121770	0.003003003	Inf	Cg_12A_190	hypothetical protein
M896_121780	0.002223876	0.36137889	Cg_12A_191	hypothetical protein
M896_121790	0.002187227	Inf	Cg_12A_192	brix domain-containing protein
M896_121800	0.004221636	0.53579016	Cg_12A_193	putative membrane protein
M896_121810	0.002602977	1.16663131	Cg_12A_194	SPT5-like transcription initiation protein
M896_121820	0.001023123	Inf	Cg_12A_196	brix domain-containing protein
M896_121830	0.001868738	0.81377448	Cg_12A_197	septin-like protein
M896_121840	0.00141792	0.18511605	Cg_12A_198	hypothetical protein
M896_121850	0.001191997	0.28571429	Cg_12A_199	hypothetical protein
M896_121860	0	Inf	Cg_12A_200	putative ubiquitin-protein ligase

M896_121870	0.00318287	1.27045765	Cg_12A_201	SMC N-terminal domain-containing protein
M896_121880	0.000948148	Inf	Cg_12A_202	hypothetical protein
M896_121890	0.001141975	Inf	Cg_12A_203	kinesin motor domain-containing protein
M896_121900	0.000532141	0	Cg_12A_204	STE-like transcription factor
M896_121910	0.002043423	Inf	Cg_12A_205	hypothetical protein
M896_121920	0.001165981	Inf	Cg_12A_206	hypothetical protein
M896_121930	0.001674471	Inf	Cg_12A_208	hypothetical protein
M896_121940	0	Inf	Cg_12A_209	ribosomal protein L35
M896_121950	0.004938272	Inf	Cg_12A_210	LSM domain-containing protein
M896_140020	0.000967098	1.26529296	Cg_14_3	aminopeptidase N
M896_140030	0.001842818	0.19007581	Cg_14_4	hypothetical protein
M896_140040	0.00514353	2.12206027	Cg_14_6	hypothetical protein

Table S3. Pairwise relatedness matrix of 10 genomes of *Ordospora colligata*.

	CN-WON-2	FI-SK-17-1	FI-SKW-2-1	GB-EP-1	NO-V-7	RU-BAYA1-1	RU-KU1-2	US-SP131-1	US-SP15-1	US-SP163-1
**CN-WON-2**										
**FI-SK-17-1**	-0.2747346									
**FI-SKW-2-1**	-0.2839427	0.9205697								
**GB-EP-1**	-0.2812594	0.6635003	0.6616378							
**NO-V-7**	-0.2783259	0.6625255	0.6587074	0.7910454						
**RU-BAYA1-1**	0.6820755	-0.3170457	-0.3215586	-0.3183405	-0.3186076					
**RU-KU1-2**	-0.2192202	0.5812758	0.5773056	0.5764958	0.5588238	-0.2475983				
**US-SP131-1**	0.1586409	-0.5635653	-0.5751042	-0.570305	-0.5725328	0.1380867	-0.5096342			
**US-SP15-1**	0.1576671	-0.5696182	-0.5801566	-0.5696728	-0.5729042	0.1409453	-0.5089189	0.9831681		
**US-SP163-1**	0.1620862	-0.5656005	-0.5750715	-0.566322	-0.5682330	0.1426819	-0.5045072	0.9793122	0.983822	

Supplementary figures

Figure S1. Putative allele frequency spectrum by ploidyNGS of Ordospora colligata samples. Histograms of allele frequency of putative alleles in the BAM files found using ploidyNGS, where reads supported a single variant for most positions. Dark and light shadings of colors indicate reference and alternative allele, respectively.

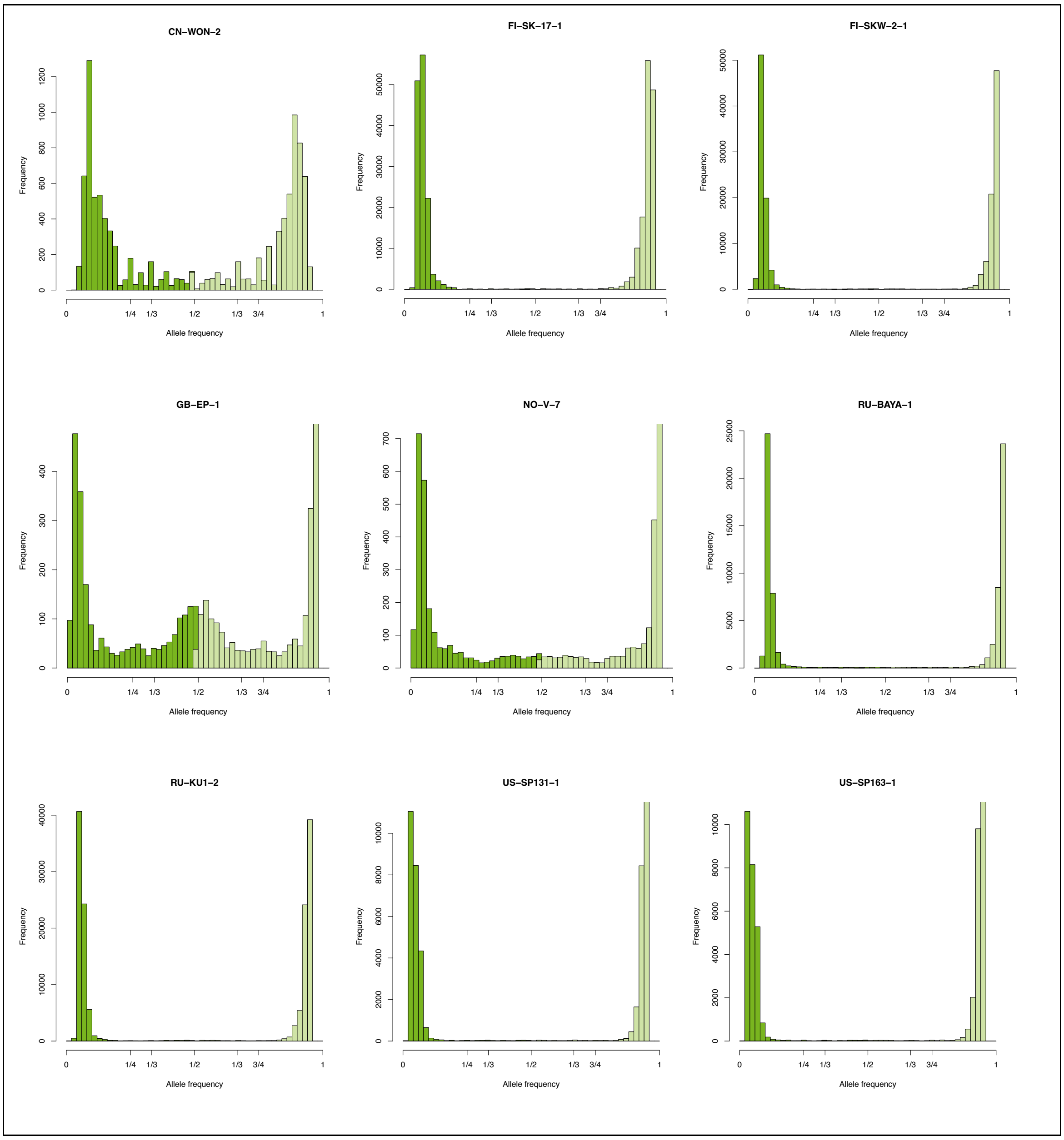

Figure S2. K-mer frequency histogram of *Ordospora colligata* by kmercountexact.sh. K-mer frequency histograms inferred using kmercountexact.sh, with a single peak for homozygous sites and no secondary peaks which would indicate di-/polyploidy.

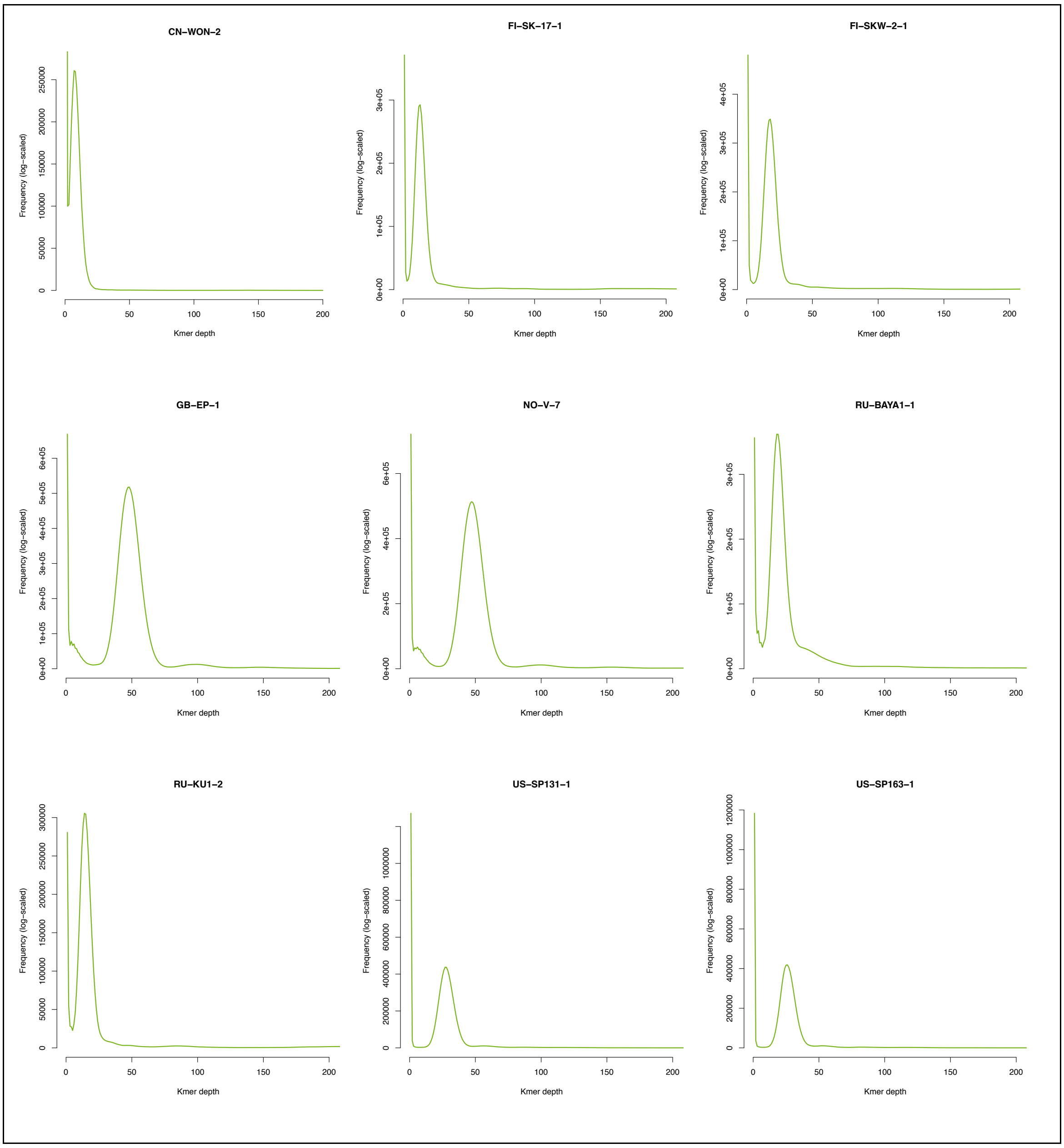

Figure S3. PCA of genomic SNP data. PC1 vs. PC2 (A) and PC1 vs. PC3 (B).

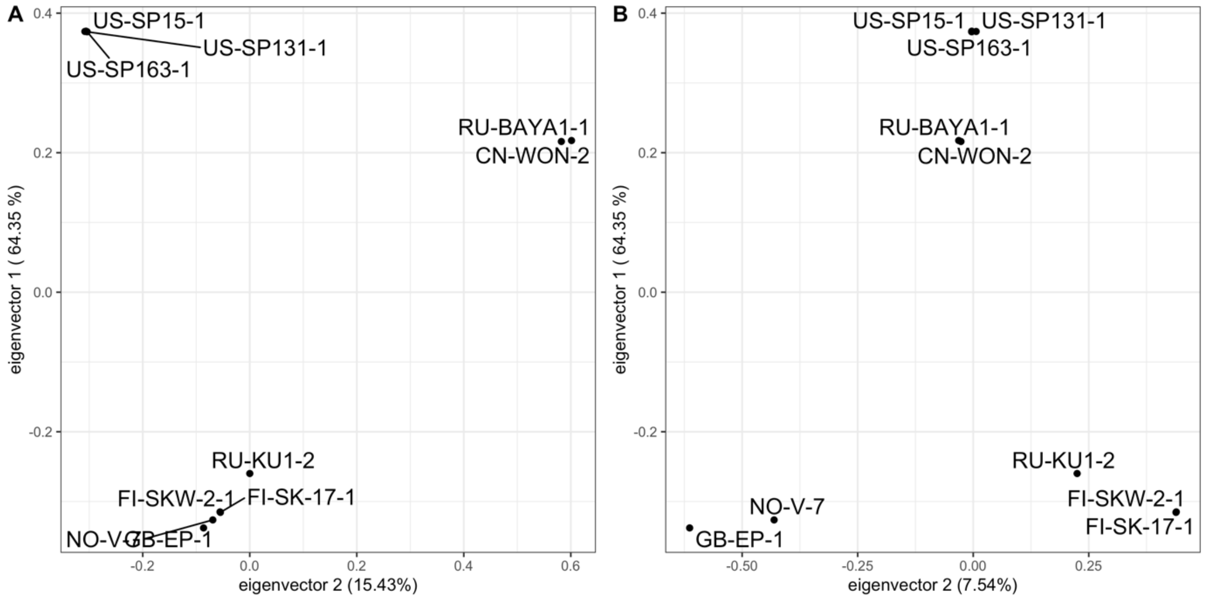

Figure S4. Cluster analysis of genomic SNP data.

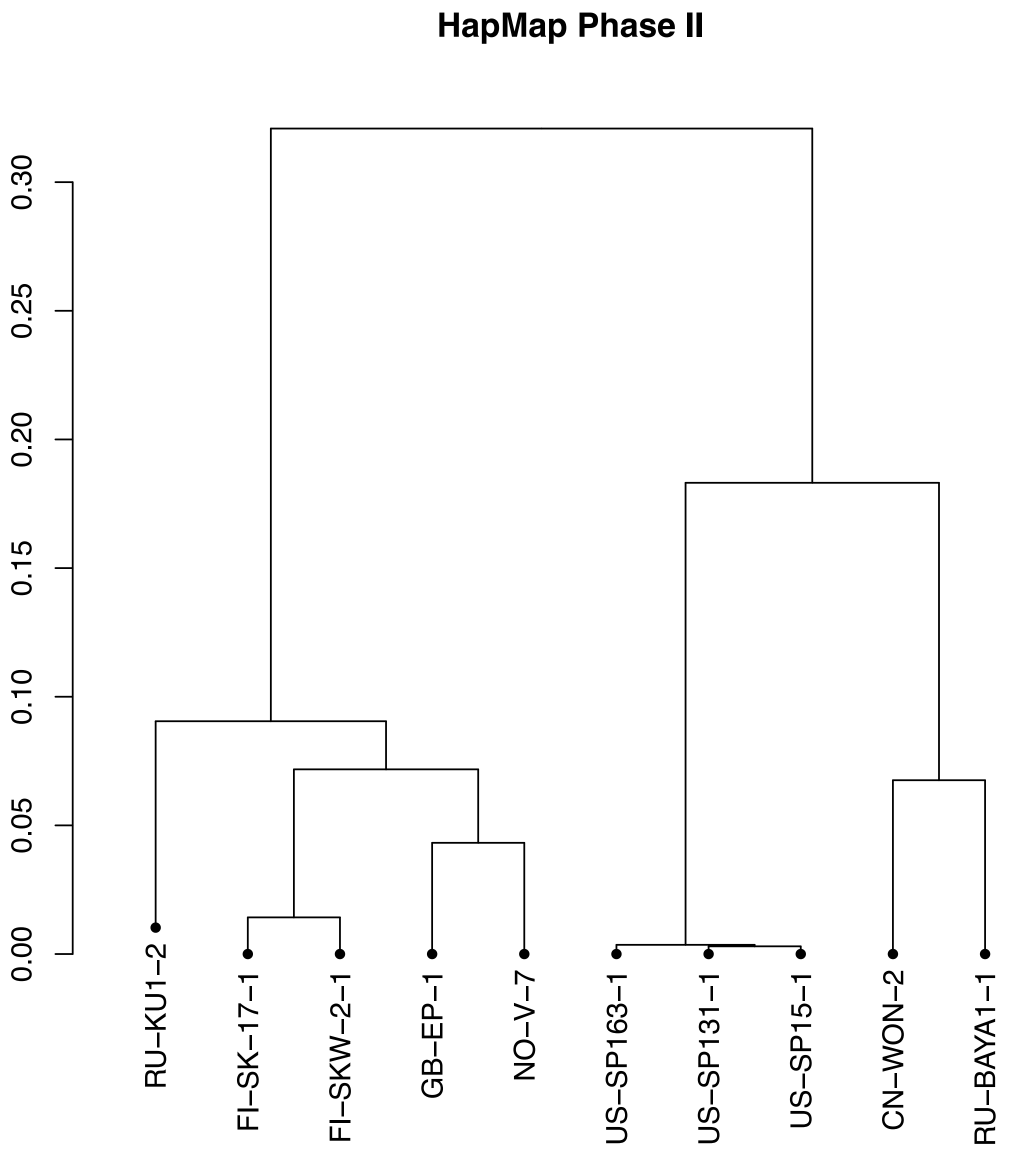

Figure S5. Admixture analysis of genomic SNP data. This pong visualization shows the most common outcomes from the ten runs for each K=2 and K=3. The colors of the K=3 chart perfectly represent the geographic distribution of the samples: Western Eurasia (red), East Asia (green) and North America (blue).

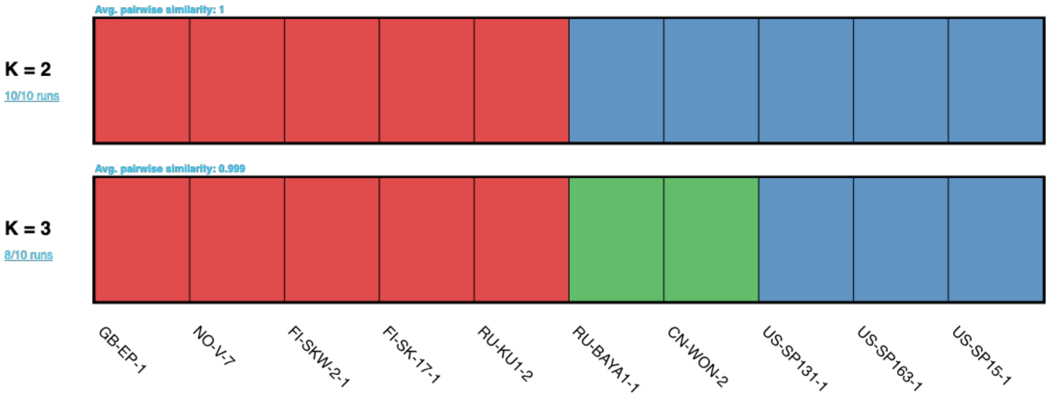

Figure S6. Bayesian tree inference of *Ordospora coligata* and the outgroup *O. pajunii* (in blue). Node labels are posterior probabilities and the scale bar unit is the divergence time as average height.

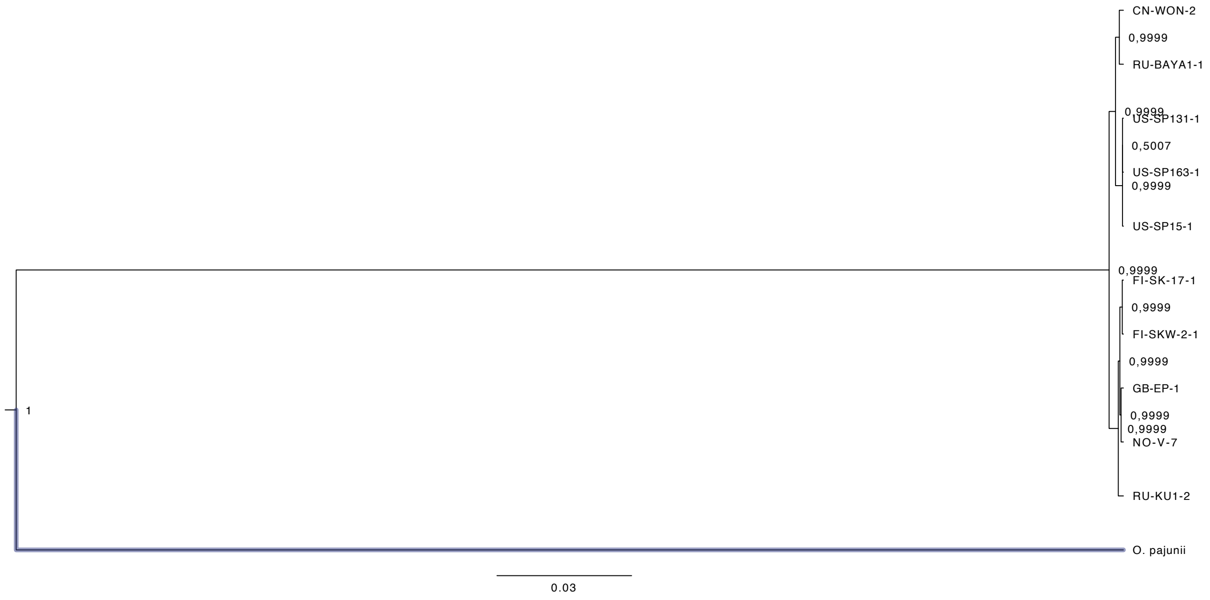

Supplement: jkad017_Supplementary_Data [file jkad017_supplementary_data.pdf]
